# Family-level stereoselective synthesis and biological evaluation of pyrrolomorpholine spiroketal natural product antioxidants†
†Electronic supplementary information (ESI) available. See DOI: 10.1039/c6sc05505b



**DOI:** 10.1039/c6sc05505b

**Published:** 2017-03-15

**Authors:** Alyssa L. Verano, Derek S. Tan

**Affiliations:** a Pharmacology Graduate Program , Weill Cornell Graduate School of Medical Sciences , Memorial Sloan Kettering Cancer Center , 1275 York Avenue, Box 422 , New York , NY 10065 , USA . Email: tand@mskcc.org; b Chemical Biology Program and Tri-Institutional Research Program , Memorial Sloan Kettering Cancer Center , 1275 York Avenue, Box 422 , New York , NY 10065 , USA

## Abstract

The pyranose spiroketal natural products pollenopyrroside A and shensongine A (also known as xylapyrroside A, *ent*-capparisine B) have been synthesized by stereoselective spirocyclizations of a common C1-functionalized glycal precursor. In conjunction with our previously reported syntheses of the corresponding furanose isomers, this provides a versatile family-level synthesis of the pyrrolomorpholine spiroketal natural products and analogues. In rat mesangial cells, hyperglycemia-induced production of reactive oxygen species, which is implicated in diabetic nephropathy, was inhibited by pollenopyrroside A and shensongine A with mid-μM IC_50_ values, while unnatural C2-hydroxy analogues exhibited more potent, sub-μM activity.

## Abstract 2

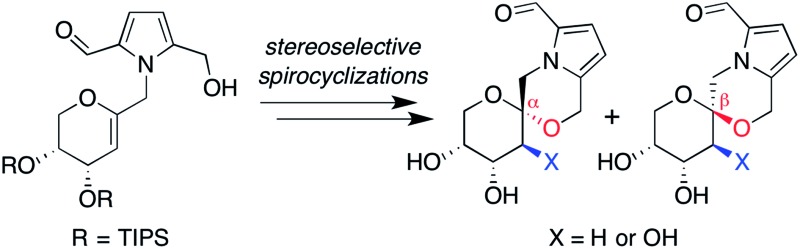
The pyranose members of the pyrrolomorpholine spiroketal family have been synthesized by stereoselective spirocyclizations of a common glycal precursor, leading to the identification of novel 2-hydroxy analogues with more potent antioxidant activities than the natural products.

## Introduction

**Fig. 1:**
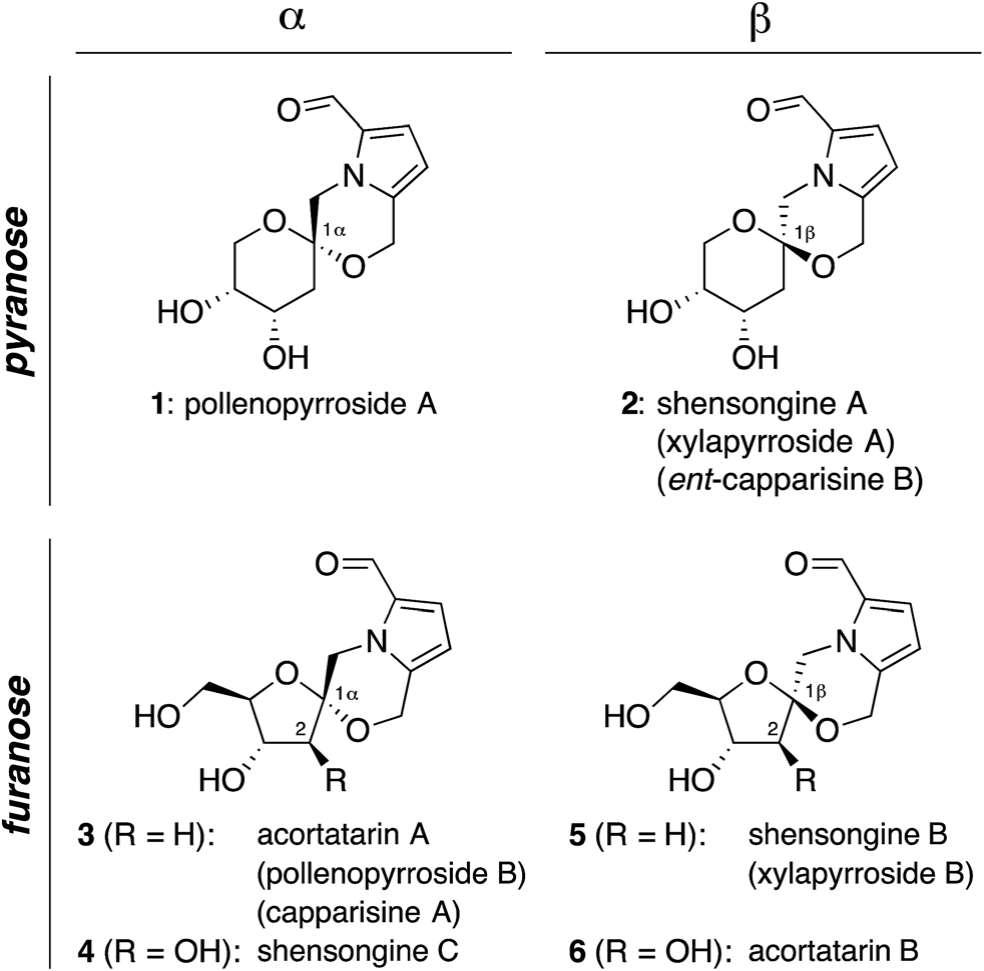
Pyrrolomorpholine spiroketal family of natural products. Structures reflect certain stereochemical revisions made subsequent to the initial isolation reports.^4^ Identical structures isolated from different natural sources and given different names (parentheses) are referred to herein by the first published name.

Pyrrolomorpholine spiroketals are a novel family of natural products that include both pyranose and furanose isomeric forms and both epimeric configurations at the anomeric carbon (Fig. 1). In 2010, three groups contemporaneously isolated acortatarins A (**3**) and B (**6**) from the rhizome of *Acorus tatarinowii*,^1^ pollenopyrrosides A (**1**) and B (**3**) from the bee-collected pollen of *Brassica campestris*,^2^ and capparisines A (**3**) and B (*ent*-**2**) from the mature fruit of *Capparis spinosa*.^3,4^ Acortatarin A and a pyranose isomer named acortatarin C (stereochemistry not assigned) were also isolated as bitter components of whole wheat bread crust.^5^

More recently, the epimeric β-spiroketals shensongine A (**2**) and shensongine B (**5**) were isolated from capsules of the antiarrhythmic Chinese herbal medicine Shensong Yangxin, along with shensongine C (**4**), a C2-hydroxy congener of acortatarin A, and pollenopyrroside B (**3**).^6^ Contemporaneously, the same β-spiroketals xylapyrrosides A (**2**) and B (**5**), were isolated from the fungus *Xylaria nigripes*.^7^

The plant-derived sources of these natural products have been used in traditional Chinese medicines for the treatment of a variety of diseases.^1–3,6,7^ Notably, acortatarins A and B exhibit antioxidant activity in a diabetic renal cell model, inhibiting production of reactive oxygen species (ROS) and significantly attenuating hyperglycemia-induced activation of NADPH oxidase and extracellular matrix production,^1,8^ hallmarks of diabetic nephropathy.^9a–c^ The xylapyrrosides also have moderate antioxidant effects and inhibit *t*-butyl hydroperoxide-induced cytotoxicity in rat vascular smooth muscle cells.^7^ Importantly, oxidative stress has been shown to play a critical role in the pathogenesis of a wide variety of disease states,^9^ due to the ability of excess ROS to inflict direct damage to essential macromolecules, as well as to lead to aberrant cell signaling.^10,11^ Accordingly, these natural products are of interest based on their therapeutic potential to treat diabetic nephropathy, cardiovascular diseases, and other pathologies in which oxidative stress is implicated.^9^ However, due to the limited quantities available from the natural sources,^12^ efficient synthetic access is required for in-depth biological and structure–activity relationship (SAR) studies. As a result, this family of natural products has attracted considerable attention from the synthetic community.^7,13^ We have previously reported concise, highly stereoselective syntheses of the furanose spiroketals acortatarins A (**3**) and B (**6**) and shensongine C (**4**) using stereoselective spirocyclizations of glycal precursors.^14^ Herein, we report expansion of this modular approach to a family-level synthesis, providing the naturally-occurring pyranose spiroketals pollenopyrroside A (**1**) and shensongine A (**2**, xylapyrroside A, *ent*-capparisine B), as well as corresponding C2-hydroxy analogues, and evaluation of the *in vitro* antioxidant activity of these compounds.

## Results and discussion

### Retrosynthetic analysis of pyrrolomorpholine spiroketal natural products

**Fig. 2:**
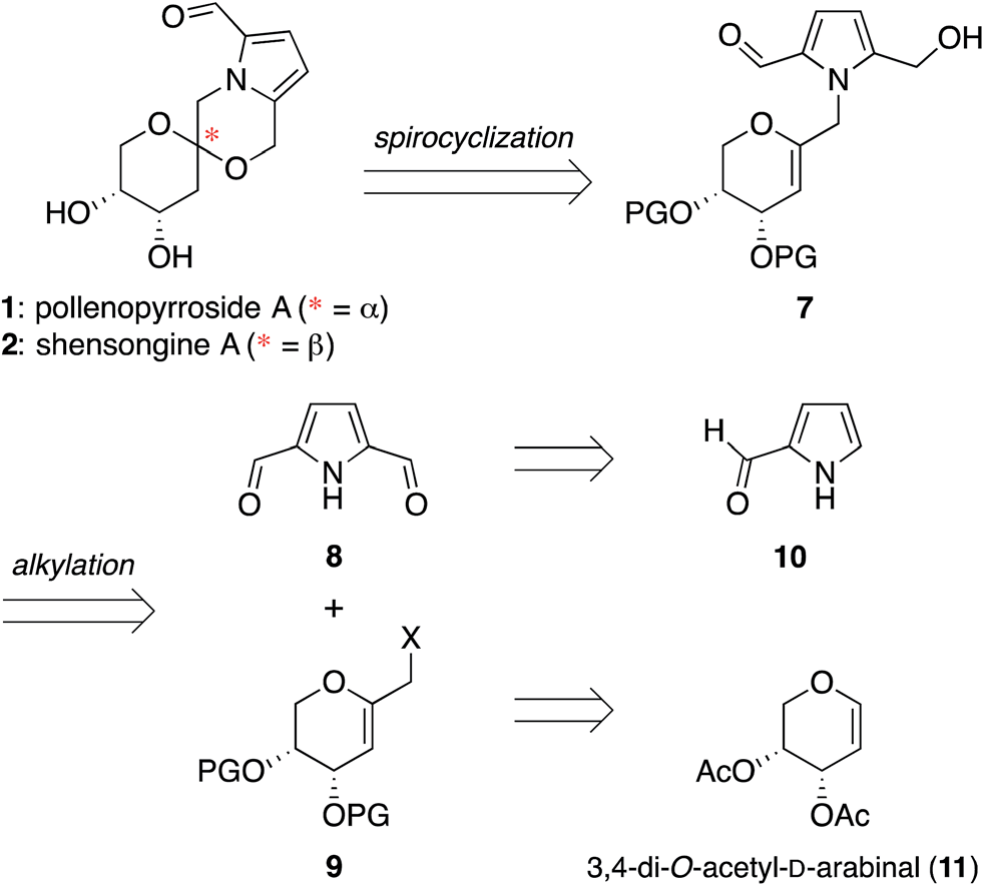
Retrosynthetic strategy *via* glycal cyclization precursor **7**.

The isomeric nature of the pyrrolomorpholine spiroketal natural products presents an attractive opportunity to develop a family-level synthesis that would provide access to both furanose and pyranose congeners, as well as both anomeric stereoisomers.^15^ Our laboratory has a long-standing interest in the stereocontrolled synthesis of spiroketals from glycals, with a particular focus on natural product scaffolds for use in probe and drug discovery.^16^ Analogous to our approach to the furanose acortatarins,^14^ we envisioned that syntheses of the corresponding pyranose isomers could be achieved by stereocontrolled spirocyclization of a pyranoglycal intermediate **7** (Fig. 2). Retrosynthetically, this glycal intermediate **7** would originate from coupling of pyrrole-2,5-dicarboxaldehyde (**8**) with pyranoarabinal (pyranoribal) derivative **9**, both of which can be accessed from commercially-available starting materials (**10**, **11**).

### Synthesis of shensongine A *via* acid-catalyzed spirocyclization of pyranoarabinal intermediate **16**

**Fig. 3:**
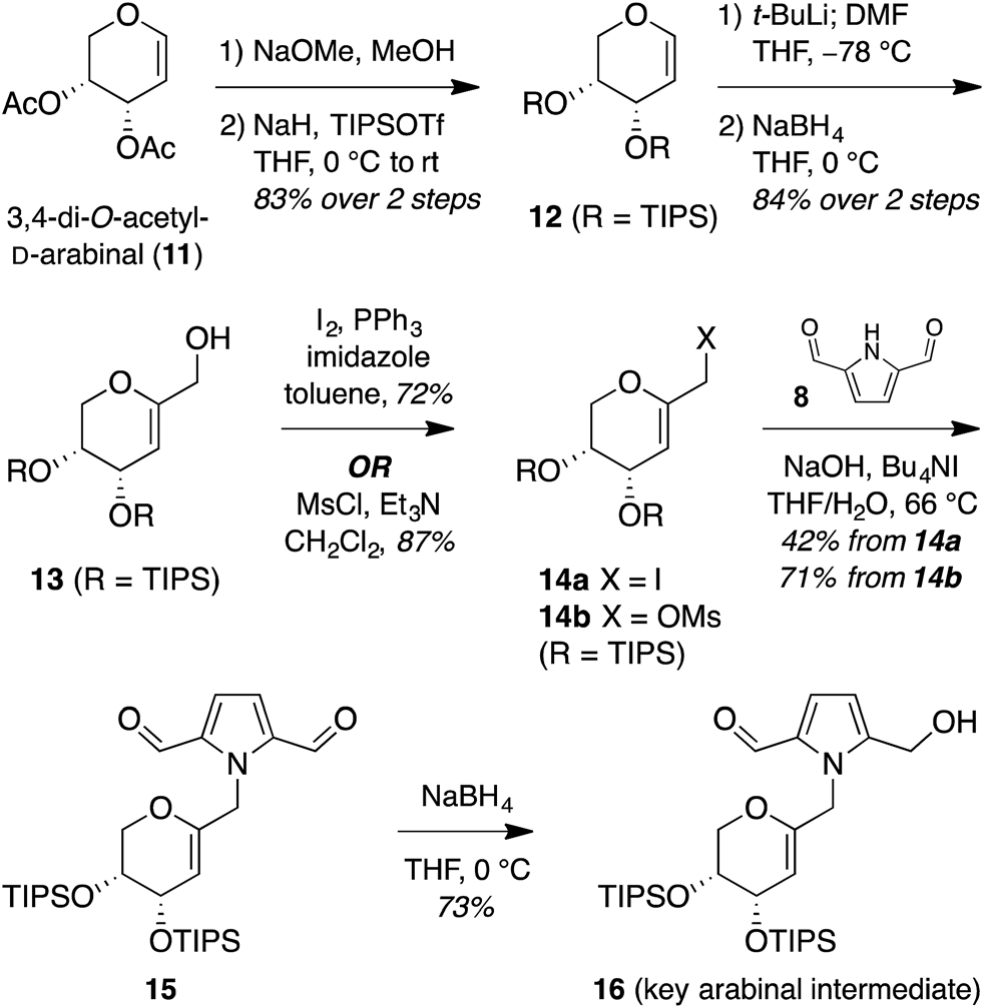
Synthesis of pivotal arabinal intermediate **16**. DMF = *N*,*N*-dimethylformamide; THF = tetrahydrofuran; TIPS = triisopropylsilyl.

Thus, we synthesized pyrrole-2,5-dicarboxaldehyde (**8**) as previously reported.^14^ To access the pyranoglycal coupling partner **14**, commercially available 3,4-di-*O*-acetyl-d-arabinal (**11**) was deacetylated^17^ then silylated^18^ to provide triisopropylsilyl-protected glycal **12** (Fig. 3). This protecting group change was necessary for compatibility with downstream C1-lithiation and also facilitated silica gel purification of intermediates. C1-Formylation^19^ of glycal **12** followed by aldehyde reduction provided protected C1-hydroxymethyl arabinal **13**. By analogy to our use of the corresponding furanose C1-iodomethyl glycal in our synthesis of the acortatarins,^14^ we proceeded with iodination of **13** to give the pyranose C1-iodomethyl glycal **14a**. However, attempted coupling of the iodide **14a** with pyrrole **8** under basic conditions led to poor yields of pyrrolomethylglycal **15**, due to the susceptibility of the C1-iodomethyl arabinal **14a** to decomposition. This is in contrast to the observed stability of the corresponding furanose C1-iodomethyl glycal in our acortatarin syntheses.^14^ However, we were able to achieve efficient coupling by instead using the more stable mesylate **14b**, which provided the desired coupling product **15** in 71% yield. Aqueous base was required for this *N*-alkylation due to the tendency of pyrrole monomers to dimerize in nonaqueous conditions.^20^ Monoreduction of the dialdehyde^21^ with NaBH_4_ gave the pivotal spirocyclization precursor **16**, from which pollenopyrroside A and shensongine A would be accessed. Careful monitoring of the reaction allowed for minimal over-reduction and minimal remaining starting material.

**Fig. 4:**
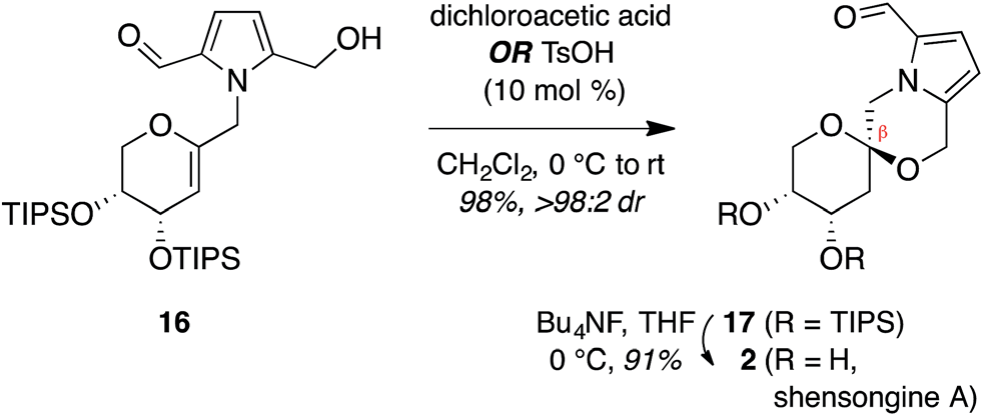
Synthesis of shensongine A (**2**, xylapyrroside A, *ent*-capparisine B).

Treatment of arabinal **16** with catalytic Brønsted acids led to exclusive formation of the thermodynamic β-spiroketal **17** (Fig. 4). Desilylation furnished shensongine A (**2**, xylapyrroside A, *ent*-capparisine B), whose optical rotation, NMR, and high-resolution mass spectral data matched those reported for the natural products.^22^

### Attempted synthesis of pollenopyrroside A *via* mercury-mediated spirocyclization of arabinal intermediates **16** and **18**

**Fig. 5:**
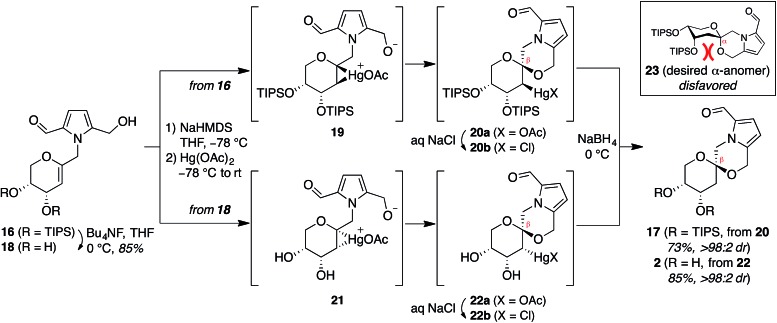
Attempted synthesis of pollenopyrroside A (1) *via* mercury-mediated spirocyclization. HMDS = hexamethyldisilazide.

In contrast, stereoselective access to the α-anomer, pollenopyrroside A, proved to be more challenging due to the inherent thermodynamic preferences of this [6,6]-spiroketal system. Formation of the contrathermodynamic α-anomer was overwhelmingly disfavored under numerous spirocyclization conditions evaluated.^22^ By analogy to our previous work in the corresponding furanoribal-derived [6,5]-spiroketal system,^14^ we first attempted Hg-mediated spirocyclization of pyranoarabinal **16**, expecting preferential *anti*-mercuration to form β-mercurinium intermediate **19**, followed by stereoinvertive cyclization to form an α-spiroketal intermediate (not shown), which could be reduced to the desired α-spiroketal **23** (Fig. 5). However, under these conditions, we instead observed exclusive formation of the β-spiroketal **17**. We attributed this undesired selectivity to steric conflicts between the bulky 3- and/or 4-*O*-TIPS groups and the morpholine ring en route to the α-anomer **23**. In addition, the 3-*O*-TIPS protecting group would preclude an intramolecular hydrogen bond with the morpholine oxygen suggested by the crystal structure of pollenopyrroside A,^2^ which may stabilize the α-configuration. To avoid these issues, we attempted Hg-mediated spirocyclization with the fully deprotected glycal substrate **18**. However, the undesired β-anomer **2** was again formed exclusively. We attributed this undesired stereoselectivity to *syn*-mercuration directed by the free C3-hydroxyl group to form the corresponding α-mercurinium intermediate **21**, followed by stereoinvertive cyclization to β-spiroketal intermediate **22a**. Isolation and NMR analysis of the intermediate 2-mercurial spiroketals as the corresponding chlorides **20b** and **22b** provided stereochemical assignments consistent with these mechanistic interpretations.^23^ Use of other mercury salts and less-hindered protecting groups, including a conformationally restricted cyclic carbonate, did not improve stereoselectivity (Table S1†).^22^

### Synthesis of pollenopyrroside A *via* metal-catalyzed spirocyclization of arabinal intermediate **18**

Based on the intramolecular hydrogen bond between the 3-hydroxyl group and morpholine oxygen postulated from the crystal structure of pollenopyrroside A,^2^ we next pursued a chelation-based approach to favor formation of the desired α-spiroketal (Table 1). Such metal-chelation approaches have been used previously to access contrathermodynamic spiroketals.^24^ Treatment of β-spiroketal **2** with various metal salts at reflux in CH_3_CN or dioxane over 24 h had no effect upon the α : β ratio (Table S2†).^22^ Treatment of the glycal spirocyclization precursor **18** with MgCl_2_, ZnCl_2_, or Ti(O-^i^Pr)_4_ led to no reaction or decomposition (Table 1, entries 1–3). However, treatment of glycal **18** with Sc(OTf)_3_ led to a 60 : 40 ratio of diastereomeric spiroketal products, favoring the desired α-anomer **1** (entry 4). We have recently used Sc(OTf)_3_ in stereoselective spirocyclizations of *exo*-glycal epoxides to form benzannulated spiroketals.^16*e*^ In those reactions, solvent played a dramatic role, with the catalyst acting as a Lewis acid in THF but as a mild source of triflic acid in CH_2_Cl_2_.^25^ To assess the mechanistic basis for the observed selectivity in this substrate system, we carried out a control experiment with DTBMP (2,6-di-*t*-butyl-4-methylpyridine) as an acid scavenger, but no reaction occurred (entry 5), suggesting that the catalyst might, indeed, be serving as a triflic acid source. However, when the reaction was carried out with TfOH alone, we observed complete diastereoselectivity for the undesired β-spiroketal (**2**) (entry 6). Moreover, no reaction was observed upon treatment with ScCl_3_ alone (entry 7). In contrast, treatment of glycal **18** with both ScCl_3_ and TfOH together (entry 8) recapitulated the 60 : 40 dr observed with Sc(OTf)_3_ (entry 4). Notably, combination of MgCl_2_ or ZnCl_2_ with TfOH also afforded a 50 : 50 mixture of the two diastereomeric spiroketals **1** and **2**. Taken together, these results suggest that the α-spiroketal **1** is formed under kinetic control, and that both a Lewis acid and Brønsted acid are required to overcome the inherent complete selectivity for the β-spiroketal **2**. This is in contrast to our previous study with *exo*-glycal epoxides, in which Sc(OTf)_3_ served as either a Lewis acid or a Brønsted acid source exclusively, depending upon solvent selection.^16*e*^ Unfortunately, the diastereomeric mixture was difficult to separate, resulting in only a 25% isolated yield of pollenopyrroside A (**1**).

**Table 1:** Metal chelation-based spirocyclization approaches to pollenopyrroside A

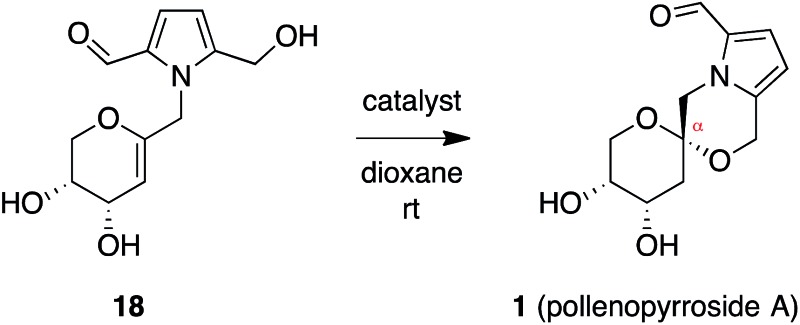			
Entry	Catalyst	Equiv.	dr (α : β)^*a*^
1	MgCl_2_	3.0	n.r.^*b*^
2	ZnCl_2_	3.0	n.r.
3	Ti(O-^i^Pr)_4_	2.0	Decomp.^*c*^
4	Sc(OTf)_3_	3.0	60 : 40^*d*^
5	Sc(OTf)_3_ + DTBMP^*e*^	3.0	n.r.
6	TfOH	0.2	0 : 100
7	ScCl_3_	3.0	n.r.
8	ScCl_3_ + TfOH^*f*^	3.0	60 : 40
9	MgCl_2_ + TfOH^*f*^	3.0	50 : 50
10	ZnCl_2_ + TfOH^*f*^	3.0	50 : 50

^*a*^Determined by ^1^H-NMR.

^*b*^n.r. = no reaction.

^*c*^Decomp. = decomposition of starting material.

^*d*^25% isolated yield of α-spiroketal **1**.

^*e*^1.0 equiv. DTBMP.

^*f*^0.5 equiv. TfOH; DTBMP = 2,6-di-*tert*-butyl-4-methylpyridine.

### Synthesis of pollenopyrroside A *via* methanol-catalyzed kinetic spirocyclization of arabinal intermediate **16**

**Fig. 6:**
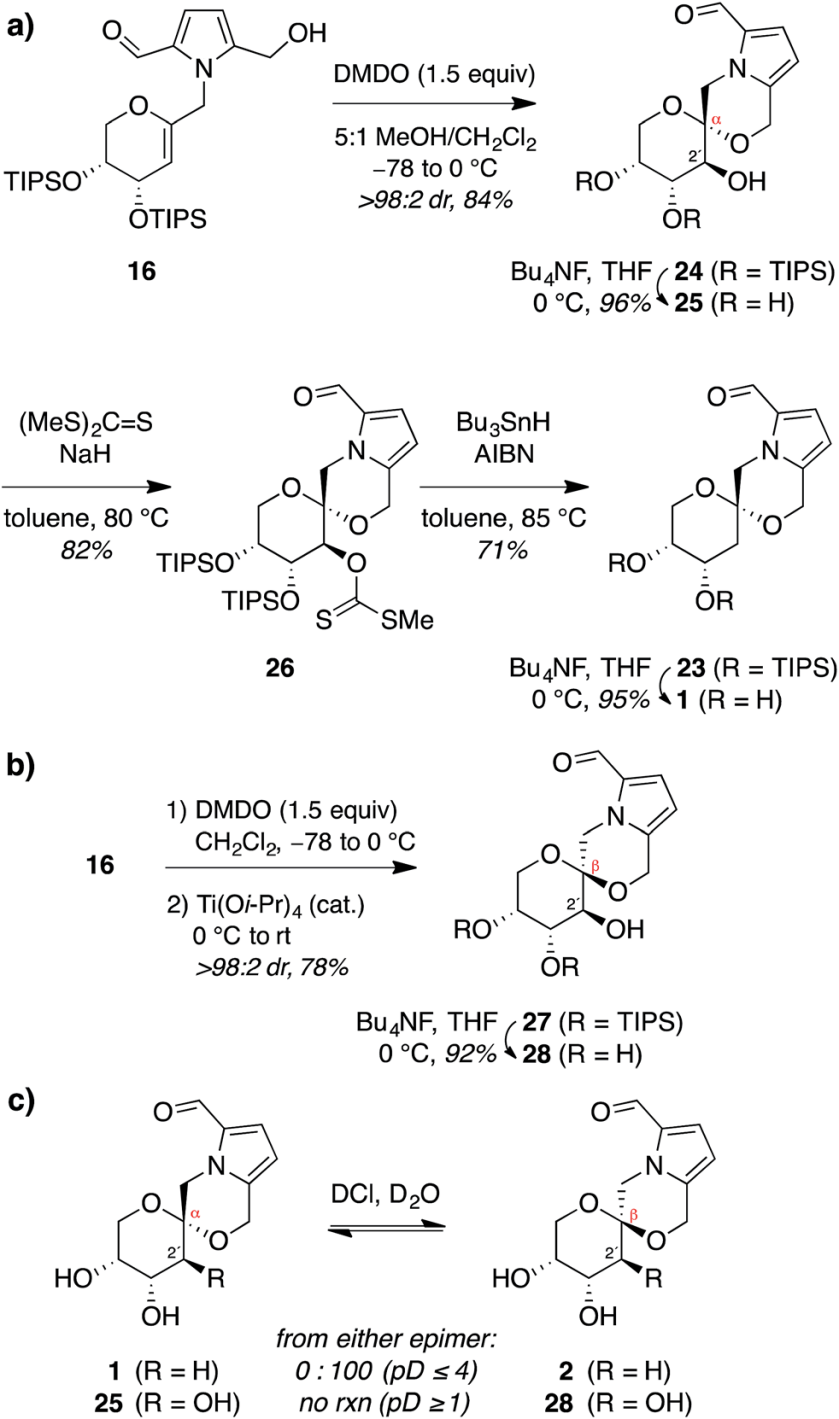
(a) Synthesis of pollenopyrroside A *via* MeOH-catalyzed kinetic spirocyclization followed by 2-deoxygenation. (b) Synthesis of 2-hydroxy analogue of shensongine A *via* Ti(O-^i^Pr)_4_-mediated kinetic cyclization. (c) Acid equilibration experiments indicate that β-spiroketal shensongine A (**2**) is thermodynamically favored over α-epimer pollenopyrroside A (**1**) in aqueous acid (pD ≤ 4) while 2-hydroxy analogues **25** and **28** do not equilibrate down to pD 1. DMDO = dimethyldioxirane; AIBN = azobisisobutyronitrile.

Thus, to provide more stereoselective access to pollenopyrroside A (**1**), we turned to our previously reported methanol-catalyzed kinetic spirocyclization of glycal epoxides.^16a,d^ While we recognized that this approach would introduce the need for deoxygenation of the resulting 2-hydroxyl group, this was balanced by the much higher stereoselectivity expected with this spirocyclization. In addition, this epoxidation–spirocyclization strategy would provide access to the non-natural 2-hydroxypyranose analogues for biological evaluation, complementing the known 2-hydroxy natural products in the furanose series (**4**, **6**). Accordingly, *anti*-epoxidation of glycal **16** with DMDO was followed by spirocyclization with inversion of configuration in methanol to give the desired α-spiroketal **24** in >98 : 2 dr (Fig. 6a). Desilylation of **24** provided a 2-hydroxy analogue of pollenopyrroside A, **25**, for biological evaluation below. Alternatively, Barton–McCombie deoxygenation^26^ of **24** at the 2-position and desilylation provided pollenopyrroside A (**1**) in 46% yield over four steps from glycal precursor **16**.

For completeness, we also carried out a stereocomplementary spirocyclization of the glycal epoxide derived from **16** using our previously established Ti(O-^i^Pr)_4_-catalyzed kinetic spirocyclization with retention of configuration^16*b*^ to provide the corresponding β-spiroketal **27** with complete β-diastereoselectivity (Fig. 6b). Desilylation then provided the 2-hydroxy analogue of shensongine A, **28**, for biological evaluation below.

### Acid equilibration studies of natural products **1** and **2** and 2-hydroxy analogues **25** and **28**

Acid equilibration experiments under aqueous conditions showed that pollenopyrroside A (**1**) was kinetically stable at pD ≥ 5, but underwent complete conversion to its C1-epimer shensongine A (**2**) at pD ≤ 4. Conversely, shensongine A was stable down to pD 1, indicating that this β-spiroketal is thermodynamically favored (Fig. 6c). In the 2-hydroxypyranose series, both **25** and its C1-epimer **28** were kinetically stable down to pD 1. Accordingly, epimerization would not be expected for any of these compounds in biological assays under physiologic conditions at pH 7.4.

### Evaluation of antioxidant activity of pyrrolomorpholine spiroketals

Previous reports have demonstrated the antioxidant activity of acortatarin A against high glucose-induced oxidative stress in rat mesangial cells^1,8^ suggesting that this scaffold may have therapeutic potential for the treatment of diabetic nephropathy. Thus, we evaluated the antioxidant activity of the complete d-enantiomeric family of furanose and pyranose pyrrolomorpholine spiroketal natural products and analogues. Intracellular ROS levels were measured using the cell-permeable 2′,7′-dichlorofluorescein diacetate (DCFH-DA), which is converted to the highly fluorescent 2′,7′-dichlorofluorescein (DCF) upon deacetylation by intracellular esterases and oxidation.^27,28^ Rat mesangial cells are highly sensitive to hyperglycemic conditions, and a significant increase in ROS was observed after 3 h exposure to 30 mM d-glucose.^22^ Inhibition of high glucose-induced oxidative stress was evaluated by concomitant treatment with the pyrrolomorpholine spiroketals, or with 1 mM *N*-acetylcysteine, a precursor of the antioxidant glutathione, as a positive control.^29^

Consistent with the previous report,^1^ acortatarins A (**3**) and B (**6**) reduced high glucose-induced ROS generation in a dose-dependent manner, with IC_50_ values of 4.6 and 11 μM, respectively, returning ROS levels to that of normal glucose conditions (Table 2, entries 2, 5). Compared to acortatarin A (**3**), the β-spiroketal shensongine B (**5**) was 4-fold less potent (entries 2, 3), suggesting that the spiroketal stereocenter may be important for activity. Consistent with this trend, the α-spiroketal shensongine C (**4**) was also more potent than its β-spiroketal congener acortatarin B (**6**) (entries 4, 5).

**Table 2:** Inhibition of high glucose-induced ROS production in rat mesangial cells^*a*^

Entry	Compound	IC_50_ (μM) [s.d. log IC_50_]^*b*^	Maximum % ROS inhibition^*c*^
1	*N*-Acetylcysteine^*d*^	n.a.	100%***
2	Acortatarin A (**3**)	4.6 [0.15]	100%***
3	Shensongine B (**5**)	19 [0.05]	100%***
4	Shensongine C (**4**)	4.8 [0.07]	100%***
5	Acortatarin B (**6**)	11 [0.07]	100%***
6	Pollenopyrroside A (**1**)	17 [0.01]	100%***
7	Shensongine A (**2**)	11 [0.05]	100%***
8	2-OH pollenopyrroside A (**25**)	0.52 [0.04]	80%***
9	2-OH shensongine A (**28**)	0.27 [0.04]	60%***

^*a*^Cells were treated with compound (0–3 mM) under normal (5.6 mM) or high (30 mM) glucose conditions and overall ROS levels were measured using the fluorescent probe DCFH-DA (50 μM).^22^

^*b*^Data are expressed as geometric mean IC_50_ (antilog [mean log IC_50_]) of three independent experiments, each performed in triplicate, with the standard deviation of the log IC_50_ shown in brackets.

^*c*^Statistical significance relative to untreated cells under high-glucose conditions was assessed using a two-tailed unpaired Student *t*-test with 95% confidence intervals; ***p ≤ 0.001.

^*d*^ *N*-Acetylcysteine (1 mM) was used as a positive control.

In the pyranose spiroketal series, both pollenopyrroside A (**1**) and shensongine A (**2**) also reduced ROS in a dose-dependent manner, with comparable IC_50_ values of 17 and 11 μM, respectively, returning ROS levels to that of normal glucose conditions (entries 6, 7). Notably, the corresponding 2-hydroxy analogues **25** and **28** were much more potent inhibitors, with IC_50_ values of 0.52 and 0.27 μM, respectively, albeit affording only partial rescues (80% and 60% maximum inhibition, respectively). Surprisingly, these analogues exhibited U-shaped dose–response curves with ROS levels actually increasing at higher concentrations (≥100 μM and ≥30 μM, respectively),^22^ possibly due to as yet undetermined feedback, off-target, or non-specific mechanisms.^30^ None of the other compounds exhibited U-shaped dose–response curves up to the maximum concentration tested (3000 μM), however, as these compounds have 9- to 70-fold higher IC_50_ values than **25** and **28**, such effects cannot be ruled out at even higher concentrations.

## Conclusions

In conclusion, we have developed an efficient, stereoselective, family-level synthesis of the pyrrolomorpholine spiroketal natural products, providing access to pollenopyrroside A (**1**) and shensongine A (**2**), as well as the corresponding 2-hydroxy analogues (**25**, **28**), from the common pyranoarabinal intermediate **16**. Pollenopyrroside A was synthesized in 11 steps and 15% overall yield from 3,4-di-*O*-acetyl-d-arabinal, and shensongine A was accessed in 9 steps and 28% overall yield. Complete diastereoselectivity was achieved for the key spiroketal-forming steps toward both natural products, as well as the 2-hydroxy congeners. This compares favorably to previous syntheses,^7,13f,g,31^ and provides practical access to the natural products and a variety of analogues. Indeed, while the synthesis of pollenopyrroside A involved an epoxidation–cyclization strategy that required subsequent C2-deoxygenation to afford the natural product, this route also provided convenient access to the corresponding 2-hydroxy analogues, which have not yet been described in the pyranose series but are known as natural products in the isomeric furanose series (*i.e.*: **4**, **6**). Evaluation of antioxidant activity against hyperglycemia-induced ROS in rat mesangial cells indicated similar activities for the anomeric natural products pollenopyrroside A (**1**) and shensongine A (**2**). Strikingly, the 2-hydroxy analogues **25** and **28** exhibited 30- to 40-fold lower IC_50_ values compared to the natural products, albeit with incomplete ROS inhibition due to U-shaped dose response curves. Notably, in the furanose series, the α-spiroketals acortatarin A (**3**) and shensongine C (**4**) were considerably more potent than the β-anomers, shensongine B (**5**) and acortatarin B (**6**), although this trend did not extend to the pyranose series (*cf.* **1**, **2**). Overall, this family-level synthesis allowed the first direct comparison of the antioxidant activities of the entire d-enantiomeric series of furanose and pyranose isomers, and led to the discovery of novel 2-hydroxy analogues with potent, sub-μM IC_50_ values. These more potent analogues may be useful for mechanistic studies, including target identification efforts. Further investigation into the mechanisms of action of these compounds and additional analogues are ongoing and will be reported in due course.
