# Supplementary material for: Family-level stereoselective synthesis and biological evaluation of pyrrolomorpholine spiroketal natural product antioxidants
Source: Chem Sci. 2017 Mar 15;8(5):3687–93. doi: 10.1039/c6sc05505b (PMC5571482; doi:10.1039/c6sc05505b)

Electronic Supplementary Material (ESI) for Chemical Science.
This journal is © The Royal Society of Chemistry 2017

Family-level stereoselective synthesis and biological evaluation of pyrrolomorpholine spiroketal natural product antioxidants

Alyssa L. Verano[a] and Derek S. Tan[a,b]*

[a] *Pharmacology Graduate Program, Weill Cornell Graduate School of Medical Sciences,*
[b] *Chemical Biology Program and Tri-Institutional Research Program*

Memorial Sloan Kettering Cancer Center,
1275 York Avenue, Box 422, New York, New York 10065, USA

Supplementary Information

A. **Supplementary Figure S1** S2
B. Complete data on mercury-mediated spiroketalizations of glycals (**16**, **18**) S3
C. Complete data on metal chelation-based spiroketal equilibration S4
D. Acid equilibration of pyranose spiroketals (**1**, **2**, **25**, **28**) S6
E. Complete data on antioxidant evaluation of spiroketals (**1–6**, **25**, **28**) S7
F. Background on structural assignments of natural products S9
G. Materials and methods S11
H. Synthesis of *C*1-mesyl-hydroxymethyl-D-arabinal (**14b**) S12
I. Synthesis of *C*1-pyrrolomethyl-D-arabinal substrates (**15**, **16**, **18**) S15
J. Synthesis of 2-mercurial spiroketals (**20b**, **22b**) S17
K. Synthesis of shensongine A (**2**) S19
L. Synthesis of pollenopyrroside A (**1**) S21
M. Synthesis of 2-hydroxy analogues (**25**, **28**) S24
N. Comparison of synthetic and authentic natural products (**1**, **2**) S26
O. Dose-response curves and bar graphs for antioxidant activity studies S31
P. ^{1}H-NMR and ^{13}C-NMR spectra S39

A. SUPPLEMENTARY FIGURE S1

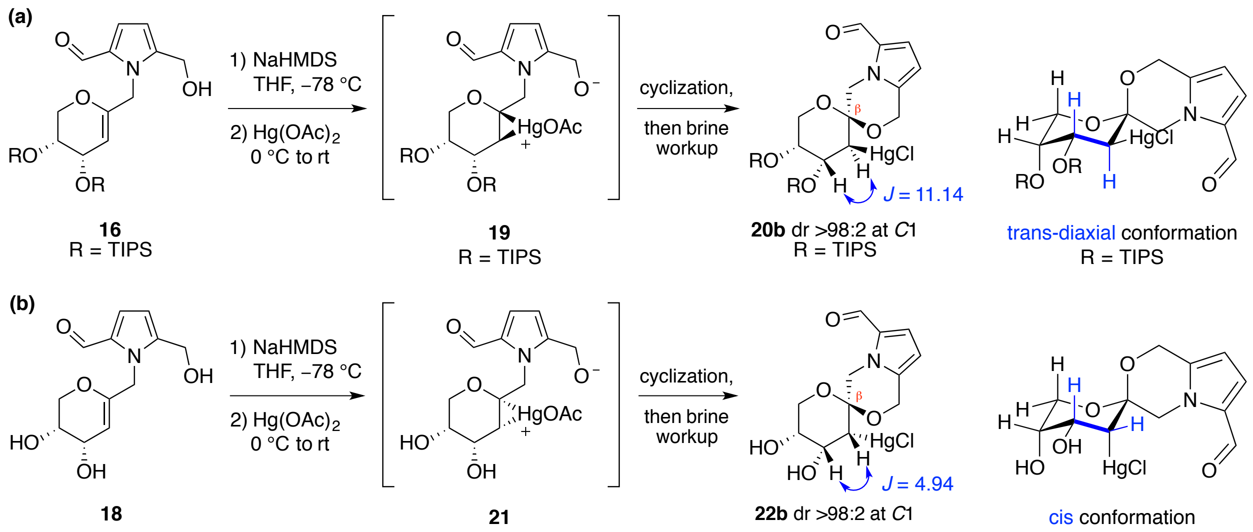

Figure S1. Isolation of α-mercurial acetal intermediates provides mechanistic evidence for mercury-mediated spirocyclizations of arabinals 16 and 18. (**a**) *Anti*-mercuration of **16**, followed by spirocyclization with retention of configuration, leads to 2-mercurial spiroketal **20b**. (**b**) *Syn*-mercuration of **18**, followed by S_N2 spirocyclization, leads to 2-mercurial spiroketal **22b**. See SECTION J below for analytical data. HMDS = hexamethyldisilazane; TIPS = triisopropylsilyl.

B. Complete data on Hg-mediated spiroketalizations of glycals

Table S1. Complete data for spirocyclizations of monoalcohol glycals 16, 18, and S1.

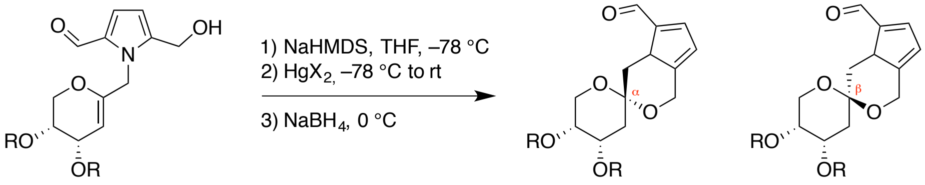

16 R = TIPS
18 R = H
S1 R,R = C=O

23 R = TIPS
1 R = H
S1-α R,R = C=O

17 R = TIPS
2 R = H
S1-β R,R = C=O

entry	substrate	reagent[a]	t (h)	dr (α:β)[a]
1	**16**	$Hg(OAc)_2$	1.5	0:100
2	**16**	$Hg(OAc)_2$	0.5	0:75[b]
3	**16**	$Hg(OAc)_2$	3	0:100
4	**16**	$Hg(OAc)_2$	6	0:100
5	**16**	$Hg(OAc)_2$	24	0:100
6	**16**	$HgCl_2$	1.5	0:87[b]
7	**16**	$Hg(TFA)_2$	1.5	0:100
8	**18**	$Hg(OAc)_2$	1.5	0:100
9	**S1**	$Hg(OAc)_2$	1.5	0:90[b]
10	**S1**	$Hg(OAc)_2$	0.5	0:75[b]
11	**S1**	$Hg(OAc)_2$	3	decomp.[c]
12	**S1**	$Hg(OAc)_2$[d]	3	decomp

[a] Determined by ^{1}H-NMR; [b] Remainder of product ratio was starting material; [c] decomp. = decomposition of starting material and/or product; [d] No pretreatment with NaHMDS. HMDS = hexamethyldisilazane; TFA = trifluoroacetate.

Variation of reaction time and mercury(II) salts had no effect upon α:β dr, and only β-spiroketal was observed for all reaction conditions attempted.

Attempts to access the desired α-spiroketal via mercury-mediated spirocyclization of conformationally restricted cyclic carbonate **S1** were unsuccessful, leading to β-spiroketal or decomposition. Additionally, attempted spiroketalization of **S1** with acid (dichloroacetic acid, TsOH, trifluoroacetic acid, AcOH) led to decomposition of starting material.

C. COMPLETE DATA ON METAL CHELATION-BASED SPIROKETAL EQUILIBRATION

Table S2. Complete data for metal chelation-based equilibration of β-spiroketal 2

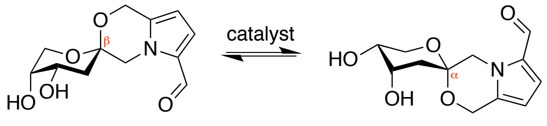

2 (shensongine A) **1** (pollenopyrroside A)

entry	catalyst[a]	equiv.	solvent	t (h)	dr **2** (β) : **1** (α)[b]
1	$MgCl_2$	3.0	CH_3CN	24	n.r.[c]
2	$ZnCl_2$	3.0	CH_3CN	24	n.r.
3	$MgCl_2$ + TFA[d]	3.0	CH_3CN	24	n.r.
4	$ZnCl_2$ + TFA[d]	3.0	CH_3CN	24	100:0
5	Ti(O*i*-Pr)$_4$	2.0	CH_3CN	24	decomp.[e]
6	Sc(OTf)$_3$	3.0	CH_3CN	24	100:0
7	$ScCl_3$	3.0	CH_3CN	24	100:0
8	$Ca(ClO_4)_2$, $HClO_4$	3.0	CH_3CN	24	100:0
9	$MgCl_2$	3.0	dioxane	24	100:0
10	$ZnCl_2$	3.0	dioxane	24	100:0
11	$MgCl_2$ + TFA[d]	3.0	dioxane	24	100:0
12	$ZnCl_2$ + TFA[d]	3.0	dioxane	24	100:0
13	Ti(O*i*-Pr)$_4$	2.0	dioxane	24	decomp.
14	Sc(OTf)$_3$	3.0	dioxane	24	100:0
15	$ScCl_3$	3.0	dioxane	24	100:0
16	$Ca(ClO_4)_2$, $HClO_4$	3.0	dioxane	24	100:0

[a] rt to refluxing in indicated solvent (82 °C in acetonitrile, 101 °C in dioxane); [b] Determined by ^{1}H-NMR; [c] n.r. = no reaction; [d] 1.0 equiv of TFA; [e] decomp. = decomposition of starting material. TFA = trifluoroacetic acid.

Previous studies[1,2] have shown that contrathermodynamic spiroketals can be accessed by treatment of spiroketals with Lewis acids, particularly metal salts that can participate in internal chelates with an axial hydroxyl and spiroketal oxygen, thereby overriding the thermodynamic preferences of the system. Based on the intramolecular hydrogen bond between the 3´-hydroxyl and the morpholine oxygen postulated from the crystal structure of pollenopyrroside A, we attempted isomerization of β-spiroketal **2** with metal salts, including those successfully used by Evans *et al.*[3] and Smith *et al.*[4] to access contrathermodynamic spiroisomers. Unfortunately, all conditions failed to yield the desired α-spiroketal **1** via equilibration of **2**.

[1] D. R. Williams, P. A. Jass and R. D. Gaston, *Tetrahedron Lett.*, 1993, **34**, 3231–3234.
[2] M. J. Kurth, E. G. Brown, E. Hendra and H. Hope, *J. Org. Chem.*, 1985, **50**, 1115–1117.
[3] D. A. Evans, B. W. Trotter, P. J. Coleman, B. Côté, L. C. Dias, H. A. Rajapakse and A. N. Tyler, *Tetrahedron*, 1999, **55**, 8671–8726.
[4] A. B. Smith, V. A. Doughty, Q. Lin, L. Zhuang, M. D. McBriar, A. M. Boldi, W. H. Moser, N. Murase, K. Nakayama and M. Sobukawa, *Angew. Chem. Int. Ed.*, 2001, **40**, 191–195.

Table S3. Complete data for attempted metal chelation-based cyclizations of glycal 18 towards α-spiroketal 1

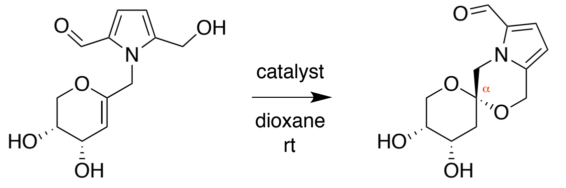

18 **1** (pollenopyrroside A)

Entry	Table 1 entry	Catalyst	Equiv	t (h)	dr (α:β)[a]
1		$MgCl_2$	3.0	1	n.r.[b]
2	1	$MgCl_2$	3.0	3	n.r.
3		$MgCl_2$	3.0	24	n.r.
4		$ZnCl_2$	3.0	1	n.r.
5	2	$ZnCl_2$	3.0	3	n.r.
6		$ZnCl_2$	3.0	24	n.r.
7		Ti(O*i*-Pr)$_4$	2.0	1	n.r.
8	3	Ti(O*i*-Pr)$_4$	2.0	3	decomp.[c]
**9**	**4**	**Sc(OTf)$_3$**	**3.0**	**3**	**60:40**[d]
10		Sc(OTf)$_3$	3.0	1.5	50:25[e]
11		Sc(OTf)$_3$	3.0	0.5	30:20[e]
12		Sc(OTf)$_3$	3.0	4	50:50
13	5	Sc(OTf)$_3$ + DTBMP[f]	3.0	3	n.r.
14	6	TfOH	0.5	3	0:100
15		TfOH	0.2	3	0:100
16	7	$ScCl_3$	3.0	3	n.r.
17	8	$ScCl_3$ + TfOH[g]	3.0	3	60:40
18	9	$MgCl_2$ + TfOH[g]	3.0	3	50:50
19	10	$ZnCl_2$ + TfOH[g]	3.0	3	50:50

[a] Determined by ^{1}H-NMR; [b] n.r. = no reaction; [c] decomp. = decomposition of starting material; [d] 25% isolated yield of α-spiroketal **1**; [e] Remainder of product ratio was starting material; [f] 1.0 equiv of DTBMP; [g] 0.5 equiv of TfOH. DTBMP = 2,6-di-*tert*-butyl-4-methylpyridine.

Kinetic spirocyclization of **18** was attempted using a variety Lewis acids. No reaction was observed with $MgCl_2$, $ZnCl_2$, and Ti(O*i*-Pr)$_4$ alone (entries 1–8). Treatment with Sc(OTf)$_3$ for 3 h (entry 9) led to a promising α:β ratio of 60:40. This α:β dr was maintained with shorter reaction times, but incomplete conversion was observed (entries 10–11), while a longer reaction time led to loss of diastereoselectivity (entry 12). Collectively, control experiments (entries 13–17) suggest that trace triflic acid is necessary for cyclization, and Sc(III) may contribute to the observed α:β diastereoselectivity with Sc(OTf)$_3$.

D. ACID EQUILIBRATION OF PYRANOSE SPIROKETALS (1, 2, 25, 28)

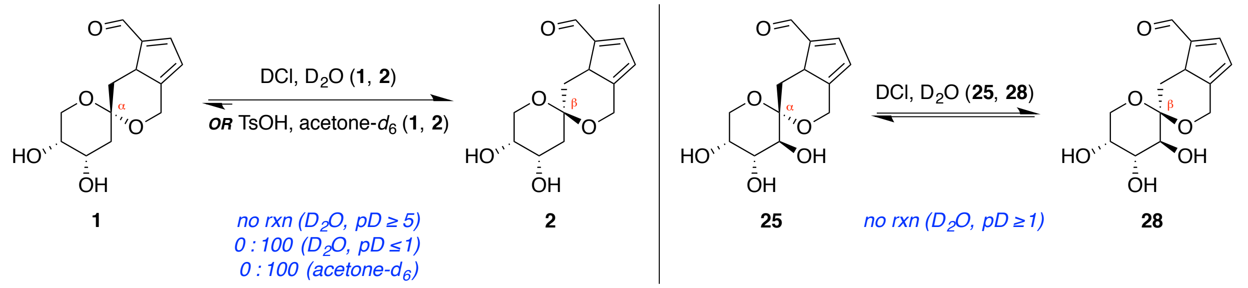

Figure S2. Acid equilibration of pollenopyrroside A, shensongine A, and their 2-hydroxy analogues. Equilibration of pollenopyrroside A (**1**) and its *C*1-epimer shensongine A (**2**) was studied in D_2O and acetone-d_6; equilibration of 2-hydroxy pyranose analogues (**25**, **28**) was studied in D_2O. For the natural products **1** and **2**, acid equilibration favored the β-spiroketal completely (0:100 α/β) in D_2O (pD ≤ 4) and acetone-d_6, indicating that the β-spiroketal is thermodynamically favored. For the 2-hydroxy pyranose series, thermodynamic preferences in D_2O could not be assessed because both **25** and its *C*1-epimer **28** were kinetically stable down to pD 1, where gradual decomposition occurred over time.

D_2O: Spiroketals **1** and **2** were dissolved individually in 0.6 mL D_2O and treated with deuterium chloride and the pD was lowered sequentially until epimerization was observed by NMR. α-Spiroketal **1** was stable until reaching pD 4, where complete epimerization to β-spiroketal **2** was observed after 15 min, and the 0:100 α/β ratio was unchanged after 3 h at a final pD 1, indicating that the β-spiroketal is thermodynamically favored under these conditions. No furanose or hydrolysis products were observed, and longer reaction times resulted in gradual decomposition.

Spiroketals **25** and **28** were dissolved individually in 0.6 mL D_2O and treated with deuterium chloride to a final pD 1 at rt for 3 h. In both cases, no equilibration was observed, indicating kinetic stability under these conditions. No furanose or hydrolysis products were observed, and longer reaction times resulted in gradual decomposition. Notably, this kinetic stability in D_2O was observed for the 2-hydroxy furanose congeners as well, as reported previously.[5]

pD values were recorded using a Fisher Scientific Accumet Excel XL60 dual channel pH/ion/conductivity/DO meter kit with a glass pH electrode (Fisher model: 13-636-XL60), and corrected using the equation ***pD = pH meter reading + 0.40***.[6]

acetone-d_6: Spiroketals **1** and **2** were dissolved individually in 0.6 mL D_2O and treated with *p*-toluenesulfonic acid (0.10 equiv) at rt for 10 min. In each case, the β-spiroketal was the only product observed, indicating that the β-spiroketal is thermodynamically favored under these conditions. No furanose or hydrolysis products were observed, and longer reaction times resulted in gradual decomposition.

[5] J. M. Wurst, A. L. Verano and D. S. Tan, *Org. Lett.*, 2012, **14**, 4442–4445.
[6] (a) P. K. Glasoe and F. A. Long, *J. Phys. Chem.*, 1960, **64**, 188–190. (b) R. Lumry, E. L. Smith and R. R. Glantz, *J. Am. Chem. Soc.*, 1951, **73**, 4330–4340.

E. Complete data on antioxidant evaluation of pyrrolomorpholine spiroketals

Antioxidant activity assay[7,8,9]

The inhibitory effects of the pyrrolomorpholine spiroketals on high glucose-induced oxidative stress were evaluated in rat mesangial cells (RMC). The rat mesangial cells, obtained from the American Type Culture Collection (CRL-2573), were cultured at 37 °C in Dulbecco's modified Eagle's medium (DMEM) containing 5.6 mM glucose (normal glucose), 10% fetal calf serum (FCS), nonessential amino acids, penicillin/streptomycin under a humidified atmosphere of 95% air and 5% CO_2. Rat mesangial cells were seeded in 96-well plates approximately 24 hours before the start of all experiments.

For each experiment, rat mesangial cells were incubated with pyrrolomorpholine spiroketal compound (0.01, 0.03, 0.1, 0.3, 1, 3, 10, 30, 100, 300 μM, 1 mM, 3 mM) or 1 mM *N*-acetyl cysteine (NAC, positive control) in the presence of either 5.6 mM (normal glucose, NG) or 30 mM (high glucose, HG) D-glucose for 3 h. RMC were then incubated with 50 μM dichloro-dihydro-fluorescein diacetate (DCFH-DA) for 45 min, and intracellular production of ROS was detected by 2′,7′-dichlorofluorescein (DCF) fluorescence (485 nm excitation, 530 emission) as measured by a microplate reader (SpectraMax M5/M5, Molecular Devices). Values are expressed as percentage of ROS inhibition, where fluorescence from untreated cells under normal glucose conditions was set at 0% and untreated cells under high glucose conditions was set at 100%.

The MTT viability assay was performed in every experiment to assess the cellular toxicity of each compound. The results showed that all compounds were non-toxic up to 3 mM over 72 h (data not shown).

---

7 H. Wang and J. A. Joseph, *Free Radical Biol. Med.*, 1999, **27**, 612–616.
8 L. M. Magalhães, M. A. Segundo, S. Reis and J. L. F. C. Lima, *Anal. Chim. Acta.*, 2008, **613**, 1–19.
9 D. Giustarini, I. Dalle-Donne, D. Tsikas and R. Rossi, *Crit. Rev. Clin. Lab.*, 2009, **46**, 241–281.

Table S4. Antioxidant activity of pyrrolomorpholine compounds against high glucose-induced oxidative stress in rat mesangial cells. Each compound was tested in three independent experiments (biological replicates), with each experiment performed in triplicate (technical replicates).

See SECTION O below for all dose-response curves and bar graphs for antioxidant activity studies.

Entry	Compound	log [IC_{50} (μM)] ± SD (technical replicates)	mean log [IC_{50} (μM)] ± SD (biological replicates)	antilog[mean [logIC_{50} (μM)]] (geometric mean IC_{50}, μM)
1a	acortatarin A (**3**)	0.73 ± 0.05	0.66 ± 0.15	4.57
b		0.76 ± 0.05		
c		0.49 ± 0.04		
2a	shensongine B (**5**)	1.26 ± 0.04	1.28 ± 0.05	19.16
b		1.34 ± 0.04		
c		1.25 ± 0.03		
3a	shensongine C (**4**)	0.64 ± 0.06	0.69 ± 0.07	4.84
b		0.66 ± 0.05		
c		0.76 ± 0.04		
4a	acortatarin B (**6**)	1.02 ± 0.07	1.03 ± 0.07	10.70
b		1.10 ± 0.05		
c		0.97 ± 0.05		
5a	pollenopyrroside A (**1**)	1.23 ± 0.04	1.22 ± 0.01	6.58
b		1.21 ± 0.04		
c		1.22 ± 0.02		
6a	shensongine A (**2**)	1.12 ± 0.06	1.06 ± 0.05	11.36
b		1.02 ± 0.07		
c		1.03 ± 0.04		
7a	2-OH pollenopyrroside A (**25**)	–0.33 ± 0.05	-0.29 ± 0.04	0.52
b		–0.24 ± 0.03		
c		–0.29 ± 0.08		
8a	2-OH shensongine A (**28**)	–0.53 ± 0.04	-0.58 ± 0.04	0.27
b		–0.61 ± 0.07		
c		–0.59 ± 0.07		

F. BACKGROUND ON STRUCTURAL ASSIGNMENTS OF NATURAL PRODUCTS

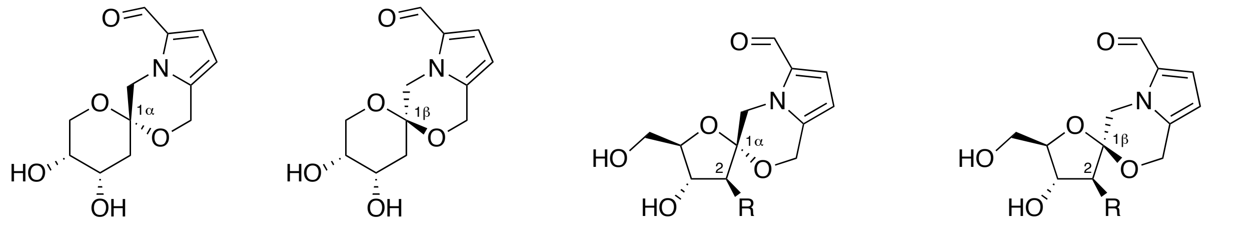

1: pollenopyrroside A

2: shensongine A (xylapyrroside A) (*ent*-capparisine B)

3 (R = H): acortatarin A (pollenopyrroside B) (capparisine A)
4 (R = OH): shensongine C

5 (R = H): shensongine B (xylapyrroside B)
6 (R = OH): acortatarin B

Pyrrolomorpholine spiroketals are a novel family of natural products that include both pyranose and furanose isomeric forms and both epimeric configurations at the anomeric carbon (**Fig. 1**). In 2010, three groups contemporaneously isolated acortatarins A and B from the rhizome of *Acorus tatarinowii*,[10] pollenopyrrosides A and B from the bee-collected pollen of *Brassica campestris*,[11] and capparisines A and B from the mature fruit of *Capparis spinosa*.[12] The D-configuration of pollenopyrroside A (**1**) was assigned unambiguously by single-crystal X-ray diffraction analysis with CuKα radiation,[11] which enables assignment of absolute stereochemistry (Flack parameter = 0.06(2)).[13] Acortatarin A and capparisine A were initially assigned identical structures with an L-configuration, while pollenopyrroside B was assigned the enantiomeric D-configuration (**3**) by analogy to pollenopyrroside A (**1**). The first total syntheses in this family were reported by Sudhakar and coworkers for acortatarins A (**3**) and B (**6**), resulting in revision of both original stereochemical assignments.[14] Acortatarin A was misassigned as the L-configuration based on Mosher ester analysis that did not account for reversal of Cahn–Ingold–Prelog configuration between the precursor acid chloride and product ester.[10] As a result, acortatarin B was misassigned as the L-configuration by analogy to acortatarin A. Capparisine A was apparently assigned the L-configuration arbitrarily, as MoKα radiation was used in X-ray crystallographic analysis, which cannot be used to assign absolute stereochemistry (Flack parameter = 10(10).[12] In contrast, the direction of optical rotation of capparisine A ($[\alpha]^{25}_{D}$ +34.5 (c 0.13, MeOH)) matches that of acortatarin A ($[\alpha]^{27}_{D}$ +178.4 (c 0.4, MeOH)) and pollenopyrroside B ($[\alpha]^{20}_{D}$ +242.7 (c 0.08, MeOH)), the D-configuration of which has been confirmed by total synthesis and direct comparison to an authentic sample of acortatarin A.[5,14] Thus, acortatarin A and pollenopyrroside B are now known to be identical,[5] and the same D-configuration can be assigned to capparisine A. In addition, the D-configuration of acortatarin B was confirmed by total synthesis, X-ray crystallography, and direct comparison to an authentic sample of acortatarin B.[5,14] Acortatarin A and a pyranose isomer named acortatarin C (stereochemistry not assigned) were also isolated as bitter components of whole wheat bread crust.[15]

[10] X. Tong, L. Zhou, Y. Wang, C. Xia, Y. Wang, M. Liang, F. Hou and Y. Cheng, *Org. Lett.*, 2010, **12**, 1844–1847.
[11] J. Guo, Z. Feng, Y. Yang and Z. Zhang, *Chem. Pharm. Bull.*, 2010, **58**, 983–985.
[12] T. Yang, C. Wang, G. Chou, T. Wu, X. Cheng and Z. Wang, *Food Chemistry*, 2010, **123**, 705–710.
[13] S. Parsons, H. D. Flack and T. Wagner, *Acta. Crystallogr., Sect. B: Struct. Sci. Cryst. Eng. Mater.*, 2013, **69**, 249–259.
[14] G. Sudhakar, V. D. Kadam, S. Bayya, G. Pranitha and B. Jagadeesh, *Org. Lett.*, 2011, **13**, 5452–5455.
[15] D. Jiang and D. G. Peterson, *Food Chemistry*, 2013, **141**, 1345–1353.

More recently, the epimeric β-spiroketals shensongine A (**2**) and shensongine B (**5**) were isolated from capsules of the antiarrhythmic Chinese herbal medicine Shensong Yangxin, along with shensongine C (**4**), a *C*2-hydroxy congener of acortatarin A, and pollenopyrroside B (**3**).[16] The absolute configurations of shensongines B (**4**) and C (**5**) were assigned by comparison to optical rotations of synthetic material reported earlier by Sudhakar as *C*1-epimers of the acortatarins.[14] Shensongine A (**2**) ($[a]^{27}_{D}$ –12.7 (*c* 0.05, MeOH))[16] was assigned as the enantiomer of capparisine B ($[a]^{25}_{D}$ +37.9 (*c* 0.09, MeOH))[12] based on opposite sign of optical rotation. Contemporaneously, the same β-spiroketals xylapyrrosides A (**2**) and B (**5**), were isolated from the fungus *Xylaria nigripes*.[17] Xylapyrroside A ($[\alpha]^{25}_{D}$ –189 (*c* 0.1, MeOH)) was assigned the D-configuration based on single-crystal X-ray diffraction analysis with CuKα radiation (Flack parameter = 0.19(18)), again with an opposite sign of optical rotation relative to that reported for capparisine B. It should be noted that synthesis of **2** from D-fructose was reported to afford material with the same sign of optical rotation as the capparisine B,[18] although a separate synthesis of **2** from 2-deoxy-D-ribose reported the opposite sign,[19] consistent with the analysis above and results herein.

[16] B. Ding, Y. Dai, Y.-L. Hou and X.-S. Yao, *J. Asian Nat. Prod. Res.*, 2015, **17**, 559–566.
[17] M. Li, J. Xiong, Y. Huang, L.-J. Wang, Y. Tang, G.-X. Yang, X.-H. Liu, B.-G. Wei, H. Fan, Y. Zhao, W.-Z. Zhai and J.-F. Hu, *Tetrahedron*, 2015, **71**, 5285–5295.
[18] Z. Cao, Y. Li, S. Wang, X. Guo, L. Wang and W. Zhao, *Synlett*, 2015, **26**, 921–926.
[19] J. M. Wood, D. P. Furkert and M. A. Brimble, *Org. Biomol. Chem.*, 2016, **14**, 7659–7664.

G. MATERIALS AND METHODS

Reagents were obtained from Aldrich Chemical (www.sigma-aldrich.com), Carbosynth (http://www.carbosynth.com), or Acros Organics (www.fishersci.com) and used without further purification. Solvents (Optima and HPLC grade) were obtained from Fisher Scientific (www.fishersci.com), degassed with Ar, and purified on a solvent drying system as described[20] unless otherwise indicated. Dimethyldioxirane (DMDO) was prepared as described[21] and the stock solution in acetone was stored over activated 4 Å molecular sieves at –80 °C.[22] All reactions were performed in flame-dried glassware under positive Ar pressure with magnetic stirring unless otherwise noted. Liquid reagents and solutions were transferred thru rubber septa via syringes flushed with Ar prior to use. Cold baths were generated as follows: 0 °C, wet ice/water; –78 °C, dry ice/acetone.

TLC was performed on 0.25 mm E. Merck silica gel 60 F254 plates and visualized under UV light (254 nm) or by staining with potassium permanganate ($KMnO_4$), or cerium ammonium molybdenate (CAM). Silica flash chromatography was performed on E. Merck 230–400 mesh silica gel 60.

Optical rotations were recorded on a JASCO model P-1020 digital polarimeter with PTC-103T temperature controller. IR spectra were recorded on a Bruker Optics Tensor 27 FTIR spectrometer using an attenuated total reflection (ATR) attachment with peaks reported in cm^{-1}. NMR spectra were recorded on a Bruker UltraShield Plus 500 MHz Avance III NMR or Bruker UltraShield Plus 600 MHz Avance III NMR with DCH CryoProbe at 24 °C in $CDCl_3$ unless otherwise indicated. Chemical shifts are expressed in ppm relative to TMS (^1H, 0 ppm) or solvent signals: $CDCl_3$ (^{13}C, 77.0 ppm), CD_3OD (^1H, 3.31 ppm; ^{13}C, 49.048 ppm), or acetone-d_6 (^{13}C, 206.2 ppm); coupling constants are expressed in Hz. NMR spectra were processed using Bruker TopSpin or MestReNova (http://mestrelab.com) software. Mass spectra were obtained at the MSKCC Analytical Core Facility on a Waters Acuity SQD LC-MS by electrospray (ESI) ionization or atmospheric pressure chemical ionization (AP-CI). High resolution mass spectra were obtained on a Waters Acuity Premiere XE TOF LC-MS by electrospray ionization.

N.B.: Atom numbers shown in chemical structures herein correspond to the standard carbohydrate numbering system used in the text of the article and Supporting Information and not to IUPAC nomenclature, which was used solely to name each compound. Compounds not cited in the paper are numbered herein from **S1**.

---

[20] A. B. G. Pangborn, M. A.; Grubbs, R. H.; Rosen, R. K.; Timmers, F. J., *Organometallics*, 1996, **15**, 1518–1520.
[21] W. C. Adam, Y.-Y.; Cremer, D.; Gauss, J.; Scheutzow, D.; Schindler, M., *J. Org. Chem.*, 1987, **52**, 2800–2803.
[22] S. J. D. Stachel, S. J., *Tetrahedron Lett.*, 2001, **42**, 6785–6787.

H. SYNTHESIS OF *C*1-MESYL-HYDROXYMETHYL-D-ARABINAL (14B)

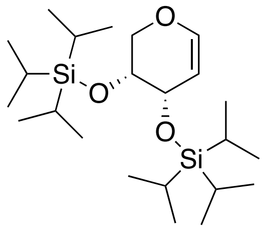

(+)-(((3*R*,4*S*)-3,4-dihydro-2*H*-pyran-3,4-diyl)bis(oxy))bis(triisopropylsilane) (TIPS-protected D-arabinal, 12).[23,24] In a 100 mL roundbottom flask, 3,4-di-*O*-acetyl-D-arabinal (2.0 g, 10 mmol) was dissolved in anhyd MeOH (0.4 M, 25 mL), followed by addition of sodium methoxide (25 wt% in MeOH, 5.0 mL, 22 mmol, 2.2 equiv). After 30 min at rt, Dowex 50W×2 (H^+) ion-exchange resin was added to neutralize the reaction mixture. The resin was then filtered over 1:1 silica/celite, and the filtrate was concentrated by rotary evaporation to afford the crude D-arabinal as a colorless oil in quantitative yield, which was used without further purification. **TLC**: R_f 0.15 (2:1 hexanes/EtOAc)

The crude D-arabinal above was dissolved in THF (0.2 M, 50 mL) and cooled to 0 °C. Sodium hydride (60 wt%, 1.60 g, 4.0 equiv) was added and stirred for 10 min, followed by dropwise addition of triisopropylsilyl trifluoromethanesulfonate (10.7 mL, 4.0 equiv) over 20 min at 0 °C. The cold bath was removed and the reaction was stirred at rt for 2 h. The reaction was re-cooled to 0 °C and quenched by dropwise addition of satd aq NH_4Cl, then warmed to rt. When bubbling had ceased, the reaction was diluted with Et_2O, and washed with brine. The organic layer was collected, dried over $MgSO_4$, filtered, and concentrated by rotary evaporation to afford the crude product. Purification by silica flash chromatography (hexanes + 0.5% Et_3N to remove triisopropylsilanol, then 19:1 hexanes/EtOAc) yielded TIPS-protected D-arabinal **12** (3.6 g, 83% over 2 steps) as a light yellow oil.

TLC: R_f 0.85 (19:1 hexanes/EtOAc). $[\alpha]_D^{19}$: +97.0° (*c* 1.0, $CDCl_3$). **IR** (ATR): 2894, 2811, 1617, 1401, 1369, 1226, 1102, 1040, 1015, 977, 910, 737. **^{1}H-NMR** (600 MHz): δ 6.28 (d, 1H, *J* = 5.9), 4.84 (t, 1H, *J* = 5.9), 4.29–4.20 (m, 1H), 4.06–4.01 (m, 1H), 3.99 (dt, 1H, *J* = 11.0, 3.0), 3.82–3.77 (m, 1H), 1.07 (s, 42H). **^{13}C-NMR** (151 MHz): δ 145.0, 102.2, 69.0, 64.6, 64.2, 18.1, 13.38. **ESI-MS** *m/z* (rel int): (pos) 451.1 ($[M+Na]^+$, 100). **HRMS** *m/z* calcd for $C_{23}H_{48}O_3Si_2Na$ ($[M+Na]^+$) 451.3040; found 451.3024.

---

[23] A. J. Janczuk, W. Zhang, P. R. Andreana, J. Warrick and P. G. Wang, *Carbohydr. Res.*, 2002, **337**, 1247–1259.

[24] H. Abe, S. Shut, S. Tamura and A. Matsuda, *Tetrahedron Lett.*, 2001, **42**, 6159–6161.

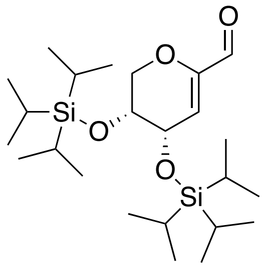

(+)-(3*R*,4*S*)-3,4-bis((triisopropylsilyl)oxy)-3,4-dihydro-2*H*-pyran-6-carbaldehyde (TIPS-protected *C*1-formyl-D-arabinal, S2).[25] In a 100 mL roundbottom flask, bis-*O*-TIPS-D-arabinal **12** (1.2 g, 2.9 mmol) was dissolved in THF (0.07 M, 40 mL) and cooled to –78 °C. A 1.7 M solution of *t*-BuLi in pentane (7.7 mL, 11.5 mmol, 4.0 equiv) was added slowly down the side of the cooled flask over 15 min, then warmed to 0 °C for 30 min. The reaction was re-cooled to –78 °C, and DMF was added dropwise over 10 min. The reaction was stirred at –78 °C for 10 h, then warmed to rt. The reaction was diluted with Et_2O and washed with satd aq NH_4Cl, $NaHCO_3$, water, and brine. The organic layer was collected, dried over $MgSO_4$, filtered, and concentrated by rotary evaporation to afford the crude product as a light yellow oil. Purification by silica flash chromatography (19:1 hexanes/EtOAc + 0.5% Et_3N) yielded TIPS-protected *C*1-formyl-D-arabinal **S2** (1.11 g, 87%) as a light yellow oil.

TLC: R_f 0.42 (9:1 hexanes/EtOAc). $[\alpha]_D^{20}$: +134° (*c* 1.0, $CDCl_3$). **IR** (ATR): 2953, 2871, 1706 (C=O), 1466, 1176, 1111, 1064, 1015, 908, 733. **^{1}H-NMR** (600 MHz): δ 9.20 (s, 1H), 5.81 (d, 1H, *J* = 5.0), 4.55–4.50 (m, 1H), 4.17 (t, 1H, *J* = 9.6), 4.10–3.99 (m, 2H), 1.14–1.02 (m, 42H). **^{13}C-NMR** (151 MHz): δ 186.8, 151.5, 120.3, 68.2, 65.9, 64.7, 18.0, 12.7. **ESI-MS** *m/z* (rel int): (pos) 479.4 ($[M+Na]^+$, 100); (neg) 491.4 ($[M+Cl]^-$, 45). **HRMS** *m/z* calcd for $C_{24}H_{48}O_4Si_2Na$ ($[M+Na]^+$) 479.2989; found 479.2975.

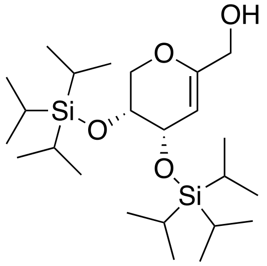

(+)-((3*R*,4*S*)-3,4-bis((triisopropylsilyl)oxy)-3,4-dihydro-2*H*-pyran-6-yl)methanol (*C*1-hydroxymethyl TIPS-protected D-arabinal, 13). In a 25 mL roundbottom flask, TIPS-protected *C*1-formyl-D-arabinal **S2** (535 mg, 1.2 mmol) was dissolved in 1:1 THF/MeOH (0.1 M, 12 mL) and cooled to 0 °C. Sodium borohydride (221 mg, 5.9 mmol, 5.0 equiv) was added in one portion. After 45 min at 0 °C, the cold bath was removed and the reaction was quenched by dropwise addition of satd aq NH_4Cl over 30 min as it warmed to rt. When bubbling had ceased, the reaction was diluted with Et_2O, and washed with water and brine. The organic layer was collected, dried over $MgSO_4$, filtered, and concentrated by rotary evaporation to afford the crude product. Purification by silica flash chromatography (19:1 hexanes/EtOAc + 0.5% Et_3N) yielded TIPS-protected *C*1-hydroxymethyl-D-arabinal **13** (510 mg, 95%) as a clear oil.

---

[25] J. Robertson, P. T. Chovatia, T. F. Fowler, J. M. Withey and D. J. Woollaston, *Org. Biomol. Chem.*, 2010, **8**, 226–233.

TLC: R_f 0.31 (9:1 hexanes/EtOAc). $[\alpha]_D^{19}$: +89.7° (*c* 1.0, $CDCl_3$). **IR** (ATR): 3385 (O–H st), 2946, 2869, 1721, 1464, 1387, 1247, 1151, 1091, 1015, 918, 888, 736, 679. **^{1}H-NMR** (600 MHz): δ 4.93 (d, 1H, *J* = 5.7), 4.32–4.24 (m, 1H), 4.09 (dd, 1H, *J* = 10.7, 9.6), 3.99 (dq, 3H, *J* = 7.7, 3.6), 3.86 (ddd, 1H, *J* = 9.6, 3.8, 1.5), 1.07 (m, 42H). **^{13}C-NMR** (151 MHz): δ 153.9, 98.7, 68.8, 65.0, 64.3, 62.7, 18.1, 12.9. **ESI-MS** *m/z* (rel int): (pos) 481.4 ($[M+Na]^+$, 100); (neg) 493.3 ($[M+Cl]^-$, 90). **HRMS** *m/z* calcd for $C_{24}H_{50}O_4Si_2Na$ ($[M+Na]^+$) 481.3145; found 481.3141.

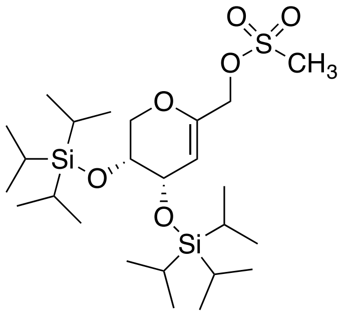

(+)-((3R,4S)-3,4-bis((triisopropylsilyl)oxy)-3,4-dihydro-2H-pyran-6-yl)methyl methanesulfonate (*C*1-mesyl-hydroxymethyl TIPS-protected D-arabinal, 14b). In a 25 mL roundbottom flask, TIPS-protected *C*1-hydroxymethyl-D-arabinal **13** (420 mg, 0.919 mmol) was dissolved in CH_2Cl_2 (0.1 M, 9.2 mL) and cooled to 0 °C. Triethylamine (0.167 mL, 1.2 mmol, 1.3 equiv) and methanesulfonyl chloride (0.100 mL, 1.3 mmol, 1.4 equiv) were added and stirred at 0 °C for 15 min. The cold bath was removed, and after 30 min at rt, the reaction was concentrated by rotary evaporation to afford the crude mesylate **14b** (581 mg, 87%), which was used without further purification.

TLC: R_f 0.22 (24:1 benzene/acetone). $[\alpha]_D^{19}$: +85.9° (*c* 1.0, $CDCl_3$). **IR** (ATR): 2946, 2869, 2357, 1674, 1464, 1362, 1243, 1174, 1072, 1019, 948, 886, 836, 737, 679. **^{1}H-NMR** (600 MHz): δ 5.09 (d, 1H, *J* = 5.5), 4.58 (d, 1H, *J* = 12.2), 4.54 (d, 1H, *J* = 12.2), 4.30 (dd, 1H, *J* = 5.5, 3.3), 4.09 (d, 1H, *J* = 10.1), 3.99, (dt, 1H, *J* = 10.4, 3.4), 3.90 (dt, 1H, *J* = 9.9, 2.3), 3.04 (s, 3H), 1.07 (m, 42H). **^{13}C-NMR** (151 MHz): δ 171.2, 148.3, 104.4, 69.0, 68.4, 65.4, 64.2, 38.2, 18.1, 12.8, 12.4. **ESI-MS** *m/z* (rel int): (pos) 559.4 ($[M+Na]^+$, 100); (neg) 571.4 ($[M+Cl]^-$, 35).

I. SYNTHESIS OF *C*1-PYRROLOMETHYL-D-ARABINAL SUBSTRATES (15, 16, 18)

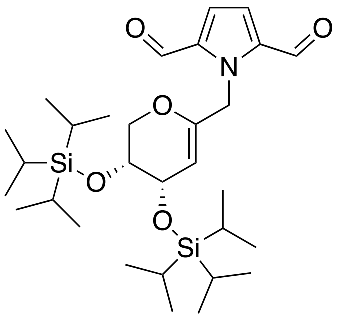

(+)-1-(((3*R*,4*S*)-3,4-bis((triisopropylsilyl)oxy)-3,4-dihydro-2*H*-pyran-6-yl)methyl)-1*H*-pyrrole-2,5-dicarbaldehyde (TIPS-protected pyrrole dicarboxaldehyde pyranoglycal, 15). In a 250 mL roundbottom flask, pyrrole-2,5-dicarboxaldehyde **8** (306 mg, 2.5 mmol, 2.0 equiv) and tetrabutylammonium iodide (460 mg, 1.24 mmol, 1.0 equiv) were dissolved in 60 mL THF. Sodium hydroxide (15 g, 373 mmol, 300 equiv) in 30 mL H_2O (12.4 M) was added. After 15 min at rt, crude mesylate **14b** (668 mg, 1.24 mmol, 1.0 equiv) in 5 mL THF was added dropwise over 10 min, then warmed to 60 °C for 12 h. Upon cooling to rt, the reaction was diluted with Et_2O, and washed with water and brine. The organic layer was collected, dried over $MgSO_4$,, filtered, and concentrated by rotary evaporation to afford the crude product. Purification by silica flash chromatography (19:1 hexanes/EtOAc + 0.5% Et_3N) yielded TIPS-protected pyrrole dicarboxaldehyde pyranoglycal **15** (498 mg, 71%) as a light yellow solid.

TLC: R_f 0.27 (9:1 hexanes/EtOAc). $[\alpha]_D^{20}$: +28.4° (*c* 0.5, $CDCl_3$). **IR** (ATR): 2943, 2867, 1673, 1463, 1246, 1102, 1068, 1017, 912, 884, 737, 682. **^{1}H-NMR** (600 MHz): δ 9.83 (s, 2H), 6.98 (s, 2H), 5.41 (d, 1H, *J* = 15.4), 5.35 (d, 1H, *J* = 15.4), 4.56 (d, 1H, *J* = 5.7), 4.19–4.14 (m, 1H), 3.96 (dd, 1H, *J* = 10.5, 9.6), 3.90 (dt, 1H, *J* = 10.6, 3.3), 3.79 (ddd, 1H, *J* = 9.5, 3.5, 1.4), 1.09–0.94 (m, 42H). **^{13}C-NMR** (151 MHz): δ 181.7, 151.4, 136.1, 121.2, 99.1, 68.6, 65.2, 64.2, 46.7, 18.2, 12.8, 12.4. **ESI-MS** *m*/*z* (rel int): (pos) 586.4 ($[M+Na]^+$, 100); (neg) 598.2 ($[M+Cl]^-$, 100). **HRMS** *m*/*z* calcd for $C_{30}H_{53}NO_5Si_2Na$ ($[M+Na]^+$) 586.3360; found 586.3344.

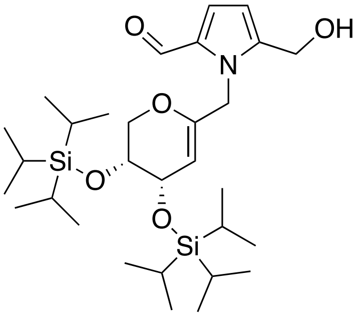

1-(((3*R*,4*S*)-3,4-bis((triisopropylsilyl)oxy)-3,4-dihydro-2*H*-pyran-6-yl)methyl)-5-(hydroxymethyl)-1*H*-pyrrole-2-carbaldehyde (TIPS-protected pyrrole monoalcohol pyranoglycal, 16).[26] In a 25 mL roundbottom flask, pyrrole dicarboxaldehyde pyranoglycal **15** (201 mg, 0.356 mmol) was dissolved in THF (0.03 M, 12 mL) and cooled to 0 °C, followed by

[26] Monoreduction of diformylpyrrole: (a) Y.-W. Chin, S. W. Lim, S.-H. Kim, D.-Y. Shin, Y.-G. Suh, Y.-B. Kim, Y. C. Kim and J. Kim, *BIoorg. Med. Chem. Lett.*, 2003, **13**, 79–81. (b) O. Tamura, N. Iyama and H. Ishibashi, *J. Org. Chem.*, 2004, **69**, 1475–1480. (c) R. Li, A. D. Lammer, G. M. Ferrence and T. D. Lash, *J. Org. Chem.*, 2014, **79**, 4078–4093.

addition of sodium borohydride (4.7 mg, 0.125 mmol, 0.35 equiv). The reaction was carefully monitored by TLC and once over-reduction was observed, the reaction was quenched with satd aq $NaHCO_3$, at 0 °C, despite the presence of minimal starting material. Upon warming to rt, the reaction mixture was diluted with Et_2O, and washed with water and brine. The organic layer was collected, dried over $MgSO_4$, filtered, and concentrated by rotary evaporation to afford the crude product. Purification by silica flash chromatography (9:1 hexanes/EtOAc + 0.5% Et_3N) yielded TIPS-protected pyrrole monoalcohol pyranoglycal **16** (147 mg, 73%) as a light yellow oil.

TLC: R_f 0.14 (9:1 hexanes/EtOAc). **IR** (ATR): 3399 (O–H st), 2947, 2867, 1663, 1463, 1159, 1062, 1019, 886, 737, 679. **^{1}H-NMR** (600 MHz): δ 9.50 (s, 1H), 6.89 (d, 1H, *J* = 4.0), 6.27 (d, 1H, *J* = 4.0), 5.13 (d, 1H, *J* = 15.7), 4.94 (d, 1H, *J* = 15.7), 4.79 (d, 1H, *J* = 5.5), 4.68–4.61 (m, 2H, 4.5), 4.26–4.20 (m, 1H), 4.02 (t, 1H, *J* = 10.0), 3.93 (dt, 1H, *J* = 10.1, 3.3), 3.84–3.80 (m, 1H), 1.12–0.98 (m, 42H). **^{13}C-NMR** (151 MHz): δ 179.4, 150.8, 142.1, 132.4, 110.8, 100.6, 68.4, 64.3, 56.1, 46.1, 18.2, 12.7, 12.4. **ESI-MS** *m/z* (rel int): (pos) 588.31 ([M+Na]$^+$, 100); (neg) 600.1 ([M+Cl]$^-$, 30). **HRMS** *m/z* calcd for $C_{30}H_{55}NO_5Si_2Na$ ([M+Na]$^+$) 588.3517; found 588.3495.

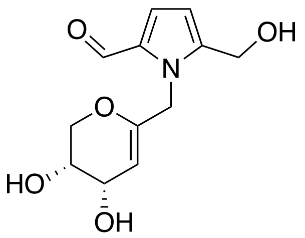

1-(((3*R*,4*S*)-3,4-dihydroxy-3,4-dihydro-2*H*-pyran-6-yl)methyl)-5-(hydroxymethyl)-1*H*-pyrrole-2-carbaldehyde (pyrrole monoalcohol pyranoglycal, 18). In a 15 mL roundbottom flask, TIPS-protected pyrrole monoalcohol pyranoglycal **16** (100 mg, 0.177 mmol) was dissolved in THF (0.03 M, 6 mL) and cooled to 0 °C. Tetrabutylammonium fluoride (1.0 M in THF, 406 μL, 2.3 equiv) was added and the reaction was stirred for 2 h. Concentration by rotary evaporation afforded the crude product. Purification by silica flash chromatography (99:1 EtOAc/MeOH + 1% Et_3N) yielded pyrrole monoalcohol pyranoglycal **18** (38 mg, 85%) as a light yellow oil.

TLC: R_f 0.12 (99:1 EtOAc/MeOH). **^{1}H-NMR** (600 MHz, CD_3OD): δ 9.43, (s, 1H), 7.00 (d, 1H, *J* = 4.0), 6.30 (d, 1H, *J* = 4.0), 5.10–5.00 (m, 2H), 4.64 (s, 2H), 4.39 (d, 1H, *J* = 4.8), 4.05–3.99 (m, 1H), 3.87 (ddd, 1H, *J* = 10.3, 3.5, 1.3), 3.84–3.79 (m, 1H), 3.75 (dt, 1H, *J* = 9.1, 3.8), 1.31 (t, 1H, *J* = 7.3), 1.24 (t, 1H, *J* = 7.1). **^{13}C-NMR** (151 MHz, CD_3OD): δ 181.1, 154.2, 145.2, 133.8, 126.1, 111.5, 98.6, 67.2, 66.7, 63.5, 56.6, 47.0. **ESI-MS** *m/z* (rel int): (pos) 276.1 ([M+Na]$^+$, 100); (neg) 288.1 ([M+Cl]$^-$, 100).

J. MERCURY-MEDIATED SPIROCYCLIZATIONS

GENERAL PROCEDURE FOR MERCURY-MEDIATED SPIROCYCLIZATIONS

In a 15 mL roundbottom flask, glycal substrate (1.0 equiv) was dissolved in 4 mL THF and the solution was cooled to –78 °C. Sodium hexamethyldisilazane (0.2 M in THF, 1.0 equiv) was added down the side of the flask over 10 min and the reaction was stirred for an additional 15 min. Mercuric acetate (1.2 equiv) was added and the reaction was warmed to 0 °C over 1 h, then to rt for the indicated times (**Table S1**). Sodium borohydride (1.2 equiv) was added and stirred for 2 min, followed by quenching slowly with satd aq $NaHCO_3$ to reduce the mercuric acetal. The crude mixture was diluted with EtOAc and washed with satd aq $NaHCO_3$ and brine. The organic layer was collected, dried over Na_2SO_4, filtered, and concentrated by rotary evaporation. The crude oil was filtered through a plug of silica gel with EtOAc to remove residual mercury, affording the crude product for NMR analysis.

Alternatively, the mercuric acetal was isolated by treatment with brine in lieu of sodium borohydride. The crude mixture was extracted with EtOAc, dried over Na_2SO_4, filtered, and concentrated by rotary evaporation. The crude oil was filtered through a plug of silica gel with EtOAc to remove residual mercury, affording the crude mercuric acetal for NMR analysis.

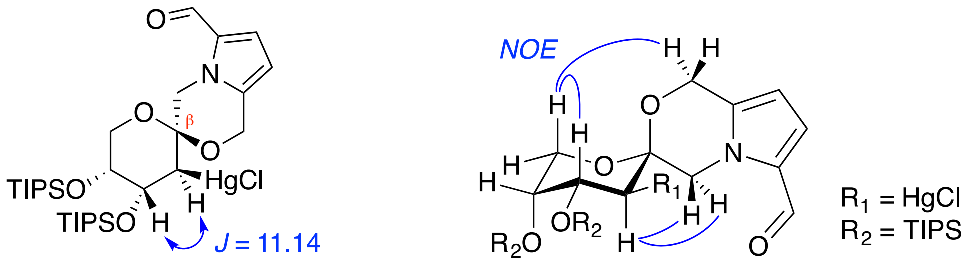

((2R,3S,4R,5R)-6'-formyl-4,5-bis((triisopropylsilyl)oxy)-3,4,5,6-tetrahydro-1'H,4'H-spiro[pyran-2,3'-pyrrolo[2,1-c][1,4]oxazin]-3-yl)mercury(II) chloride (TIPS-protected 2-mercurial spiroketal, 20b).

^{1}H-NMR (600 MHz): δ 9.47, (s, 1H), 6.94 (d, 1H, *J* = 4.1), 6.03 (d, 1H, *J* = 4.1), 4.81 (d, 1H, *J* = 15.2), 4.71 (d, 1H, *J* = 15.4), 4.41 (dd, 1H, *J* = 11.1, 2.3), 4.25–4.21 (ddd, 1H, *J* = 10.9, 5.7), 4.20 (m, 1H, *J* = 2.4), 4.17 (d, 1H, *J* = 14.2), 3.81 (dd, 1H, *J* = 12.1, 2.6), 3.70 (d, 1H, *J* = 12.1), 3.43 (d, 1H, *J* = 11.1), 1.45–0.81 (m, 42H). **^{13}C-NMR** (151 MHz): δ 178.9, 134.1, 131.1, 124.0, 105.0, 98.1, 72.0, 71.6, 68.1, 66.2, 60.1, 58.0, 18.6, 13.2. **ESI-MS** *m/z* (rel int): (pos) 824.2 ($[M+Na]^+$, 40); (neg) 836.3 ($[M+Cl]^-$, 100).

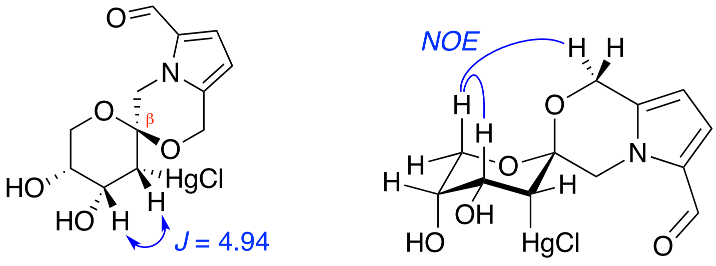

((2R,3R,4R,5R)-6'-formyl-4,5-dihydroxy-3,4,5,6-tetrahydro-1'H,4'H-spiro[pyran-2,3'-pyrrolo[2,1-c][1,4]oxazin]-3-yl)mercury(II) chloride (2-mercurial spiroketal, 22b).

^{1}**H-NMR** (600 MHz, CD_3OD): δ 9.36, (s, 1H), 7.03 (d, 1H, *J* = 4.0), 6.08 (d, 1H, *J* = 4.2), 4.86 (d, 1H), 4.72 (d, 1H, *J* = 15.7), 4.69 (dd, 1H, *J* = 5.0, 3.2), 4.65 (d, 1H, *J* = 13.7), 4.04 (d, 1H, *J* = 13.8), 3.91–3.87 (m, 1H), 3.80 (dd, 1H, *J* = 12.3, 2.0), 2.72 (d, 1H, *J* = 5.0), 2.19 (t, 1H, *J* = 7.6). ^{13}**C-NMR** (151 MHz, CD_3OD): δ 180.3, 173.0, 161.5, 125.7, 106.1, 99.1, 68.3, 66.8, 59.4, 54.7, 54.3, 36.6. **ESI-MS** *m/z* (rel int): (pos) 512.0 ($[M+Na]^+$, 20); (neg) 524.1 ($[M+Cl]^-$, 100), 488.2 ($[M+H]^-$, 30).

K. SYNTHESIS OF SHENSONGINE A (2)

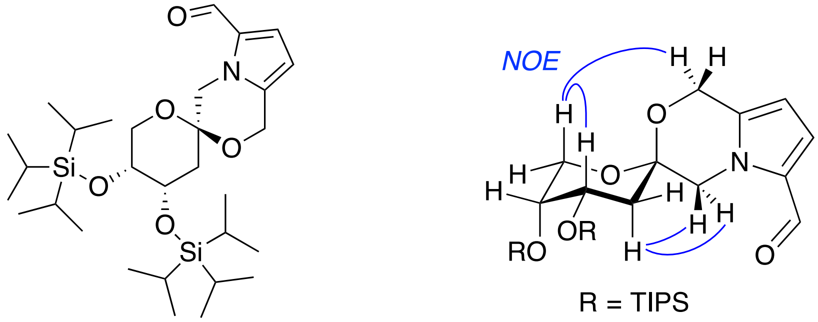

(+)-(2*S*,4*S*,5*R*)-4,5-bis((triisopropylsilyl)oxy)-3,4,5,6-tetrahydro-1'*H*,4'*H*-spiro[pyran-2,3'-pyrrolo[2,1-*c*][1,4]oxazine]-6'-carbaldehyde (TIPS-protected β-spiroketal, 17). In a 10 mL roundbottom flask, TIPS-protected pyrrole monoalcohol pyranoglycal **16** (50 mg, 0.088 mmol) was dissolved in 4.4 mL CH_2Cl_2 (0.02 M) and cooled to 0 °C. Dichloroacetic acid (0.02 M solution in CH_2Cl_2, 0.442 mL, 0.10 equiv) was added dropwise and stirred for 30 min. Upon warming to rt, the reaction was diluted with EtOAc, and washed with satd aq $NaHCO_3$, water and brine. The organic layer was collected, dried over $MgSO_4$, filtered, and concentrated by rotary evaporation to afford the crude product as a single diastereomer. Purification by silica flash chromatography (9:1 hexanes/EtOAc + 0.5% Et_3N) yielded TIPS-protected β-spiroketal **17** (49 mg, 98%) as a light yellow oil.

Alternatively, treatment of pyranoglycal **16** with *p*-toluenesulfonic acid (0.10 equiv) for 30 min also provided efficient access to TIPS-protected β-spiroketal **17** as a single diastereomer.

TLC: R_f 0.21 (9:1 hexanes/EtOAc). $[\alpha]_D^{19}$: +73.2° (*c* 1.0, $CDCl_3$). **IR** (ATR): 2948, 2867, 1661, 1464, 1403, 1378, 1321, 1255, 1131, 1054, 918, 883, 737, 681. **^{1}H-NMR** (600 MHz): δ 9.45 (s, 1H), 6.90 (d, 1H, *J* = 4.1), 5.98 (d, 1H, *J* = 4.1), 4.76 (d, 1H, *J* = 15.3), 4.71 (d, 1H, *J* = 15.4), 4.54 (d, 1H, *J* = 14.1), 4.24 (ddd, 1H, *J* = 11.4, 4.5, 2.5), 4.03 (d, 1H, *J* = 13.8), 4.03 (m, 1H), 3.73 (dd, 1H, *J* = 11.9, 2.4), 3.67 (d, 1H, *J* = 11.8), 2.29 (t, 1H, *J* = 11.9), 1.80 (dd, 1H, *J* = 12.5, 4.5), 1.08 (m, 42H). **^{13}C-NMR** (151 MHz): δ 178.6, 134.8, 131.1, 124.0, 104.6, 95.7, 69.8, 67.4, 66.6, 57.4, 52.7, 36.2, 18.2. **ESI-MS** *m/z* (rel int): (pos) 588.3 ($[M+Na]^+$, 100); (neg) 600 ($[M+Cl]^-$, 100). **HRMS** *m/z* calcd for $C_{30}H_{55}NO_5Si_2Na$ ($[M+Na]^+$) 588.3517; found 588.3519.

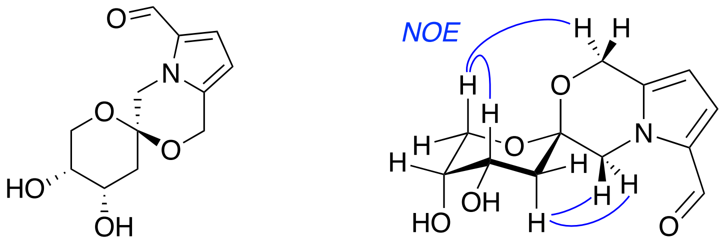

(–)-(2*S*,4*S*,5*R*)-4,5-dihydroxy-3,4,5,6-tetrahydro-1'*H*,4'*H*-spiro[pyran-2,3'-pyrrolo[2,1-*c*][1,4]oxazine]-6'-carbaldehyde (shensongine A, 2). In a 10 mL roundbottom flask, TIPS-protected β-spiroketal **17** (25 mg, 0.044 mmol) was dissolved in 4.4 mL THF (0.01 M) and cooled to 0 °C. Tetrabutylammonium fluoride (1.0 M, 0.097 mmol, 2.2 equiv) was added and the reaction was stirred for 1 h. Concentration by rotary evaporation afforded the crude product.

Purification by silica flash chromatography (99:1 EtOAc/MeOH + 1% Et_3N) yielded shensongine A **2** (10.2 mg, 91%) as a white solid.

TLC: R_f 0.23 (98:2 EtOAc/MeOH). $[\alpha]_D^{20}$: –38.8° (*c* 0.9, MeOH). **IR** (ATR): 3345 (O–H st), 2962, 2930, 1645, 1499, 1446, 1409, 1320, 1187, 1046, 972, 846, 774, 728, 612. **^{1}H-NMR** (600 MHz, acetone-*d*6): δ 9.46 (s, 1H), 6.97 (d, 1H, *J* = 4.1), 6.05 (d, 1H, *J* = 4.0), 4.85 (d, 1H, *J* = 15.6), 4.74 (d, 1H, *J* = 15.7), 4.55 (d, 1H, *J* = 14.0), 4.07 (m, 1H), 3.95 (d, 1H, *J* = 14.0), 3.84 (m, 1H), 3.79 (dd, 1H, *J* = 11.4), 3.75 (dd, 1H, *J* = 12.3, 2.2), 1.99 (dd, 1H, 12.8, 11.4), 1.91 (dd, 1H, 12.8, 5.2). **^{13}C-NMR** (151 MHz, acetone-*d*6): δ 179.1, 135.4, 132.1, 124.2, 105.3, 96.2, 68.3, 65.9, 65.5, 58.1, 53.1, 36.3. **ESI-MS** *m/z* (rel int): (pos) 276.0 ($[M+Na]^+$, 80). **HRMS** *m/z* calcd for $C_{12}H_{15}NO_5Na$ ($[M+Na]^+$) 276.0848; found 276.0858.

^{1}H-NMR (600 MHz, CD_3OD): δ 9.36 (s, 1H), 7.02 (d, 1H, *J* = 4.1), 6.07 (d, 1H, *J* = 4.1), 4.84 (d, 1H, *J* = 15.7), 4.74 (d, 1H, *J* = 15.8), 4.60 (d, 1H, *J* = 13.9), 4.07 (ddd, 1H, *J* = 11.7, 5.2, 3.0), 3.97 (d, 1H, *J* = 13.9), 3.81 (dd, 1H, *J* = 12.0, 1.1), 3.78 (m, 1H, *J* = 2.6), 3.76 (dd, 1H, *J* = 12.0, 2.1), 1.99 (dd, 1H, *J* = 12.7, 11.7), 1.90 (dd, 1H, *J* = 12.6, 5.0). **^{13}C-NMR** (151 MHz, CD_3OD): δ 180.2, 137.1, 132.4, 125.9, 106.1, 96.7, 68.7, 66.3, 66.1, 58.6, 53.5, 36.1.

^{1}H-NMR (600 MHz, $CDCl_3$): δ 9.45 (s, 1H), 6.92 (d, 1H, *J* = 4.1), 6.01 (d, 1H, *J* = 4.1), 4.82 (d, 1H, *J* = 15.4), 4.74 (d, 1H, *J* = 15.5), 4.69 (d, 1H, *J* = 14.1), 4.17 (m, 1H), 4.02 (d, 1H, *J* = 14.1), 3.89 (m, 1H), 3.89 (dd, 1H), 3.81 (dd, 1H, *J* = 12.7, 1.3), 2.05 (dd, 1H, *J* = 13.0, 5.4), 1.92 (dd, 1H, *J* = 13.0, 11.5). **^{13}C-NMR** (151 MHz, $CDCl_3$): δ 178.8, 134.1, 131.1, 124.0, 104.8, 95.4, 67.3, 65.0, 64.5, 57.7, 52.2, 35.7.

L. Synthesis of pollenopyrroside A (1)

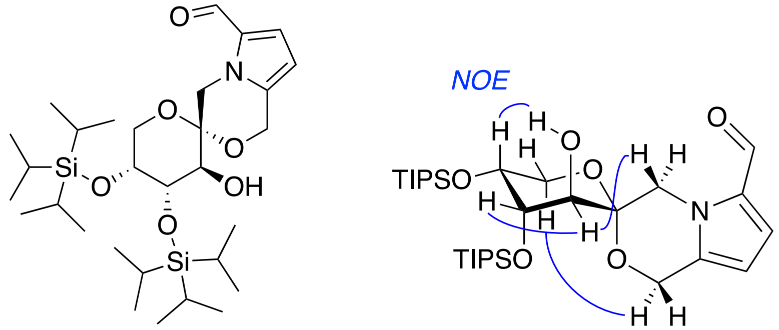

(+)-(2*S*,3*S*,4*S*,5*R*)-3-hydroxy-4,5-bis((triisopropylsilyl)oxy)-3,4,5,6-tetrahydro-1'*H*,4'*H*-spiro[pyran-2,3'-pyrrolo[2,1-*c*][1,4]oxazine]-6'-carbaldehyde (TIPS-protected 2-OH-α-spiroketal, 24).[27] In a 5 mL roundbottom flask, TIPS-protected pyrrole monoalcohol pyranoglycal **16** (9.4 mg, 0.017 mmol) was dissolved in 2 mL MeOH/CH_2Cl_2 (final ratio 5:1:1 MeOH/CH_2Cl_2/acetone) and cooled to –78 °C. DMDO (0.277 mL, 0.090 M in acetone, 1.5 equiv) was added and stirred for 20 min, then warmed to 0 °C for 1 h. After warming to rt, the reaction mixture was extracted with EtOAc, washed with water, brine, dried over Na_2SO_4, filtered, and concentrated by rotary evaporation to provide the crude inversion α-spiroketal as a single diastereomer. Purification by silica flash chromatography with 19:1 hexanes/EtOAc + 1% Et_3N yielded the desired 2-hydroxy-α-spiroketal **24** (8.1 mg, 84%) as a white solid.

TLC: R_f 0.19 (9:1 hexanes/EtOAc). $[\alpha]_D^{19}$: +79.4° (*c* 1.0, $CDCl_3$). **IR** (ATR): 3310 (O–H st), 2939, 2864, 1635, 1461, 1316, 1255, 1154, 1042, 947, 884, 830, 775, 678. **^{1}H-NMR** (600 MHz): δ 9.48 (s, 1H), 6.91 (d, 1H, *J* = 4.1), 6.01 (d, 1H, *J* = 4.1), 4.87 (d, 1H, *J* = 15.2), 4.74 (d, 1H, *J* = 15.1), 4.63 (d, 1H, *J* = 14.0), 4.40 (d, 1H, *J* = 14.0), 4.20 (dt, 1H, *J* = 3.4, 1.6), 3.99 (d, 1H, *J* = 9.1), 3.89 (ddd, 1H, 10.4, 9.2, 3.5), 3.77 (dd, 1H, *J* = 12.6, 1.2), 3.74 (dd, 1H, *J* = 12.6, 1.8), 2.33 (d, 1H, *J* = 10.5), 1.23–1.01 (m, 42H). **^{13}C-NMR** (151 MHz): δ 178.7, 134.3, 131.4, 123.6, 104.6, 97.2, 73.2, 71.4, 70.9, 64.1, 57.4, 49.1, 18.4, 13.1. **ESI-MS** *m/z* (rel int): (pos) 604.3 ($[M+Na]^+$, 100); (neg) 580.1 ($[M–H]^-$, 100). **HRMS** *m/z* calcd for $C_{30}H_{55}NO_6Si_2Na$ ($[M+Na]^+$) 604.3466; found 604.3450.

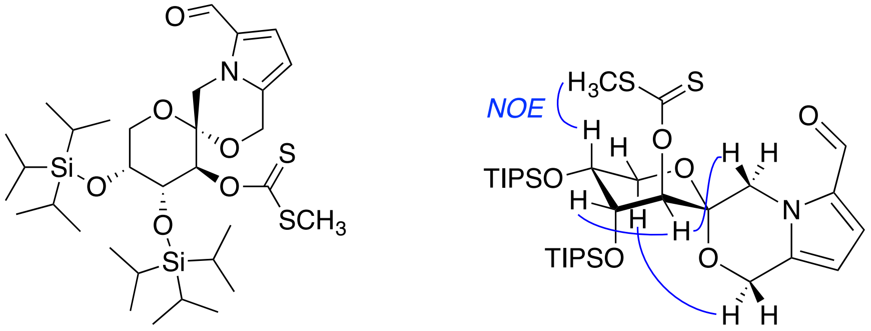

(+)-*O*-((2*S*,3*S*,4*S*,5*R*)-6'-formyl-4,5-bis((triisopropylsilyl)oxy)-3,4,5,6-tetrahydro-1'*H*,4'*H*-spiro[pyran-2,3'-pyrrolo[2,1-*c*][1,4]oxazin]-3-yl) *S*-methyl carbonodithioate (TIPS-protected 2-xanthate-α-spiroketal, 26). In a 10 mL roundbottom flask, TIPS-protected 2-hydroxy-α-spiroketal **24** (48 mg, 0.082 mmol) was dissolved in 4 mL toluene (0.02 M) and

---

[27] J. S. Potuzak, S. B. Moilanen and D. S. Tan, *J. Am. Chem. Soc.*, 2005, **127**, 13796–13797.

cooled to 0 °C. Sodium hydride (13 mg, 60% w 0.33 mmol 4.0 equiv) was added and stirred for 15 min. Dimethyl carbonotrithioate (0.045 mL, 0.41 mmol, 5.0 equiv) was added and warmed to 80 °C for 2 h. After cooling to rt, the reaction was re-cooled to 0 °C and slowly quenched by dropwise addition of satd aq NH_4Cl. The reaction mixture was extracted with EtOAc, washed with satd aq $NaHCO_3$, water, brine, dried over Na_2SO_4, filtered, and concentrated by rotary evaporation. The crude product was filtered over a short column of silica gel with 19:1 hexanes/EtOAc + 0.5% Et_3N providing the desired 2-xanthate **26** (45 mg, 82%) as a light yellow solid, which was taken forward to the following deoxygenation step without further purification.

TLC: R_f 0.31 (9:1 hexanes/EtOAc). $[\alpha]_D^{18}$: +57.9° (*c* 1.0, $CDCl_3$). **IR** (ATR): 2934, 2866, 1661, 1464, 1411, 1376, 1318, 1203, 1148, 1074, 914, 736, 685. **^{1}H-NMR** (600 MHz): δ 9.43 (s, 1H), 6.87 (d, 1H, *J* = 4.1), 6.24 (d, 1H, *J* = 3.0), 5.97 (d, 1H, *J* = 4.0), 4.97 (d, 1H, *J* = 15.4), 4.89 (d, 1H, *J* = 15.4), 4.72 (dd, 1H, *J* = 6.3, 3.0), 4.58 (d, 1H, *J* = 14.1), 4.17 (d, 1H, *J* = 14.1), 4.07 (dt, 1H, *J* = 6.5, 3.6), 3.95 (dd, 1H, *J* = 11.5, 3.0), 3.88 (dd, 1H, *J* = 11.5, 4.1), 2.62 (s, 3H), 1.03 (m, 42H). **^{13}C-NMR** (151 MHz): δ 215.5, 178.5, 138.0, 135.0, 131.1, 123.7, 104.6, 102.6, 92.2, 84.7, 76.5, 61.7, 58.0, 48.1, 19.6, 17.9, 11.9. **HRMS** *m/z* calcd for $C_{32}H_{57}NO_6S_2Si_2Na$ ($[M+Na]^+$) 694.3064; found 694.3069.

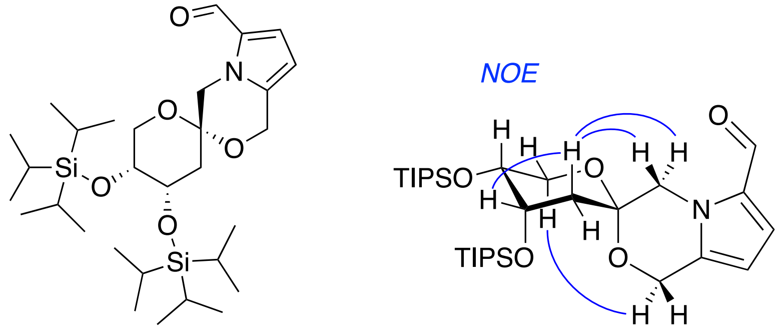

(+)-(2R,4S,5R)-4,5-bis((triisopropylsilyl)oxy)-3,4,5,6-tetrahydro-1'H,4'H-spiro[pyran-2,3'-pyrrolo[2,1-c][1,4]oxazine]-6'-carbaldehyde (TIPS-protected α-spiroketal, 23).[28] In a 10 mL roundbottom flask, 2-xanthate-α-spiroketal **26** (12.7 mg, 0.019 mmol) was dissolved in 4 mL toluene. Azobisisobutyronitrile (6.5 mg, 0.040 mmol, 2.1 equiv) and tributylstannane (0.011 mL, 0.040 mmol, 2.1 equiv) were added and the reaction was warmed to 85 °C and stirred for 3 h. After warming to rt, the reaction mixture was extracted with EtOAc, washed with water, brine, dried over Na_2SO_4, filtered, and concentrated by rotary evaporation. Purification by silica flash chromatography (19:1 EtOAc/MeOH + 1% Et_3N) yielded TIPS-protected α-spiroketal **23** (7.6 mg, 71%) as a colorless oil.

TLC: R_f 0.26 (9:1 hexanes/EtOAc). $[\alpha]_D^{19}$: +116.5° (*c* 1.0, $CDCl_3$). **IR** (ATR): 2948, 2867, 1662, 1464, 1404, 1378, 1322, 1255, 1131, 1053, 921, 883, 744, 681. **^{1}H-NMR** (600 MHz): δ 9.45 (s, 1H), 6.90 (d, 1H, *J* = 4.1 Hz), 5.98 (d, 1H, *J* = 4.1 Hz), 4.77 (d, 1H, *J* = 15.4 Hz), 4.72 (d, 1H, *J* = 15.4 Hz), 4.59 (d, 1H, *J* = 14.1 Hz), 4.12 (ddd, 1H, *J* = 11.3, 4.7, 2.7 Hz), 4.00 (d, 1H, *J* = 14.1 Hz), 3.76 (m, 1H, *J* = 2.4), 3.69 (dd, 1H, *J* = 11.8, 1.1), 3.66 (dd, 1H, *J* = 11.8, 2.3), 2.16 (t, 1H, *J* = 11.8), 1.71 (dd, 1H, *J* = 12.6, 4.6 Hz), 0.91 (m, 42H). **^{13}C-NMR** (151 MHz): δ 178.8, 134.8, 131.3, 124.1, 104.8, 95.8, 69.4, 67.0, 66.6, 57.6, 52.7, 36.2, 26.1, 18.4. **ESI-MS** *m/z* (rel int): (pos) 588.4 ($[M+Na]^+$, 100); (neg) 600.3 ($[M+Cl]^-$, 100). **HRMS** *m/z* calcd for $C_{30}H_{55}NO_5Si_2Na$ ($[M+Na]^+$) 588.3517; found 588.3511.

---

[28] S. Iacono and J. R. Rasmussen, *Org. Synth.*, 1986, **64**, 57–62.

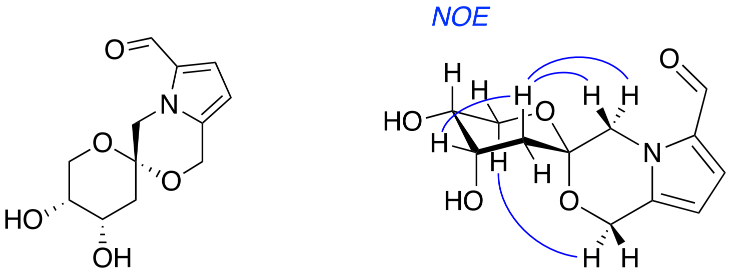

(+)-(2R,4S,5R)-4,5-dihydroxy-3,4,5,6-tetrahydro-1'H,4'H-spiro[pyran-2,3'-pyrrolo[2,1-c][1,4]oxazine]-6'-carbaldehyde (pollenopyrroside A, 1). In a 5 mL roundbottom flask, TIPS-protected α-spiroketal **23** (10 mg, 0.018 mmol) was dissolved in 1.8 mL THF and cooled to 0 °C. Tetrabutylammonium fluoride (1.0 M, 0.039 mmol, 2.2 equiv) was added and the reaction was stirred for 1 h. Concentration by rotary evaporation afforded the crude product. Purification by silica flash chromatography (99:1 EtOAc/MeOH + 1% Et_3N) yielded pollenopyrroside A **1** (4.2 mg, 95%) as a light yellow solid.

TLC: R_f 0.26 (98:2 EtOAc/MeOH). $[\alpha]_D^{19}$: +128.7° (*c* 0.08, MeOH). **IR** (ATR): 3344 (O–H st), 2928, 1642, 1446, 1405, 1372, 1318, 1192, 1145, 967, 877, 770, 720, 663, 615. **^{1}H-NMR** (600 MHz, acetone-*d*6): δ 9.47 (s, 1H), 6.98 (d, 1H, *J* = 4.1), 6.06 (d, 1H, *J* = 4.1), 4.93 (d, 1H, *J* = 15.6), 4.85 (d, 1H, *J* = 15.6), 4.46 (d, 1H, *J* = 14.2), 4.02 (m, 1H), 4.00 (d, 1H, *J* = 14.2), 3.75 (dd, 1H, *J* = 10.5), 3.71–3.66 (m, 1H), 3.56–3.51 (m, 1H), 2.24 (dd, 14.5, 3.5), 2.09 (dd, 14.5, 3.5). **^{13}C-NMR** (151 MHz, acetone-*d*6): δ 179.1, 135.3, 132.1, 124.1, 105.3, 95.1, 67.6, 66.8, 60.9, 58.1, 52.3, 38.4. **ESI-MS** *m*/*z* (rel int): (pos) 276.0 ($[M+Na]^+$, 90). **HRMS** *m*/*z* calcd for $C_{12}H_{15}NO_5Na$ ($[M+Na]^+$) 276.0848; found 276.0835.

M. Synthesis of 2-hydroxy analogues (25, 28)

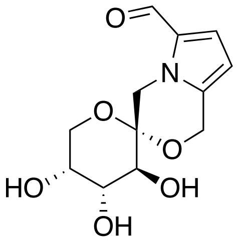
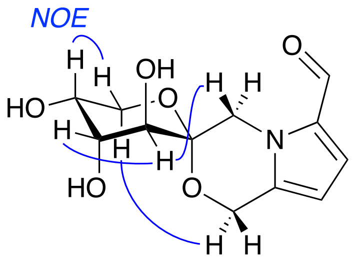

(2S,3S,4R,5R)-3,4,5-trihydroxy-3,4,5,6-tetrahydro-1'H,4'H-spiro[pyran-2,3'-pyrrolo[2,1-c][1,4]oxazine]-6'-carbaldehyde (2-OH pollenopyrroside A, 25). In a 5 mL roundbottom flask, TIPS-protected 2-hydroxy-α-spiroketal **24** (8.4 mg, 0.014 mmol) was dissolved in 1.4 mL THF (0.01 M) and cooled to 0 °C. Tetrabutylammonium fluoride (1.0 M, 0.032 mmol, 2.2 equiv) was added and the reaction was stirred for 1 h. Concentration by rotary evaporation afforded the crude product. Purification by silica flash chromatography (97:3 EtOAc/MeOH + 1% Et_3N) yielded 2-OH pollenopyrroside A **25** (3.7 mg, 95%) as a light yellow solid.

TLC: R_f 0.12 (95:5 EtOAc/MeOH). $[\alpha]_D^{19}$: +33.4° (*c* 1.0, acetone-d_6). **IR** (ATR): 3361 (O–H st), 2925, 2859, 1698, 1647, 1460, 1411, 1320, 1257, 1185, 1071, 1033, 928, 807. **^{1}H-NMR** (600 MHz, acetone-*d*6): δ 9.48 (s, 1H), 6.99 (d, 1H, *J* = 4.1), 6.06 (d, 1H, *J* = 4.1), 4.96 (d, 1H, *J* = 15.5, 1.2), 4.84 (d, 1H, *J* = 15.4), 4.56 (dd, 1H, *J* = 14.6, 1.0), 4.28 (d, 1H, *J* = 14.6), 4.08 (d, 1H, *J* = 2.4), 4.07–4.05 (m, 1H), 4.03 (ddd, 1H, *J* = 4.7, 2.1), 3.77 (dd, 1H, *J* = 12.0, 3.0), 3.68 (dd, 1H, *J* = 12.0, 4.3). **^{13}C-NMR** (151 MHz, acetone-*d*6): δ 179.0, 136.0, 132.4, 124.2, 105.2, 104.8, 86.5, 83.9, 80.0, 62.5, 58.0, 49.5. **ESI-MS** *m/z* (rel int): (neg) 268.1 ($[M–H]^+$, 100). **HRMS** *m/z* calcd for $C_{12}H_{14}NO_6$ ($[M–H]^-$) 268.0821; found 268.0829.

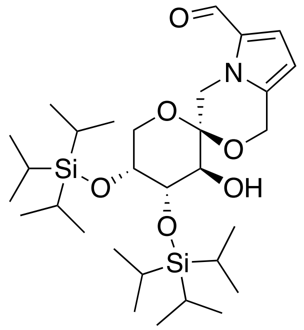
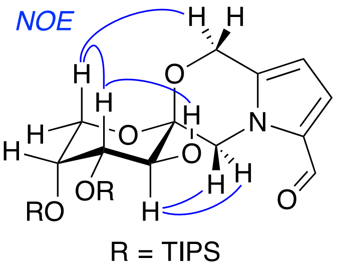

(2*R*,3*S*,4*S*,5*R*)-3-hydroxy-4,5-bis((triisopropylsilyl)oxy)-3,4,5,6-tetrahydro-1'*H*,4'*H*-spiro[pyran-2,3'-pyrrolo[2,1-*c*][1,4]oxazine]-6'-carbaldehyde (TIPS-protected 2-OH-β-spiroketal, 27).[29] In a 5 mL roundbottom flask, TIPS-protected pyrrole monoalcohol pyranoglycal **16** (18 mg, 0.032 mmol) was dissolved in 1 mL CH_2Cl_2 (final ratio 2:1 CH_2Cl_2/acetone) and cooled to –78 °C. DMDO (0.530 mL, 0.090 M in acetone, 1.5 equiv) was added and stirred for 10 min, then warmed to 0 °C. After 30 min, Ti(O-*i*Pr)$_4$ (0.020 mL, 0.064 mmol, 2.0 equiv) was added and the reaction mixture was warmed to rt. After 1 h, the reaction was quenched with satd aq $NaHCO_3$. The reaction mixture was extracted with EtOAc, washed with satd aq NH_4Cl, water, brine, dried over Na_2SO_4, filtered, and concentrated by rotary evaporation to provide the crude retention β-spiroketal as a single diastereomer. Purification by

---

[29] S. B. Moilanen, J. S. Potuzak and D. S. Tan, *J. Am. Chem. Soc.*, 2006, **128**, 1792–1793.

silica flash chromatography with 19:1 hexanes/EtOAc + 1% Et_3N yielded the desired 2-hydroxy-β-spiroketal **27** (14.4 mg, 78%) as a colorless oil.

TLC: R_f 0.34 (9:1 hexanes/EtOAc). **IR** (ATR): 3399 (O–H st), 2946, 2867, 1655, 1464, 1397, 1323, 1252, 1059, 915, 778, 736, 681. **^{1}H-NMR** (600 MHz): δ 9.47 (s, 1H), 6.90 (d, 1H, *J* = 4.1 Hz), 6.00 (d, 1H, *J* = 4.1 Hz), 4.89 (d, 1H, *J* = 15.1 Hz), 4.80 (d, 1H, *J* = 15.1 Hz), 4.66 (d, 1H, *J* = 14.1 Hz), 4.47 (d, 1H, *J* = 14.1 Hz), 4.19 (dt, 1H, *J* = 3.1, 1.7), 4.13 (dd, 1H *J* = 9.4, 2.7 Hz), 3.99 (dd, 1H, *J* = 9.4, 7.3 Hz), 3.71 (d, 2H, *J* = 1.6 Hz), 2.04 (d, 1H, *J* = 7.3 Hz), 1.20–1.04 (m, 42H). **^{13}C-NMR** (151 MHz): δ 178.7, 133.9, 131.5, 123.8, 104.7, 96.9, 72.6, 72.1, 70.7, 65.7, 57.6, 49.1, 18.3, 12.9. **ESI-MS** *m/z* (rel int): (pos) 604.4 ([M+Na]$^+$, 100); (neg) 580.2 ([M–H]$^-$, 100), 616.1 ([M+Cl]$^-$, 40). **HRMS** *m/z* calcd for $C_{30}H_{55}NO_6Si_2Na$ ([M+Na]$^+$) 604.3466; found 604.3491.

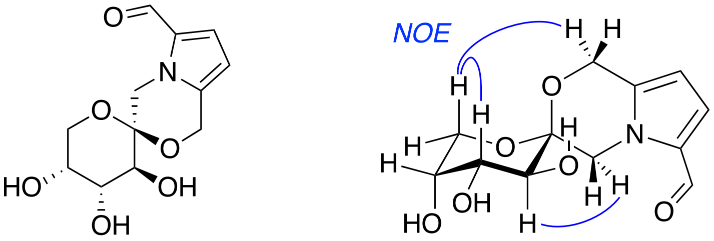

(2*R*,3*S*,4*R*,5*R*)-3,4,5-trihydroxy-3,4,5,6-tetrahydro-1'*H*,4'*H*-spiro[pyran-2,3'-pyrrolo[2,1-*c*][1,4]oxazine]-6'-carbaldehyde (2-OH β-spiroketal, 28). In a 5 mL roundbottom flask, TIPS-protected 2-hydroxy-β-spiroketal **27** (6.9 mg, 0.012 mmol) was dissolved in 1.2 mL THF (0.01 M) and cooled to 0 °C. Tetrabutylammonium fluoride (1.0 M, 0.026 mmol, 2.2 equiv) was added and the reaction was stirred for 1 h. Concentration by rotary evaporation afforded the crude product. Purification by silica flash chromatography (97:3 EtOAc/MeOH + 1% Et_3N) yielded 2-hydroxy-β-spiroketal **28** (2.9 mg, 92%) as a light yellow solid.

TLC: R_f 0.11 (95:5 EtOAc/MeOH). $[\alpha]_D^{19}$: –27.7° (*c* 1.0, acetone-d_6). **IR** (ATR): 3397 (O–H st), 2926, 1698, 1647, 1463, 1408, 1324, 1408, 1324, 1255, 1179, 1036, 923, 873, 795. **^{1}H-NMR** (600 MHz, acetone-*d*6): δ 9.48 (s, 1H), 6.99 (d, 1H, *J* = 4.0), 6.05 (d, 1H, *J* = 4.0), 5.10 (d, 1H, *J* = 15.4), 4.85 (d, 1H, *J* = 15.4), 4.50 (d, 1H, *J* = 14.0), 4.28 (d, 1H, *J* = 14.0), 4.18 (t, 1H, *J* = 7.6), 3.97 (d, 1H, *J* = 7.9), 3.90 (td, 1H, *J* = 6.8, 3.4), 3.71 (dd, 1H, *J* = 11.9, 3.4), 3.63 (dd, 1H, *J* = 11.9, 6.5). **^{13}C-NMR** (151 MHz, acetone-*d*6): δ 179.1, 135.8, 132.4, 124.2, 105.3, 100.1, 84.2, 81.1, 75.9, 64.3, 58.0, 50.4. **ESI-MS** *m/z* (rel int): (neg) 268.1 ([M–H]$^-$, 30). **HRMS** *m/z* calcd for $C_{12}H_{14}NO_6$ ([M–H]$^-$) 268.0821; found 268.0820.

N. Comparison of synthetic and authentic natural products (1, 2)

Comparative analytical data is provided for synthetic pollenopyrroside A (**1**) and shensongine A (**2**, xylapyrroside A, *ent*-capparisine B) prepared herein and analyzed by 600 MHz NMR, and original data reported for pollenopyrroside A and capparisine B using 500 MHz NMR, and shensongine A and xylapyrroside A using 400 MHz NMR. Peak assignments are based on the standard carbohydrate numbering system used in the manuscript and Supporting Information herein, and are based on COSY, NOESY, HSQC, and HMBC multidimensional NMR analysis.

1. Pollenopyrroside A (1)

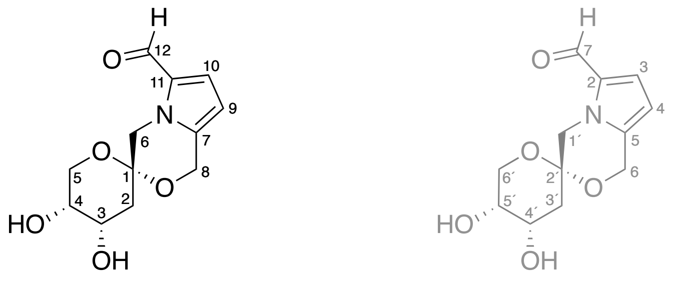

1
carbohydrate numbering herein

pollenopyrroside A
original numbering (*Zhang,* et al.)

Table S5. Comparative ^{1}H- and ^{13}C-NMR data for pollenopyrroside A in acetone-d_6.

	synthetic sample (1)[b]		literature[c] (Zhang, *et al.*)[11]		
position[a]	δ_H (*J* values in Hz)	δ_C	δ_H (*J* values in Hz)	δ_C	lit. position[d]
1		95.1		95.0	2´
2	2.09 (dd, 14.5, 3.5)	38.4	2.09 (dd, 14.5, 3.5)	38.4	3´
	2.24 (dd, 14.5, 3.5)		2.24 (dd, 14.5, 3.5)		
3	4.02 (m)	67.6	4.05 (m)	67.6	4´
4	3.71–3.66 (m)	66.8	3.71 (m)	66.9	5´
5	3.56–3.51 (m)	60.9	3.53 (m)	60.9	6´
	3.75 (dd, 10.5)		3.75 (dd, 10.5, 10.5)		
6	4.46 (d, 14.2)	52.3	4.49 (d, 14.0)	52.3	1´
	4.00 (d, 14.2)		4.03 (d, 14.0)		
7		135.3		135.1	5
8	4.93 (d, 15.6)	58.1	4.93 (d, 16.0)	58.1	6
	4.85 (d, 15.6)		4.86 (d, 16.0)		
9	6.06 (d, 4.1)	105.3	6.08 (d, 4.0)	105.3	4
10	6.98 (d, 4.1)	124.1	6.99 (d, 4.0)	124.1	3
11		132.1		132.2	2
12	9.47 (s)	179.1	9.48 (s)	179.1	7

[a] Carbohydrate numbering system used herein. [b] Referenced to residual solvent signal from acetone-d_6 at 2.05 ppm for ^{1}H-NMR (600 MHz), and 29.84 ppm for ^{13}C-NMR (151 MHz). [c] Reported in ref. 11 as referenced to TMS. [d] Original numbering system used in ref.11.

Table S6. Other comparative analytical data for pollenopyrroside A.

**data**	**synthetic sample (1)**	**literature**[11]
optical rotation	$[\alpha]_D^{19}$: +128.7° (*c* 0.08, MeOH)	$[\alpha]_D^{20}$: +125.9° (*c* 0.08, MeOH)
LRMS	ESI+: 276.0 ($[M+Na]^+$, 90)	ESI+: 254.1 ($[M+H]^+$)
HRMS found	ESI: 276.0835 ([M+Na]+)	n.a.
HRMS calcd	ESI: 276.0848 ($[M+Na]^+$)	n.a.

2. SHENSONGINE A (2)

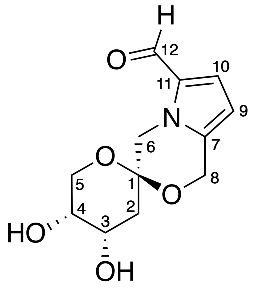

2
carbohydrate numbering herein

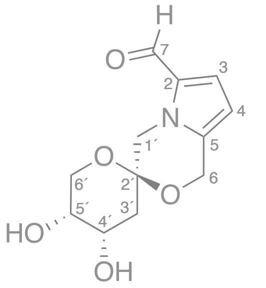

shensongine A
original numbering (Yao, et al.*)*

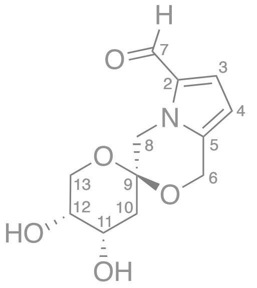

xylapyroside A
original numbering (Hu, et al.*)*

Table S7. Comparative ^{1}H- and ^{13}C-NMR data for synthetic (2) and isolated shensongine A, and xylapyrroside A in CD_3OD.

	synthetic sample (2)[b]		shensongine A[c] (Yao, *et al.*)[16]		xylapyrroside A[b] (Hu, *et al.*)[17]	
peak[a]	δ_H (*J* values in Hz)	δ_C	δ_H	δ_C	δ_H	δ_C
1		96.7		96.7		96.9
2	1.99 (dd, 12.7, 11.7)	36.1	1.98 (t, 12.0)	36.1	2.02 (dd, 12.8, 11.6)	36.1
	1.90 (dd, 12.6, 5.0)		1.89 (dd, 12.0, 5.3)		1.92 (dd, 12.8, 5.2)	
3	4.07 (ddd, 11.7, 5.2, 3.0)	66.1	4.07 (ddd, 12.0, 5.3, 2.7)	66.0	4.10 (ddd, 11.6, 5.2, 2.8)	66.0
4	3.78 (m, 2.6)	68.7	3.78 (overlapped)	68.7	3.81 (m)	68.7
5	3.81 (dd, 12.0, 1.1)	66.3	3.79 (dd, overlapped)	66.3	3.83 (d, 12.0)	66.3
	3.76 (dd, 12.0, 2.1)		3.67 (dd, 18.5, 7.3)[c]		3.78 (d, 12.0)	
6	4.60 (d, 13.9)	53.5	4.58 (d, 14.0)	53.4	4.62 (d, 14.0)	53.5
	3.97 (d, 13.9)		3.96 (d, 14.0)		4.00 (d, 14.0)	
7		137.1		137.0		137.1
8	4.84 (d, 15.7)	58.6	4.83 (overlapped)	58.6	4.86 (d, 16.0)	58.6
	4.74 (d, 15.8)		4.73 (d, 15.8)		4.77 (d, 16.0)	
9	6.07 (d, 4.1)	106.1	6.06 (d, 4.1)	106.1	6.09 (d, 4.0)	106.1
10	7.02 (d, 4.1)	125.9	7.00 (d, 4.1)	125.7	7.04 (d, 4.0)	125.8
11		132.4		132.4		132.4
12	9.36 (s)	180.2	9.35 (s)	180.2	9.39 (s)	180.2

[a] Carbohydrate numbering system used herein. [b] Referenced to residual solvent signal from CD_3OD at 3.31 ppm for ^{1}H-NMR, and 49.00 ppm for ^{13}C-NMR. [c] NMR spectra of Ref. 16 unavailable for direct comparison.

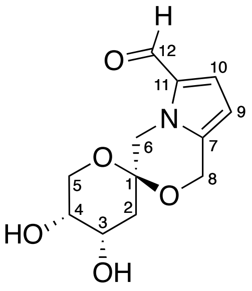

2
carbohydrate numbering herein

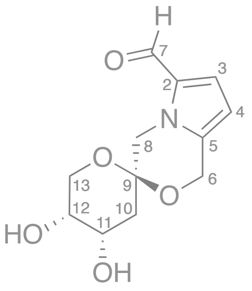

xylapyrroside A
original numbering (Hu, et al.*)*

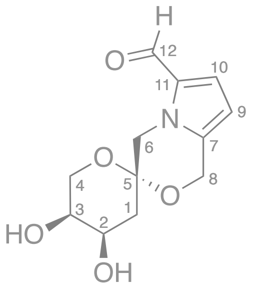

capparisine B
original numbering (Wang, et al.*)*

Table S8. Comparative ^{1}H- and ^{13}C-NMR data for synthetic (2) and isolated xylapyrroside A and capparisine B in $CDCl_3$.

	**synthetic sample (2)**[b]		**xylapyrroside A**[c] (Hu, *et al.*)[17]	**capparisine B**[d] (Wang, *et al.*)[12]	
**peak**[a]	**δ_H (*J* values in Hz)**	**δ_C**	**δ_H**	**δ_H**	**δ_C**
1		95.4			95.4
2	1.92 (dd, 13.0, 11.5)	35.7	1.90 (dd, 12.8, 11.6)	1.91 (t, 12.4)	35.8
	2.05 (dd, 13.0, 5.4)		2.02 (dd, 12.8, 5.6)	2.04 (dd, 12.8, 5.3)	
3	4.17 (m)	65.0	4.14 (ddd, 11.6, 5.6, 2.8)	4.17 (m)	65.1
4	3.89 (m, overlapped)	67.3	3.87 (m, overlapped)	3.88 (s)	67.4
5	3.89 (dd, overlapped)	64.5	3.87 (dd, overlapped)	3.89 (d, 12.1)	64.5
	3.81 (dd, 12.7, 1.3)		3.78 (dd, 12.8, 1.2)	3.81 (d, 12.1)	
6	4.69 (d, 14.1)	52.2	4.68 (d, 13.9)	4.70 (d, 14.4)	52.2
	4.02 (d, 14.1)		3.99 (d, 13.9)	4.02 (d, 13.8)	
7		134.1			134.1
8	4.82 (d, 15.4)	57.7	4.80 (d, 15.6)	4.82 (d, 15.2)	57.8
	4.74 (d, 15.5)		4.71 (d, 15.6)	4.74 (d, 15.7)	
9	6.01 (d, 4.1)	104.8	5.99 (d, 4.0)	6.01 (d, 3.5)	104.8
10	6.92 (d, 4.1)	124.0	6.90 (d, 4.0)	6.92 (d, 3.8)	124.0
11		131.1			131.2
12	9.45 (s)	178.8	9.42 (s)	9.45 (s)	178.8

[a] Carbohydrate numbering system used herein. [b] Referenced to residual solvent signal from $CDCl_3$ at 7.26 ppm for ^{1}H-NMR, and 77.16 ppm for ^{13}C-NMR. [c] ^{13}C-NMR spectrum of xylapyrroside A in $CDCl_3$ for Ref. 17 unavailable for direct comparison. [d] NMR spectra of Ref. 12 unavailable for direct comparison.

Table S9. Other comparative analytical data for shensongine A.

data	synthetic sample (2)	shensongine A	xylapyrroside A	capparisine B
optical rotation	$[\alpha]_D^{20}$: –38.8° (*c* 0.9, MeOH)	$[\alpha]_D^{27}$: –12.7° (*c* 0.05, MeOH)	$[\alpha]_D^{22}$: –189° (*c* 0.1, MeOH)	$[\alpha]_D^{25}$: +37.9° (*c* 0.1, MeOH)
LRMS	ESI+: 276.0 ($[M+Na]^+$, 100)	ESI+: 254 ($[M+H])^+$	ESI+: 254 ($[M+H])^+$	n.a.
HRMS found	ESI: 276.0848 ($[M+Na]^+$)	ESI: 254.1024 ($[M+H]^+$)	ESI: 253.0952 ($[M+H]^+$)	ESI: 253.0954 ($[M+H]^+$)
HRMS calcd	ESI: 276.0858 ($[M+Na]^+$)	ESI: 254.1028 ($[M+H]^+$)	ESI: 253.0950 ($[M+H]^+$)	ESI: 253.0950 ($[M+H]^+$)

O. DOSE-RESPONSE CURVES FOR ANTIOXIDANT ACTIVITY STUDIES

1. ANTIOXIDANT ACTIVITY OF ACORTATARIN A (3)

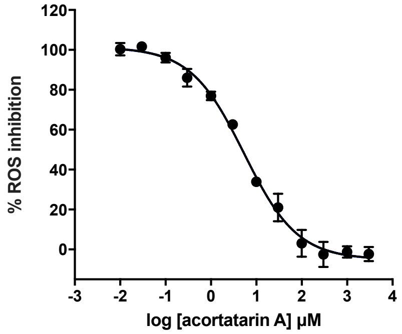

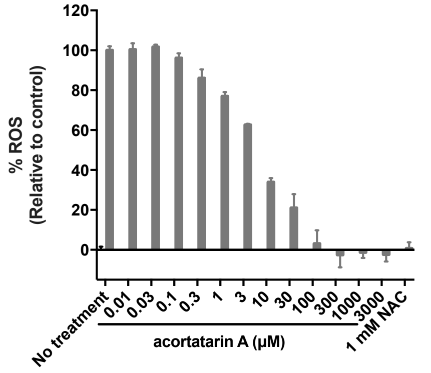

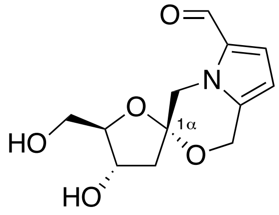

	acortatarin A
IC50	5.346

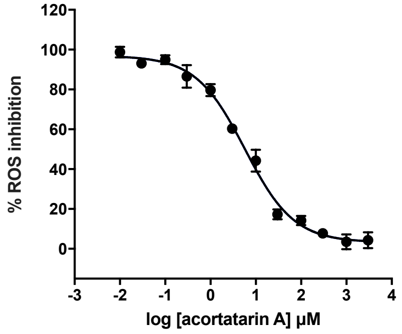

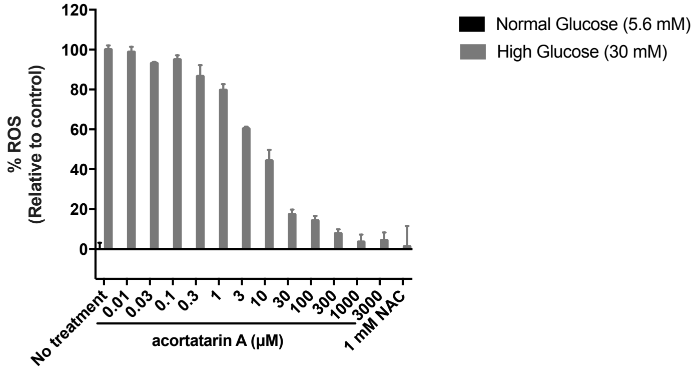

	acortatarin A
IC50	5.74

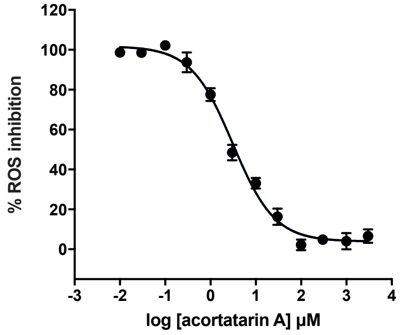

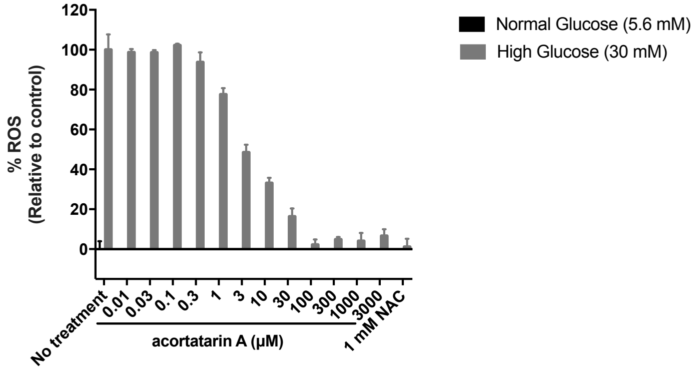

	acortatarin A
IC50	3.109

2. Antioxidant activity of shensongine B (5)

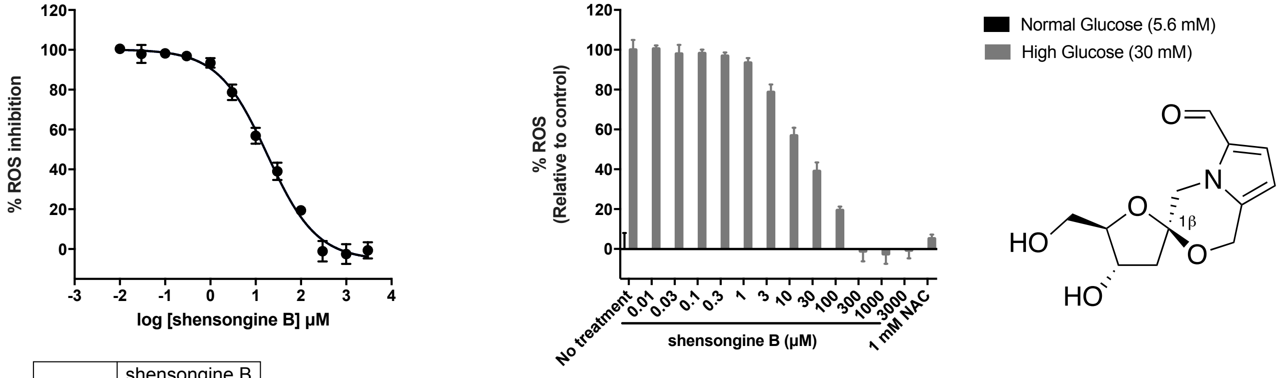

	shensongine B
IC50	18.14

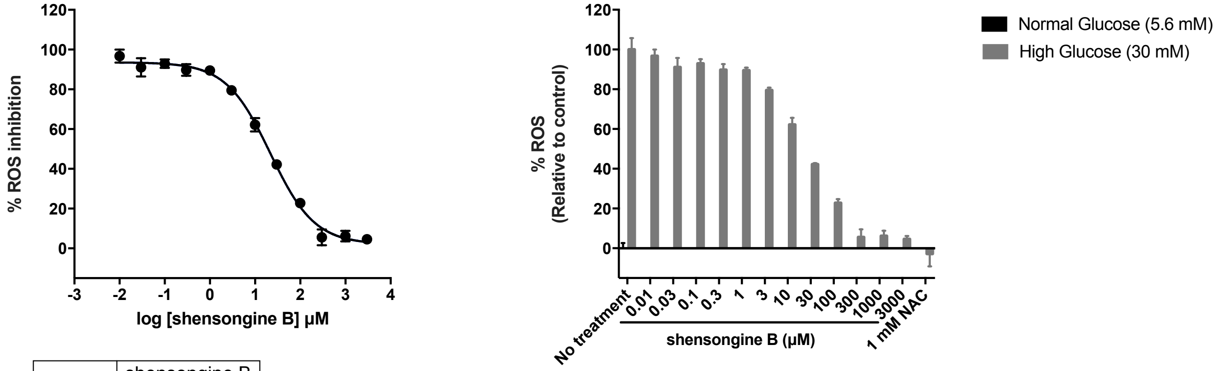

	shensongine B
IC50	21.74

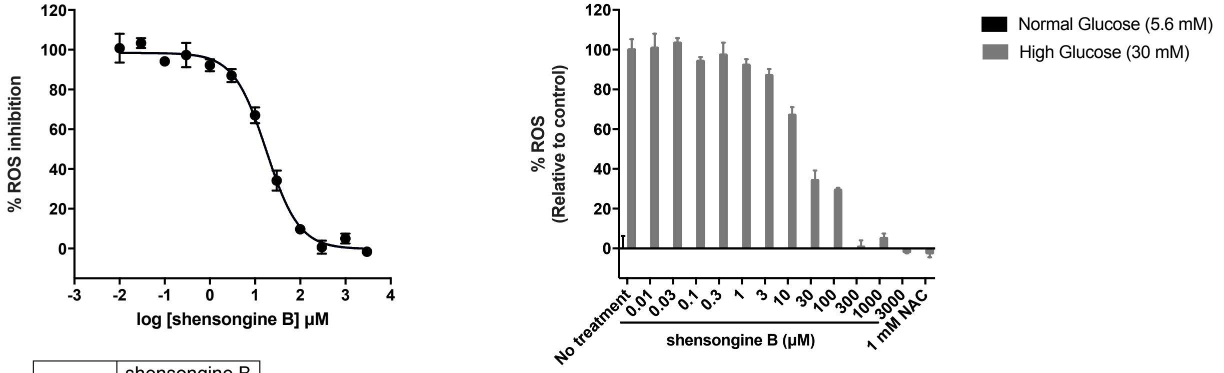

	shensongine B
IC50	17.83

3. Antioxidant activity of shensongine C (4)

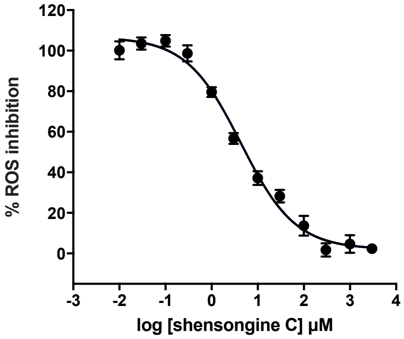

	shensongine C
IC50	4.32

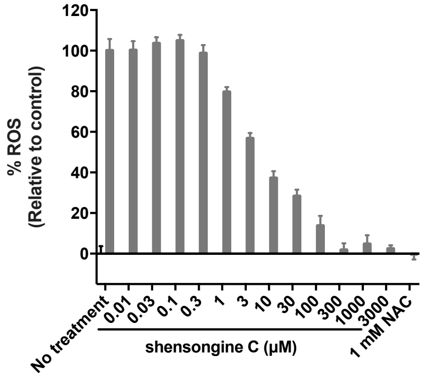

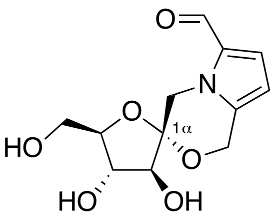

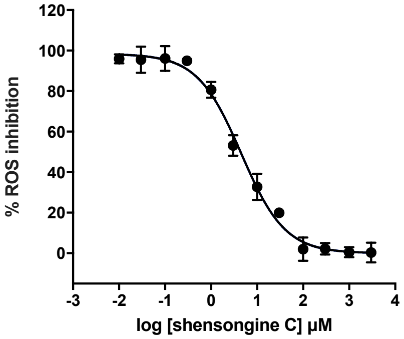

	shensongine C
IC50	4.521

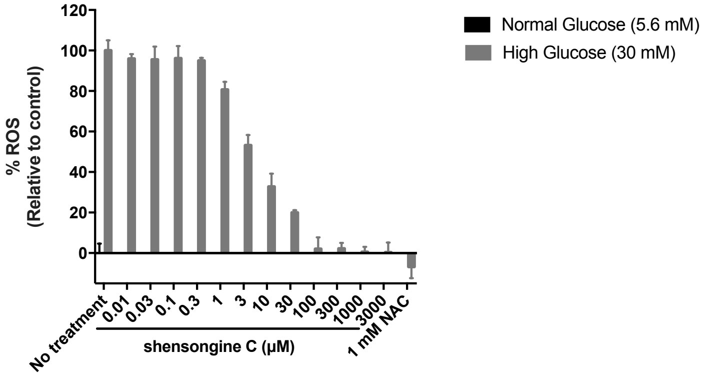

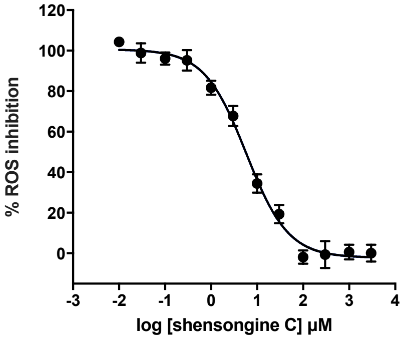

	shensongine C
IC50	5.819

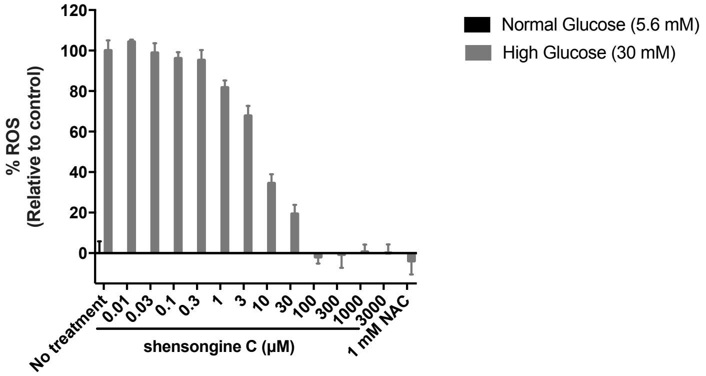

4. Antioxidant activity of acortatarin B (6)

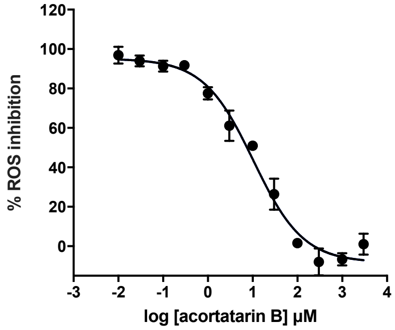

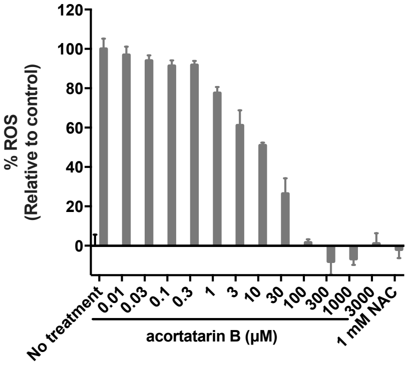

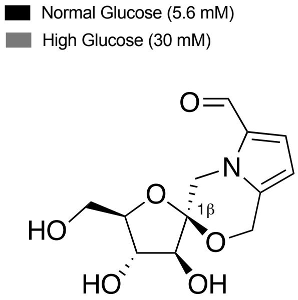

	acortatarin B
IC50	10.38

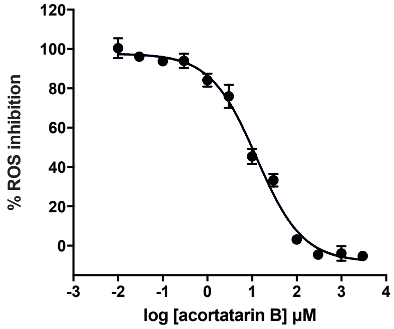

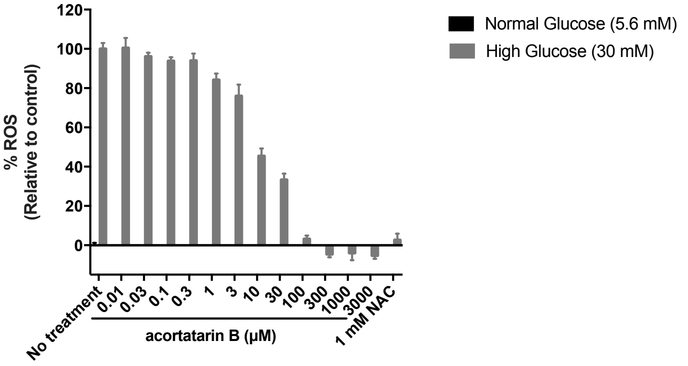

	acortatarin B
IC50	12.62

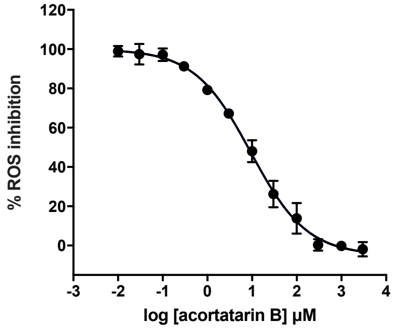

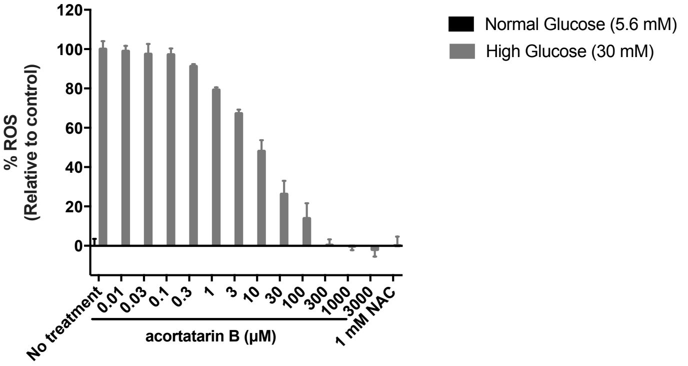

	acortatarin B
IC50	9.352

5. ANTIOXIDANT ACTIVITY OF POLLENOPYRROSIDE A (1)

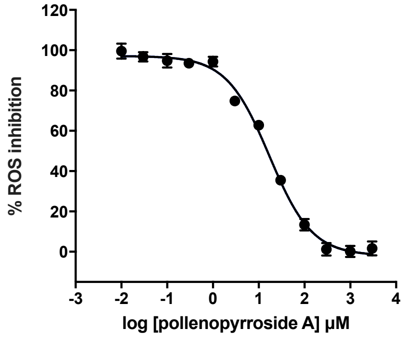

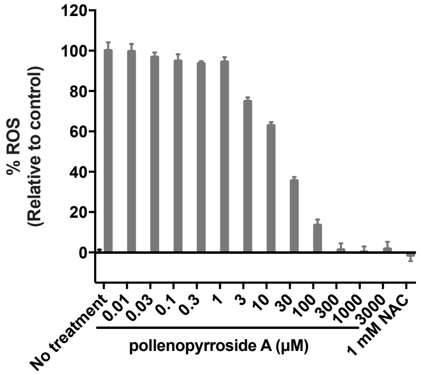

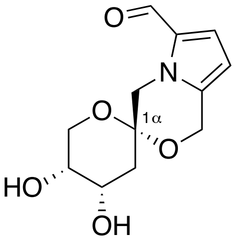

	pollenopyrroside A
IC50	17.06

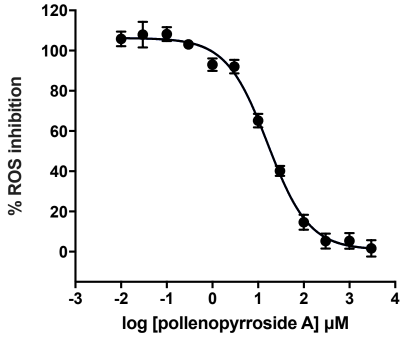

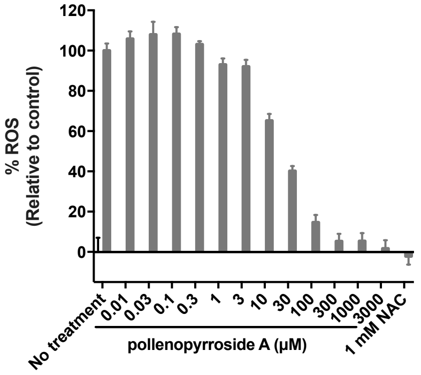

	pollenopyrroside A
IC50	16.24

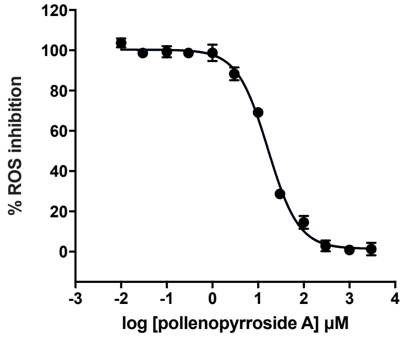

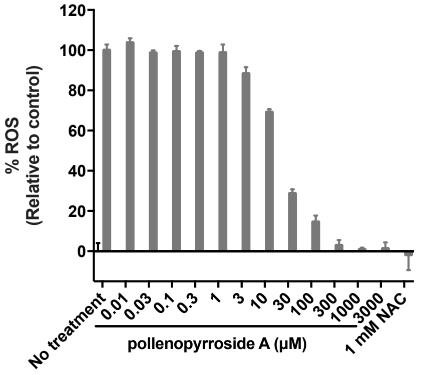

	pollenopyrroside A
IC50	16.43

6. ANTIOXIDANT ACTIVITY OF SHENSONGINE A (2)

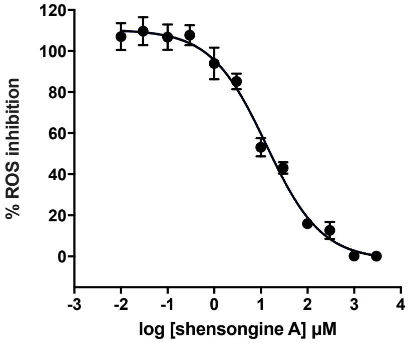

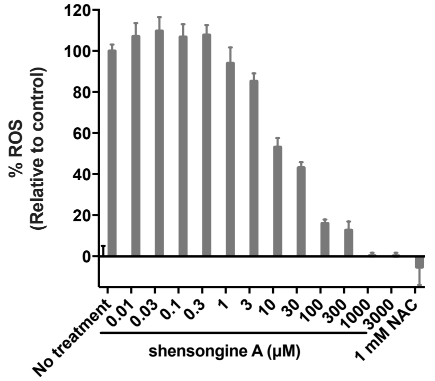

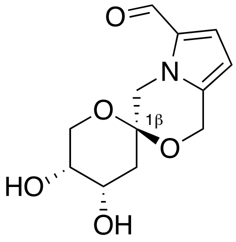

	shensongine A
IC50	13.06

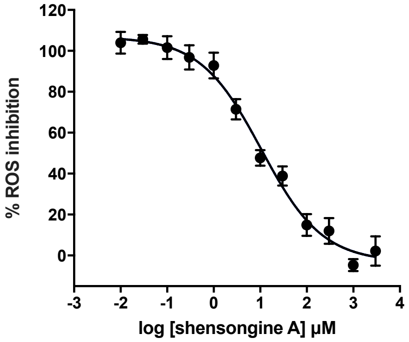

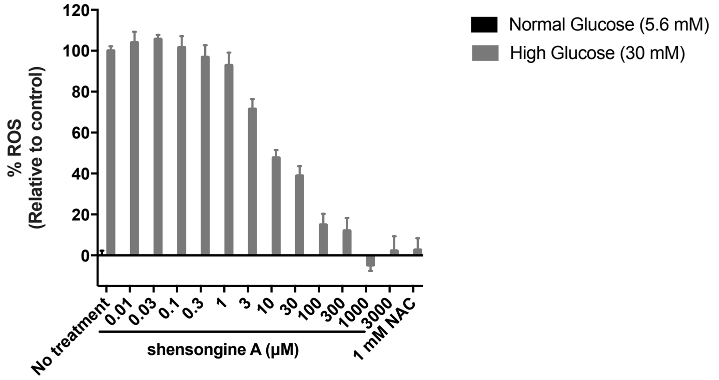

	shensongine A
IC50	10.55

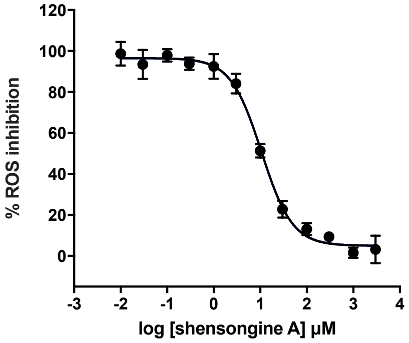

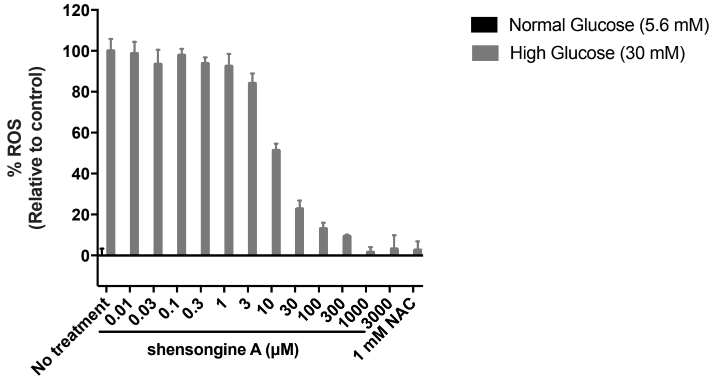

	shensongine A
IC50	10.64

7. ANTIOXIDANT ACTIVITY OF 2-OH POLLENOPYRROSIDE A (25)

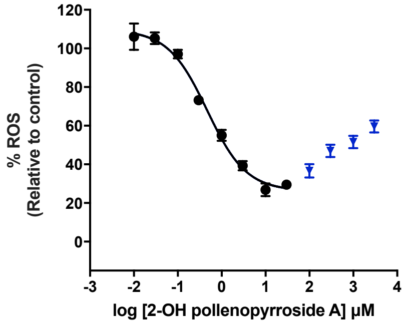

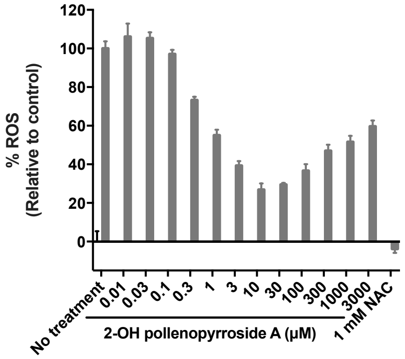

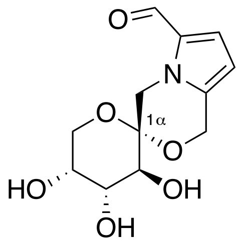

	2-OH pollenopyrroside A
IC50	0.4669

▼ Data points excluded from curve fit

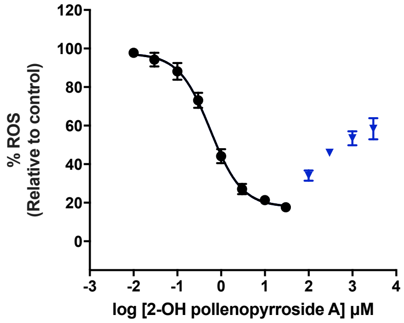

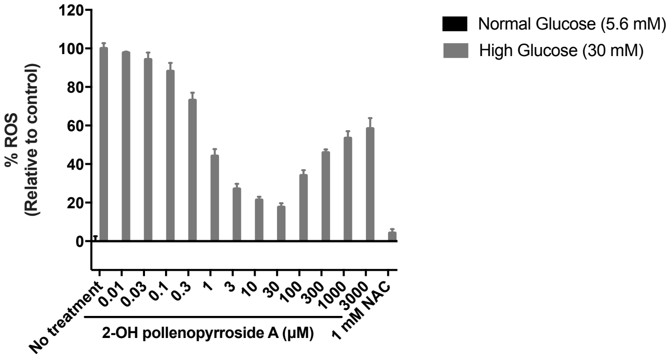

	2-OH pollenopyrroside A
IC50	0.5705

▼ Data points excluded from curve fit

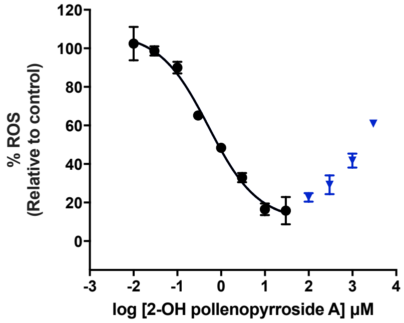

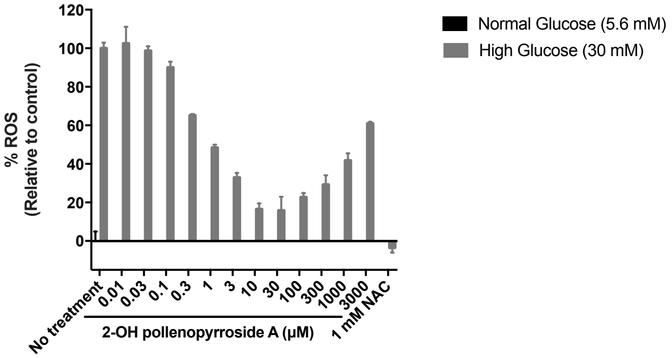

	2-OH pollenopyrroside A
IC50	0.5185

▼ Data points excluded from curve fit

8. Antioxidant activity of 2-OH shensongine A (28)

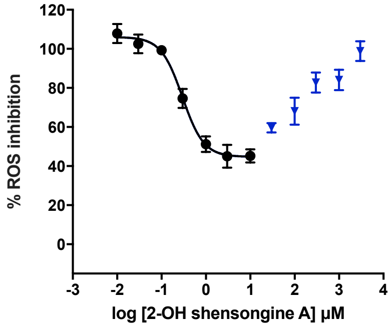

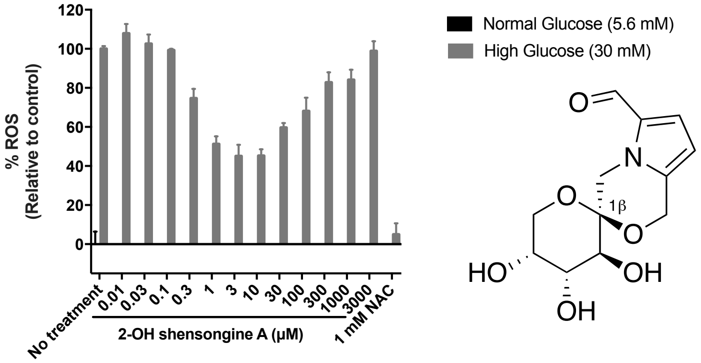

	2-OH shensongine A
IC50	0.295

▼ Data points excluded from curve fit

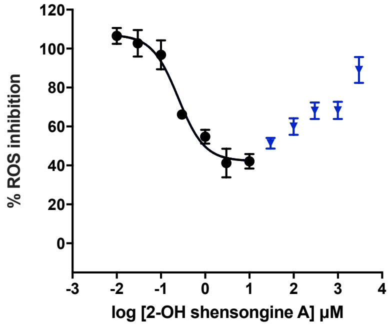

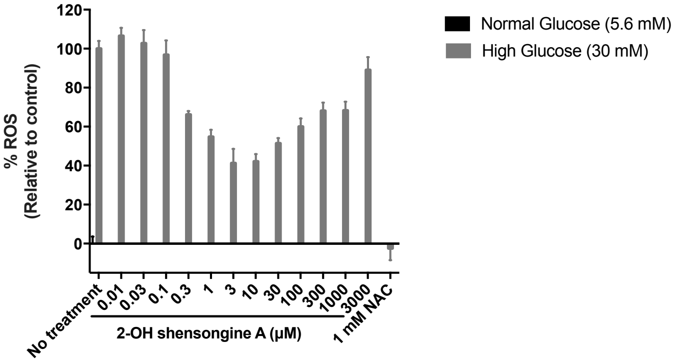

	2-OH shensongine A
IC50	0.246

▼ Data points excluded from curve fit

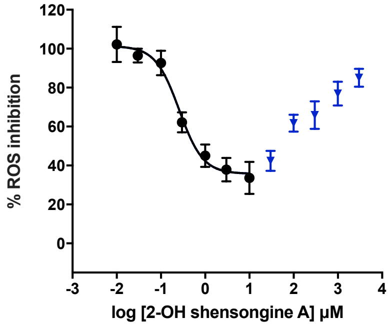

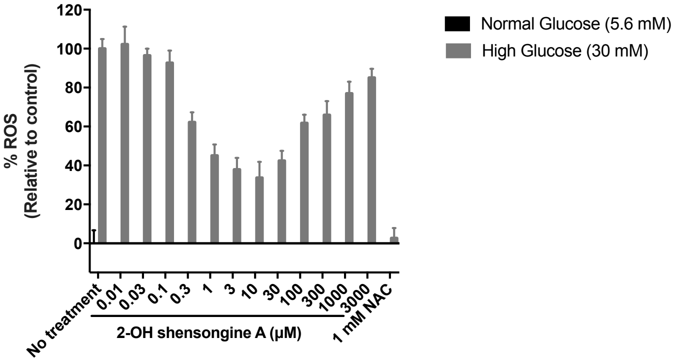

	2-OH shensongine A
IC50	0.2582

▼ Data points excluded from curve fit

P. ^{1}H-NMR AND ^{13}C-NMR SPECTRA

1. SYNTHESIS OF *C*1-MESYL-HYDROXYMETHYL-D-ARABINAL (14b) **S40**

a. TIPS-protected D-arabinal **12** S40
b. TIPS-protected *C*1-formyl-D-arabinal **S2** S41
c. *C*1-Hydroxymethyl TIPS-protected D-arabinal **13** S42
d. *C*1-Mesyl-hydroxymethyl TIPS-protected D-arabinal **14b** S43

2. SYNTHESIS OF *C*1-PYRROLOMETHYL-D-ARABINAL SUBSTRATES (15, 16, 18) **S44**

a. TIPS-protected pyrrole dicarboxaldehyde pyranoglycal **15** S44
b. TIPS-protected pyrrole monoalcohol pyranoglycal **16** S45
c. Pyrrole monoalcohol pyranoglycal **18** S46

3. SYNTHESIS OF 2-MERCURIAL SPIROKETALS (20b, 22b) **S47**

a. TIPS-protected 2-mercurial spiroketal **20b** S47
b. 2-Mercurial spiroketal **22b** S48

4. SYNTHESIS OF SHENSONGINE A (2) **S49**

a. TIPS-protected β-spiroketal **17** S49
b. Shensongine A (**2**) S50

5. SYNTHESIS OF POLLENOPYRROSIDE A (1) **S53**

a. TIPS-protected 2-hydroxy-α-spiroketal **24** S53
b. TIPS-protected 2-xanthate-α-spiroketal **26** S54
c. TIPS-protected α-spiroketal **23** S55
d. Pollenopyrroside A (**1**) S56

6. SYNTHESIS OF 2-HYDROXY ANALOGUES (25, 28) **S57**

a. 2-OH pollenopyrroside A (**25**) S57
b. TIPS-protected 2-hydroxy-β-spiroketal **27** S58
b. 2-OH shensongine A (**28**) S59

TIPS-protected D-arabinal (12) 1H-NMR (600 MHz, CDCl3)

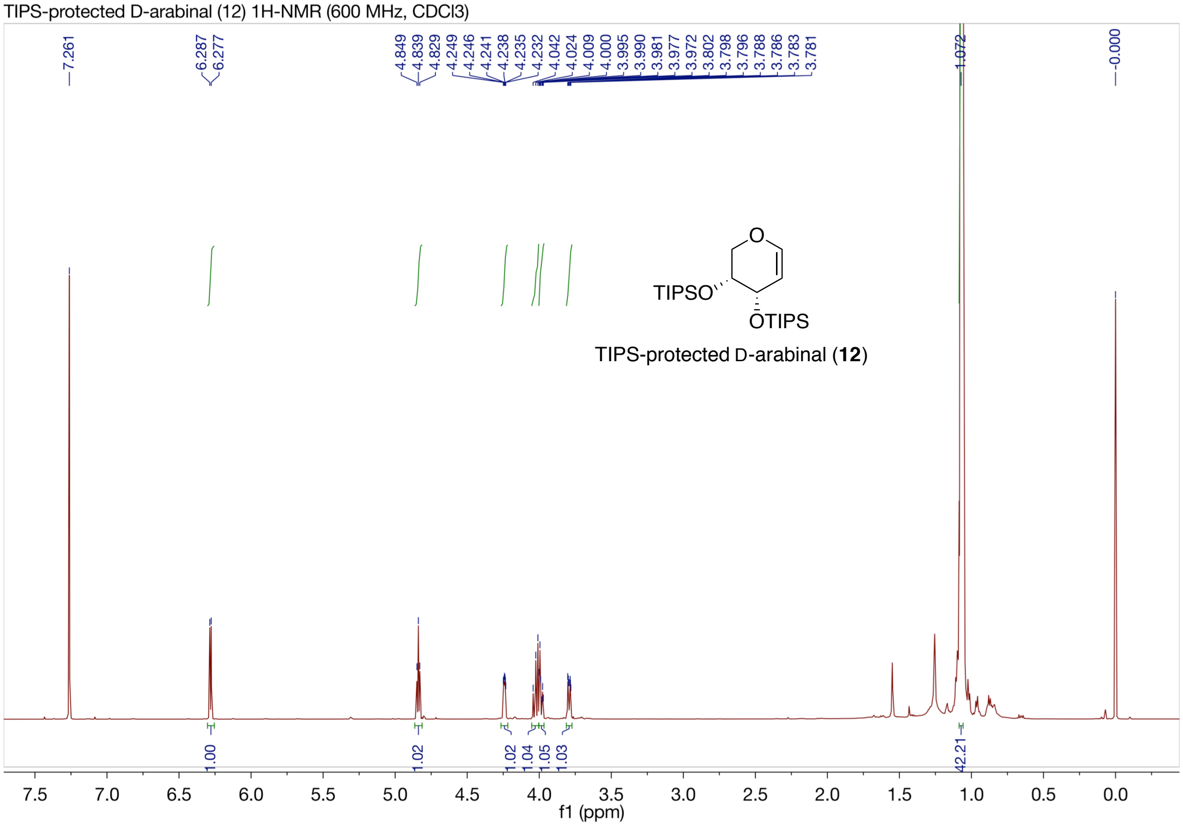

TIPS-protected D-arabinal (12) 13C-NMR (151 MHz, CDCl3)

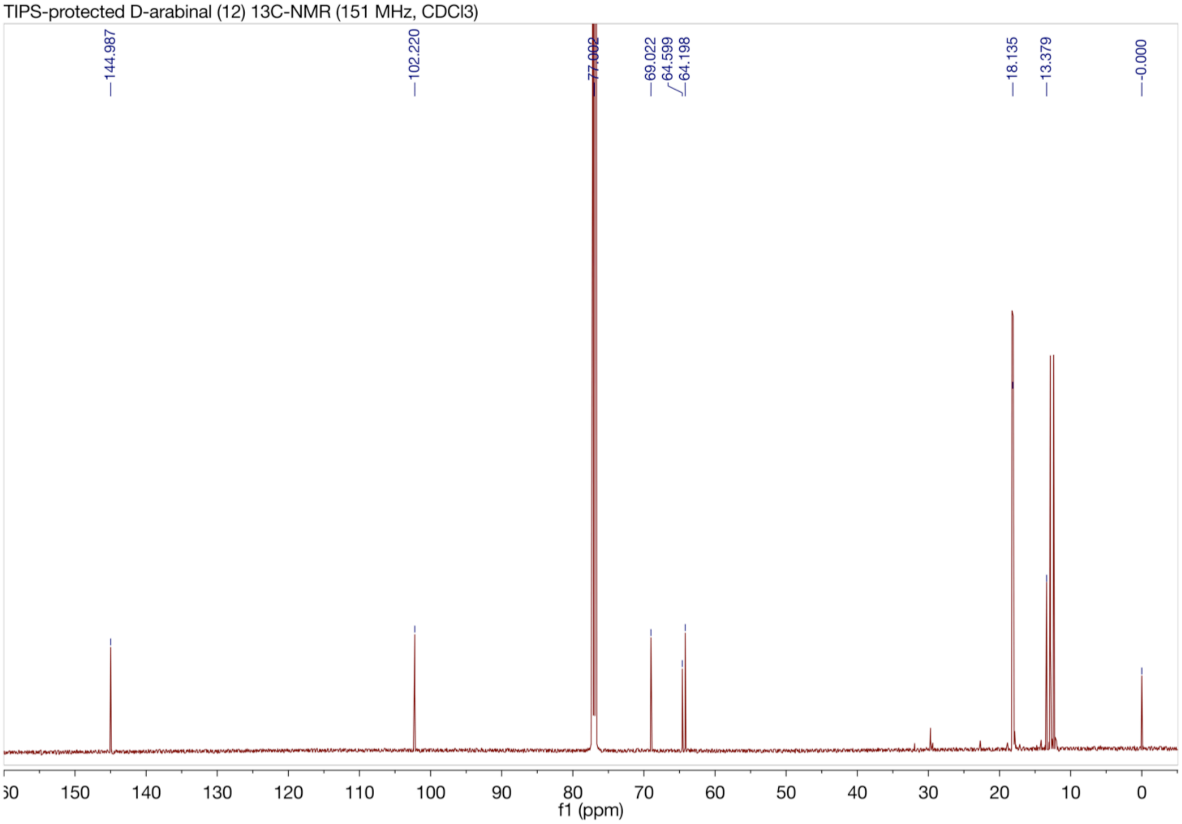

TIPS-protected C1-formyl-D-arabinal (S2) 1H-NMR (600 MHz, CDCl3)

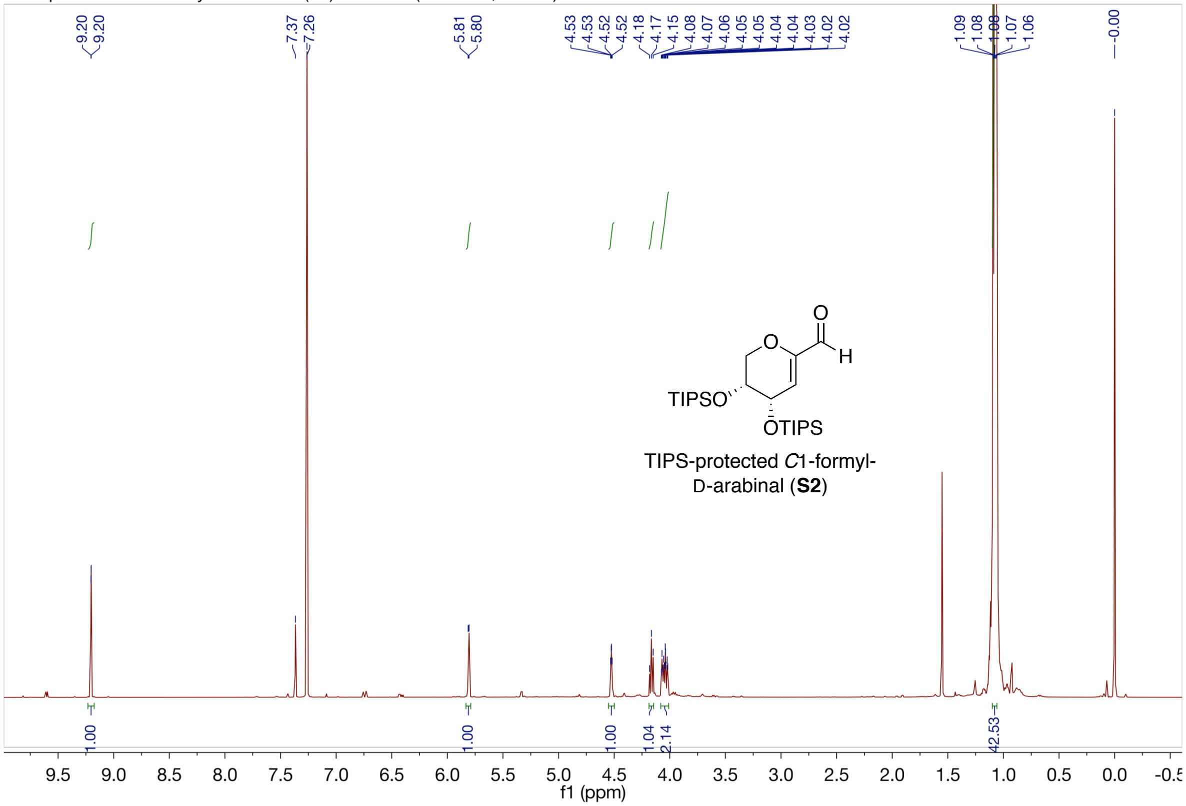

TIPS-protected C1-formyl-D-arabinal (S2) 13C-NMR (151 MHz, CDCl3)

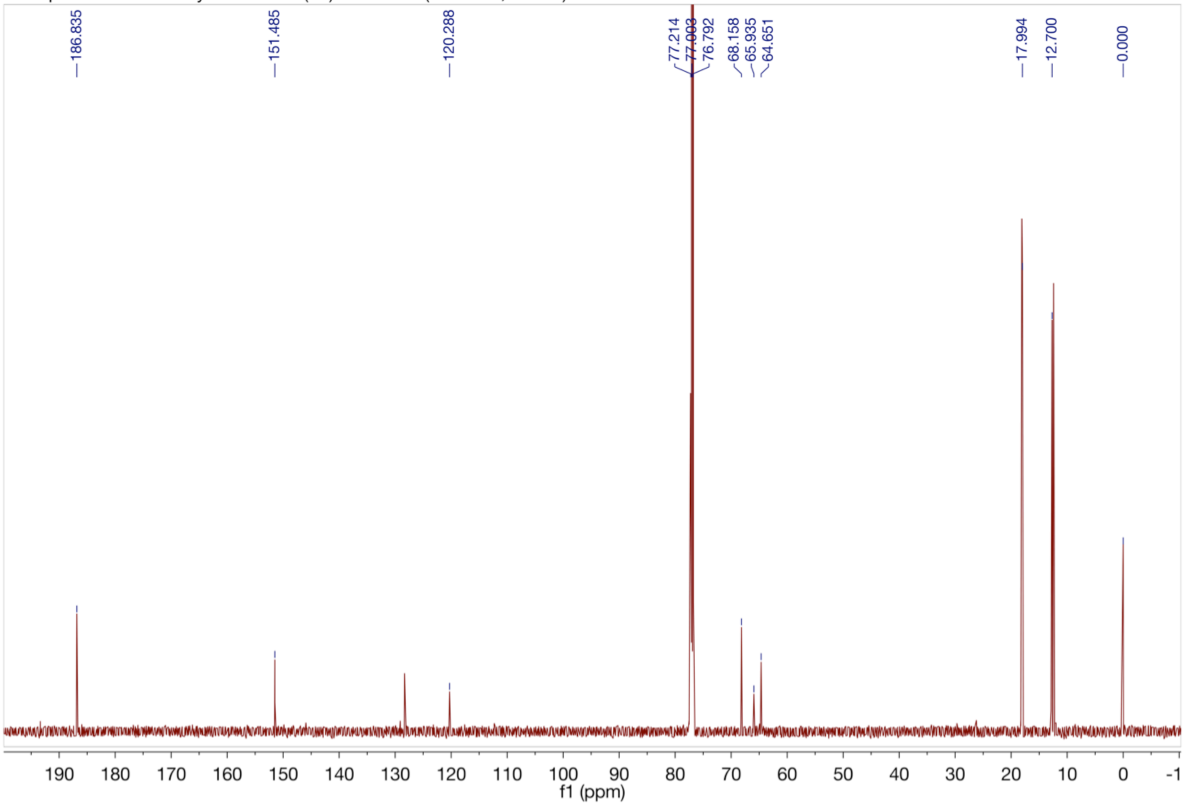

TIPS-protected C1-hydroxymethyl-D-arabinal (13) 1H-NMR (600 MHz, CDCl3)

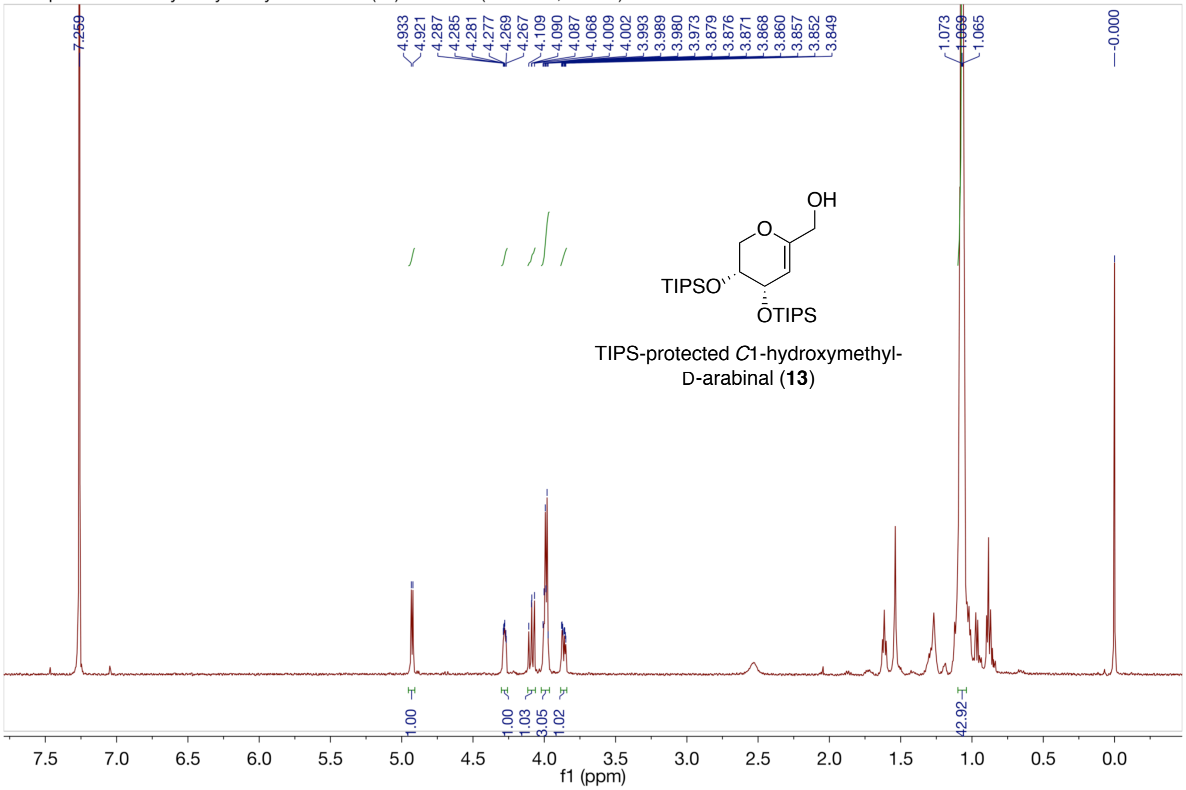

TIPS-protected C1-hydroxymethyl-D-arabinal (13) 13C-NMR (151 MHz, CDCl3)

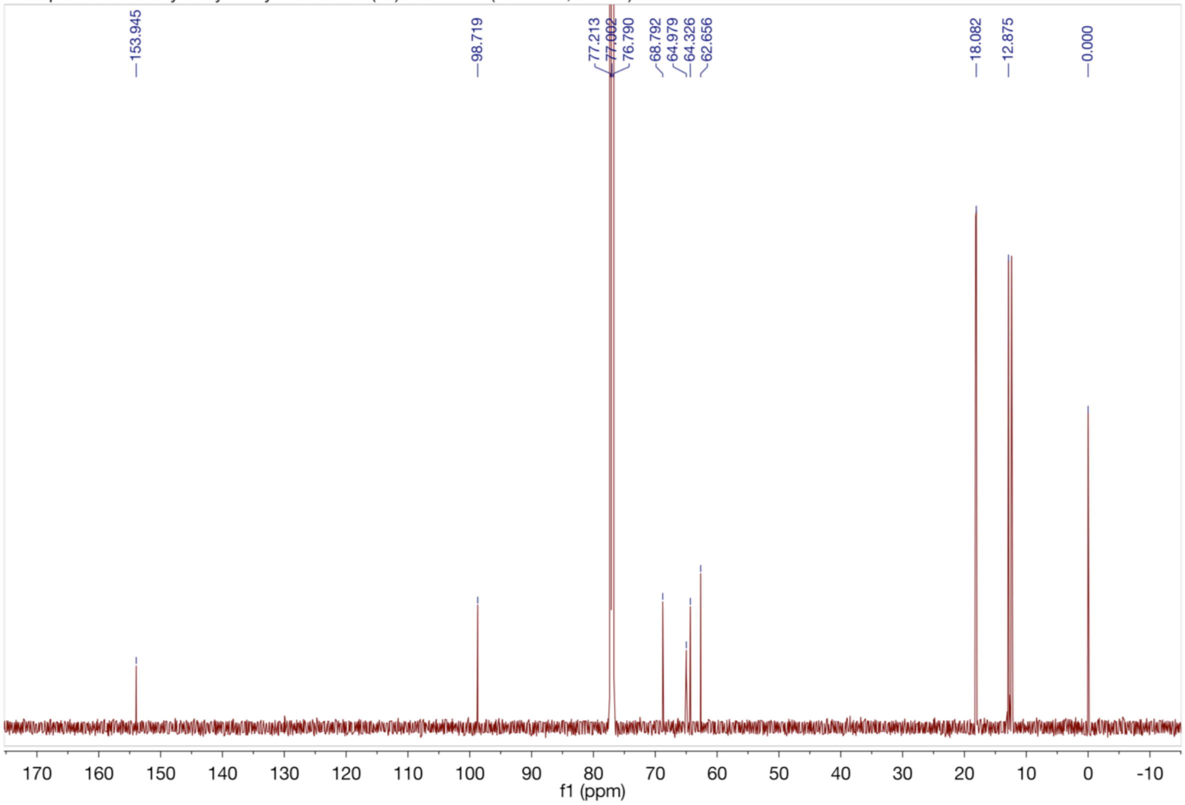

TIPS-protected C1-mesyl-hydroxymethyl-D-arabinal (14b) 1H-NMR (600 MHz, CDCl3)

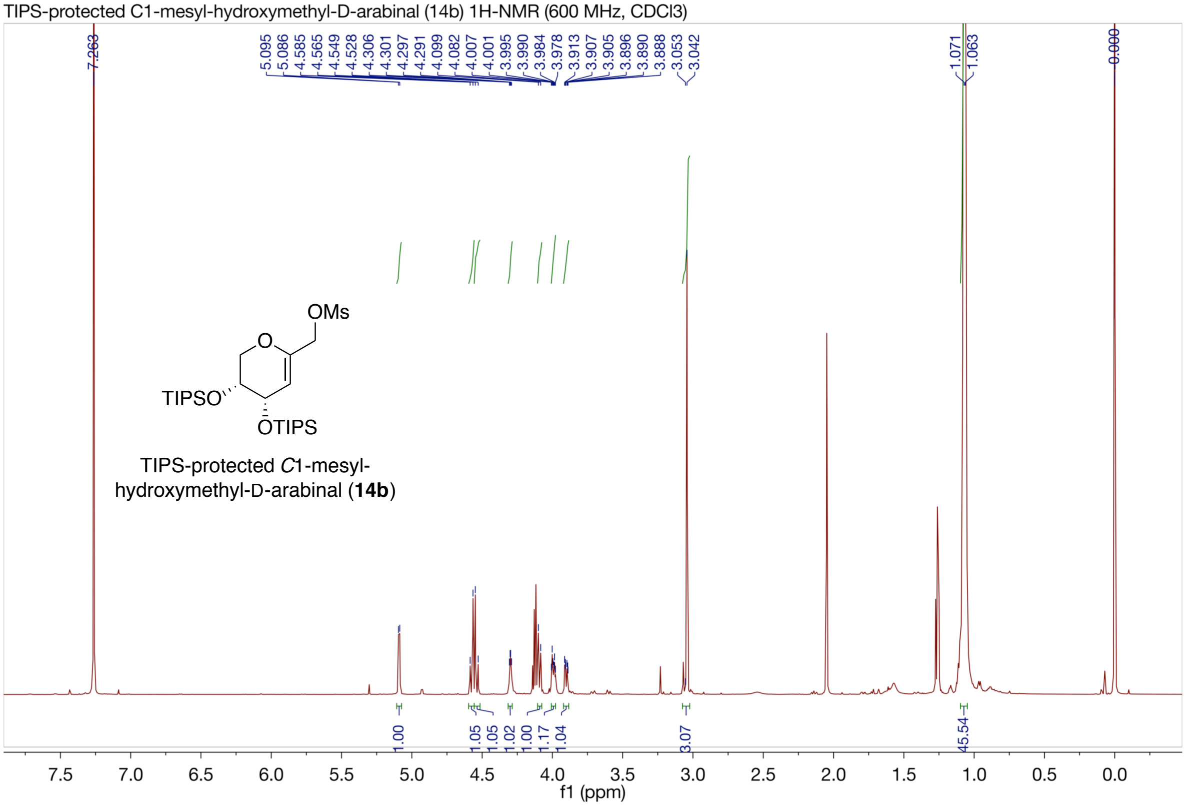

TIPS-protected C1-mesyl-hydroxymethyl-D-arabinal (14b) 13C-NMR (151 MHz, CDCl3)

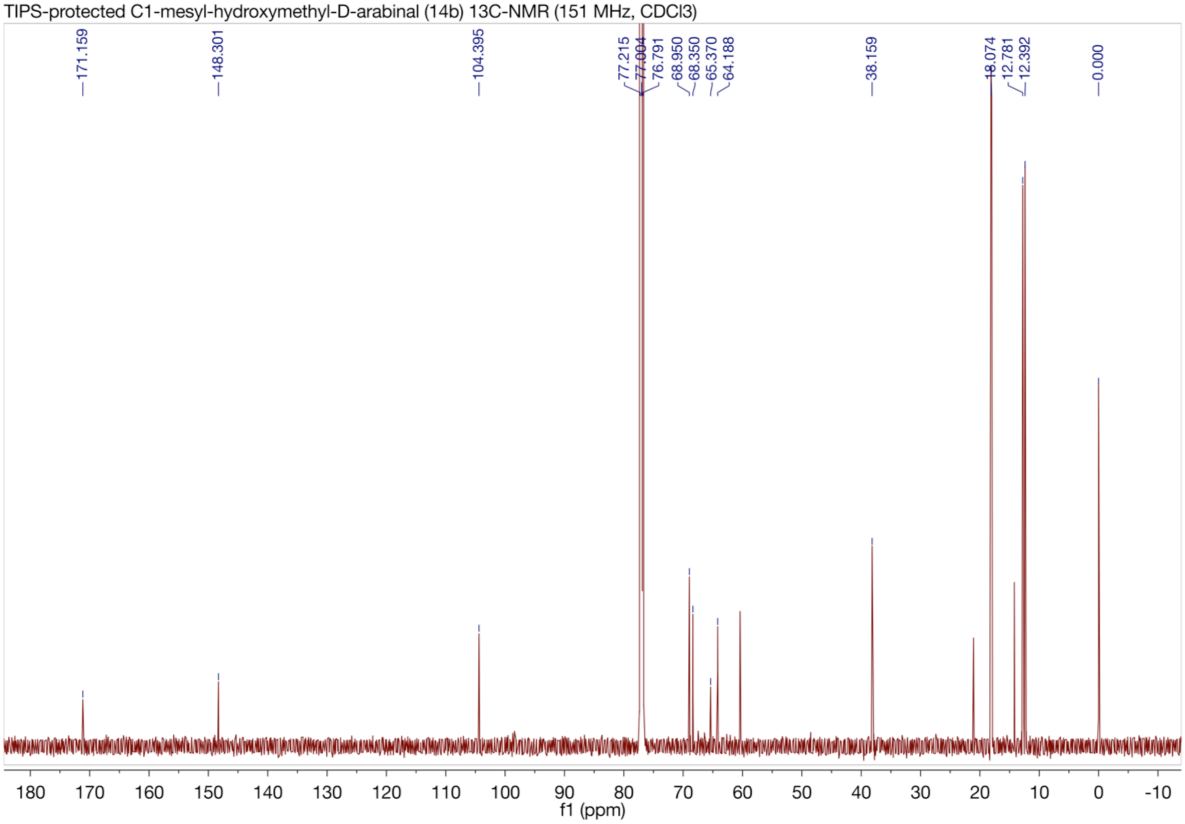

TIPS-protected pyrrole dicarboxaldehyde pyranoglycal (15) 1H-NMR (600 MHz, CDCl3)

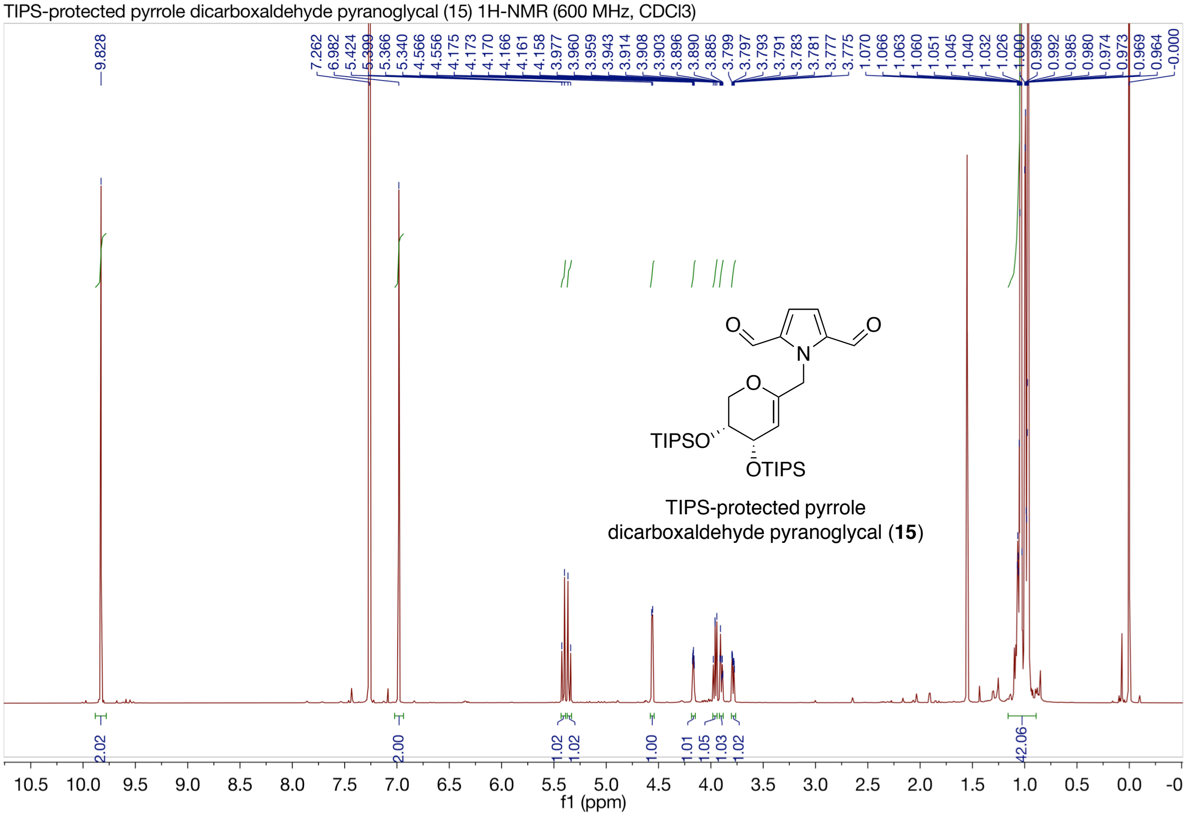

TIPS-protected pyrrole dicarboxaldehyde pyranoglycal (15) 13C-NMR (151 MHz, CDCl3)

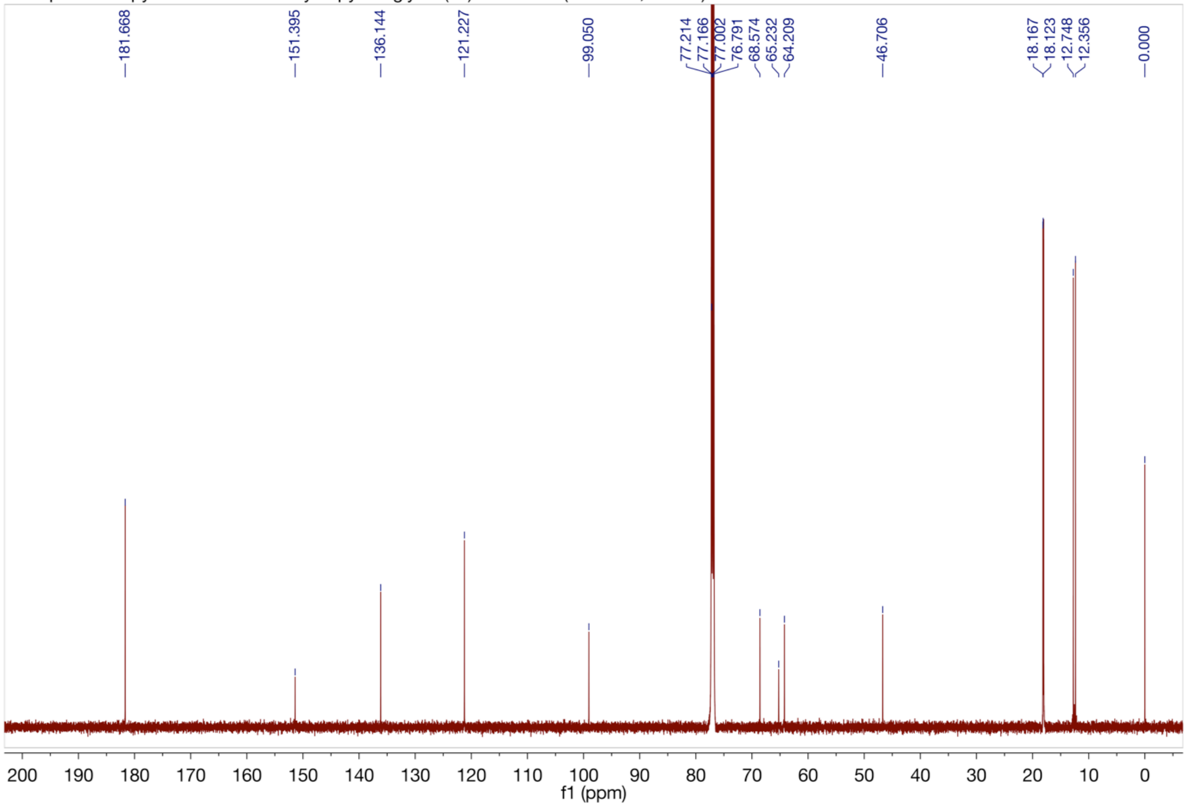

TIPS-protected pyrrole monoalcohol pyranoglycal (16) 1H-NMR (600 MHz, CDCl3)

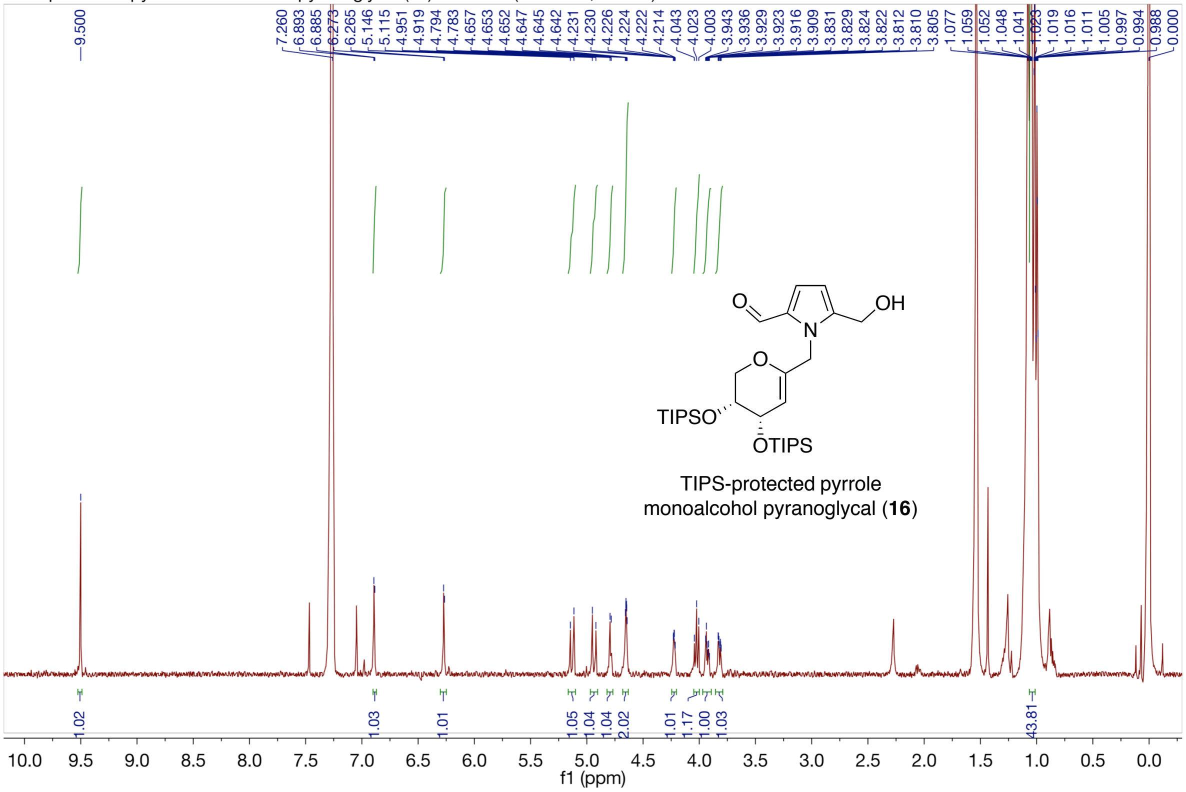

TIPS-protected pyrrole monoalcohol pyranoglycal (16) 13C-NMR (151 MHz, CDCl3)

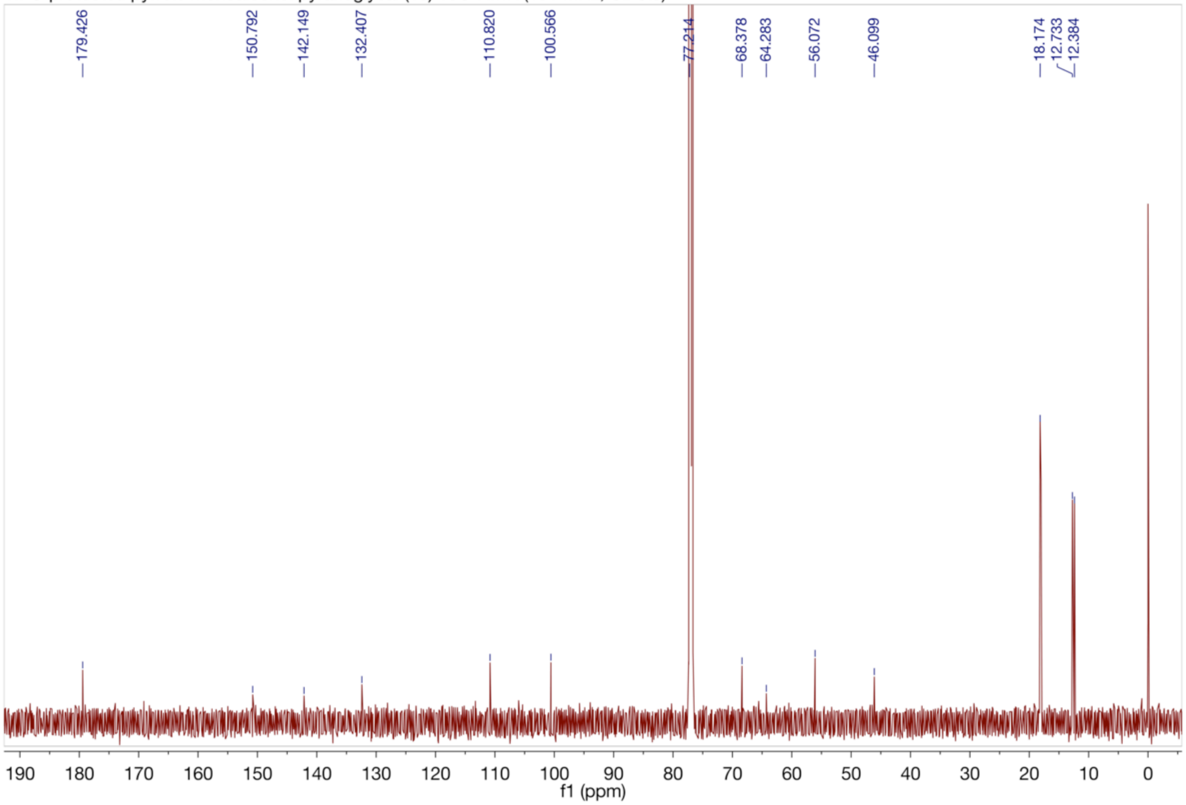

Pyrrole monoalcohol pyranoglycal (18) 1H-NMR (600 MHz, CD3OD)

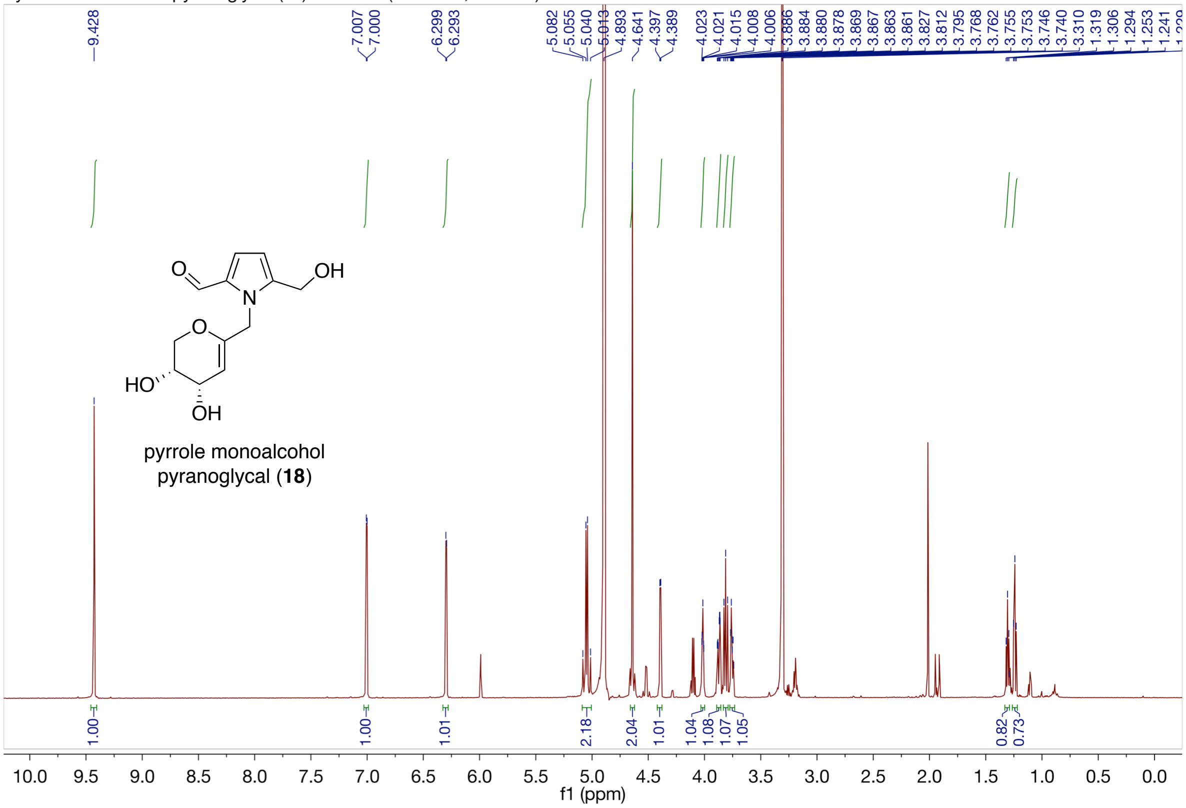

Pyrrole monoalcohol pyranoglycal (18) 13C-NMR (151 MHz, CD3OD)

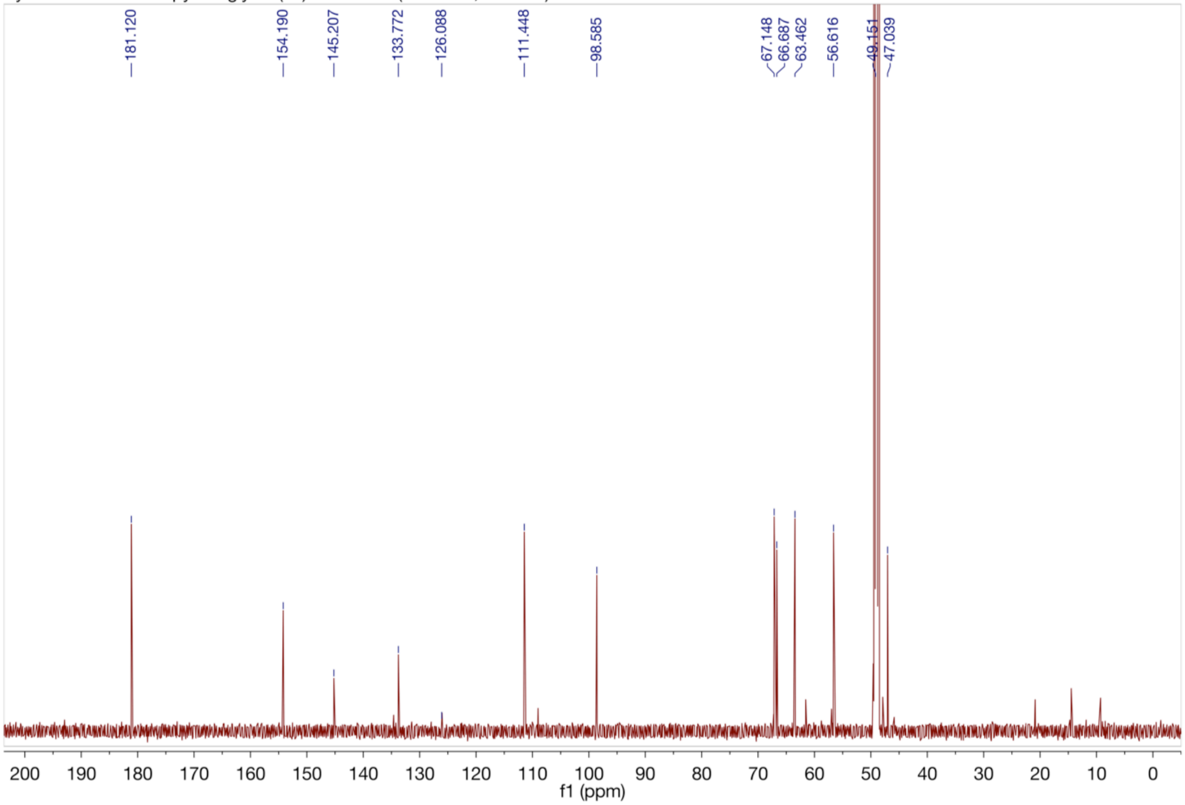

TIPS-protected 2-mercurial β-spiroketal, crude (20b) 1H-NMR (600 MHz, CDCl3)

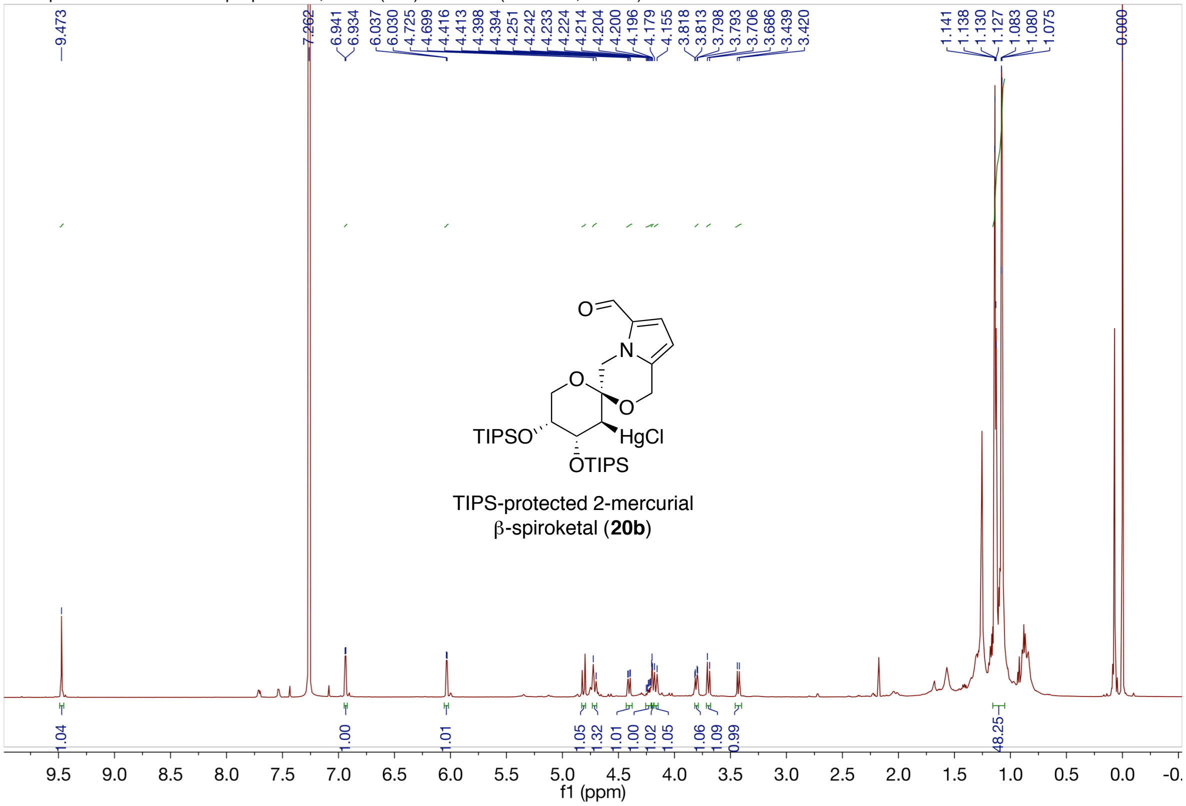

TIPS-protected 2-mercurial β-spiroketal, crude (20b) 13C-NMR (151 MHz, CDCl3)

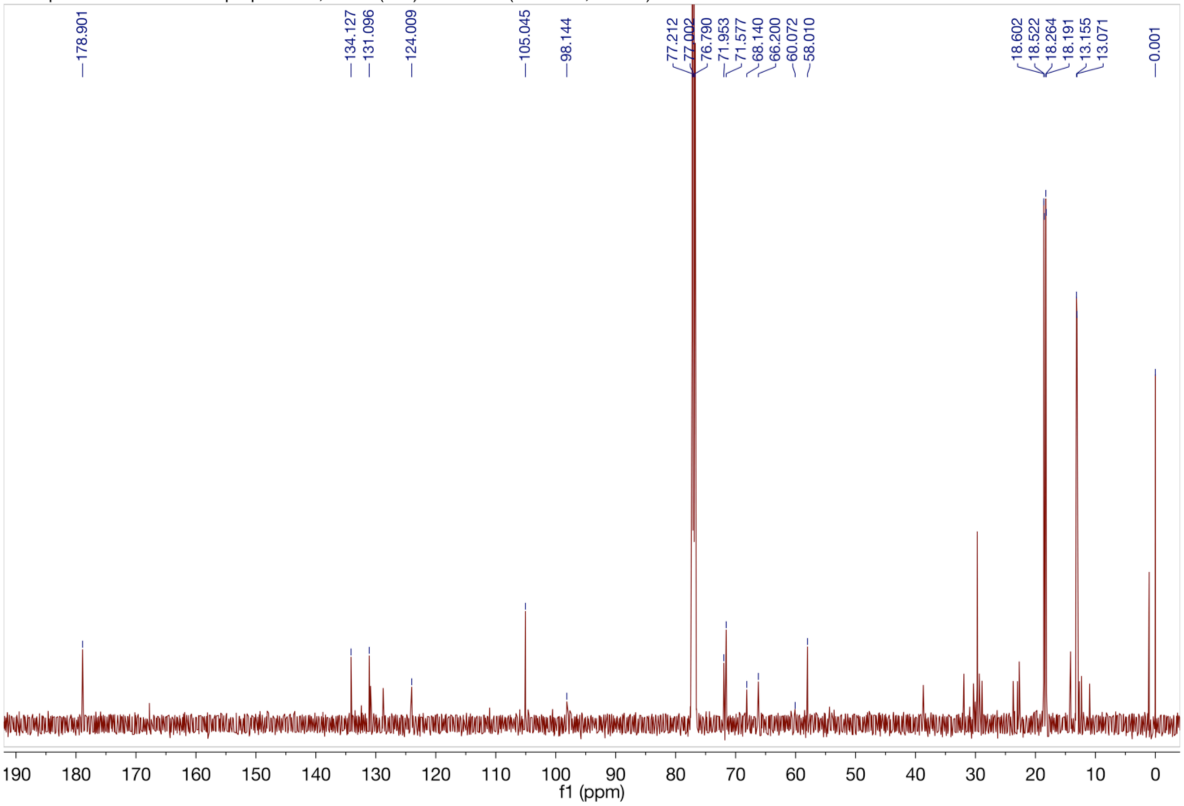

2-mercurial β-spiroketal, crude (22b) 1H-NMR (600 MHz, CD3OD)

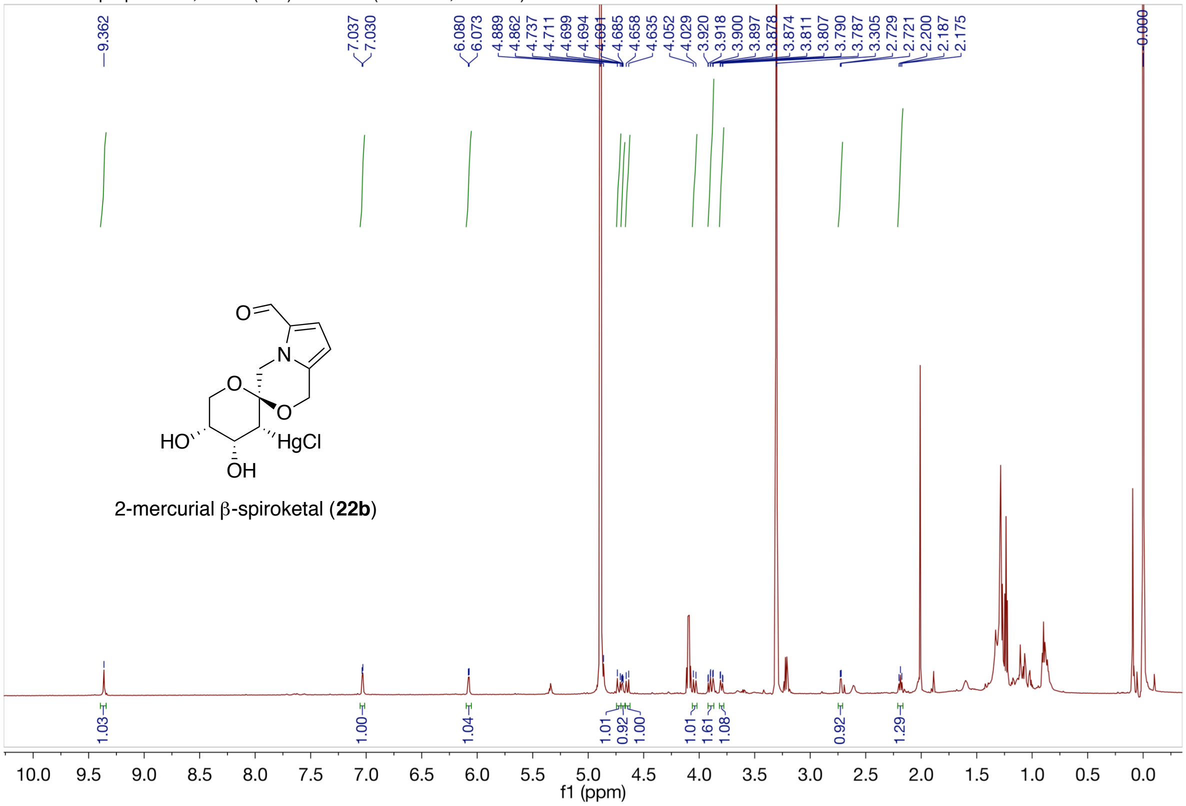

2-mercurial β-spiroketal (22b) 13C-NMR (151 MHz, CD3OD)

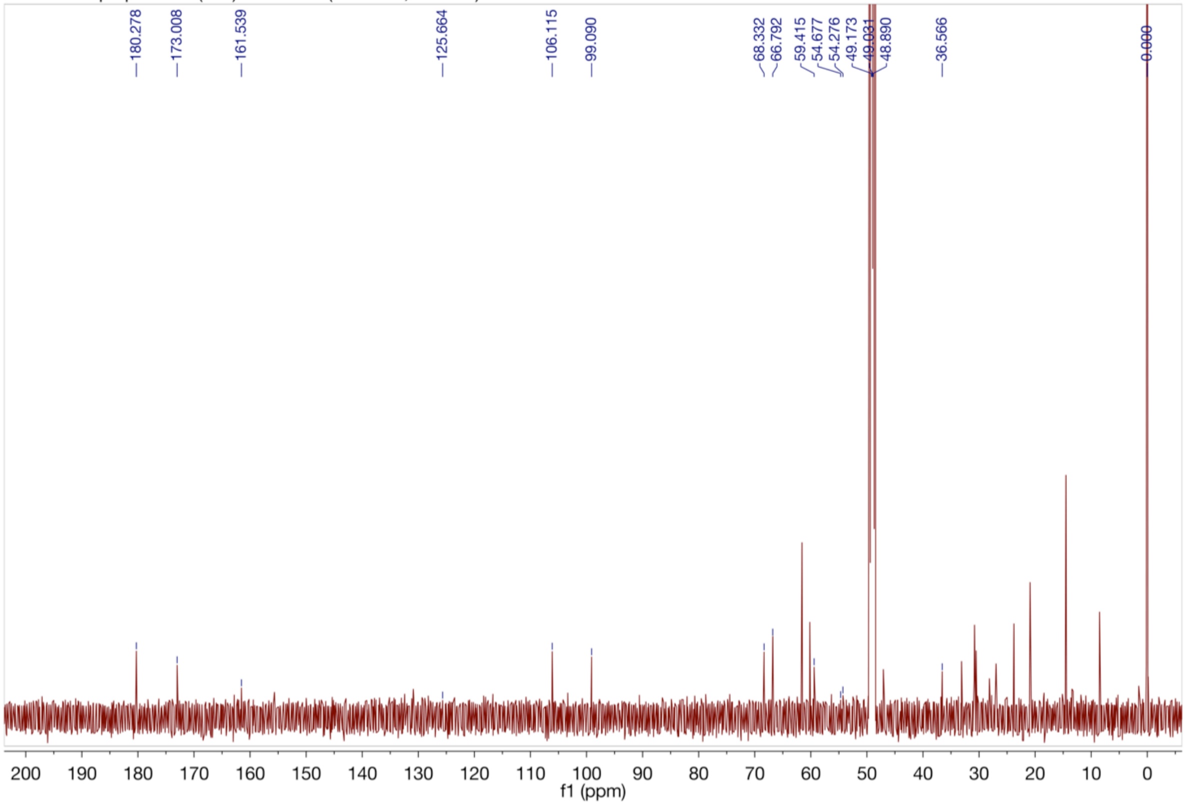

TIPS-protected β-spiroketal (17) 1H-NMR (600 MHz, CDCl3)

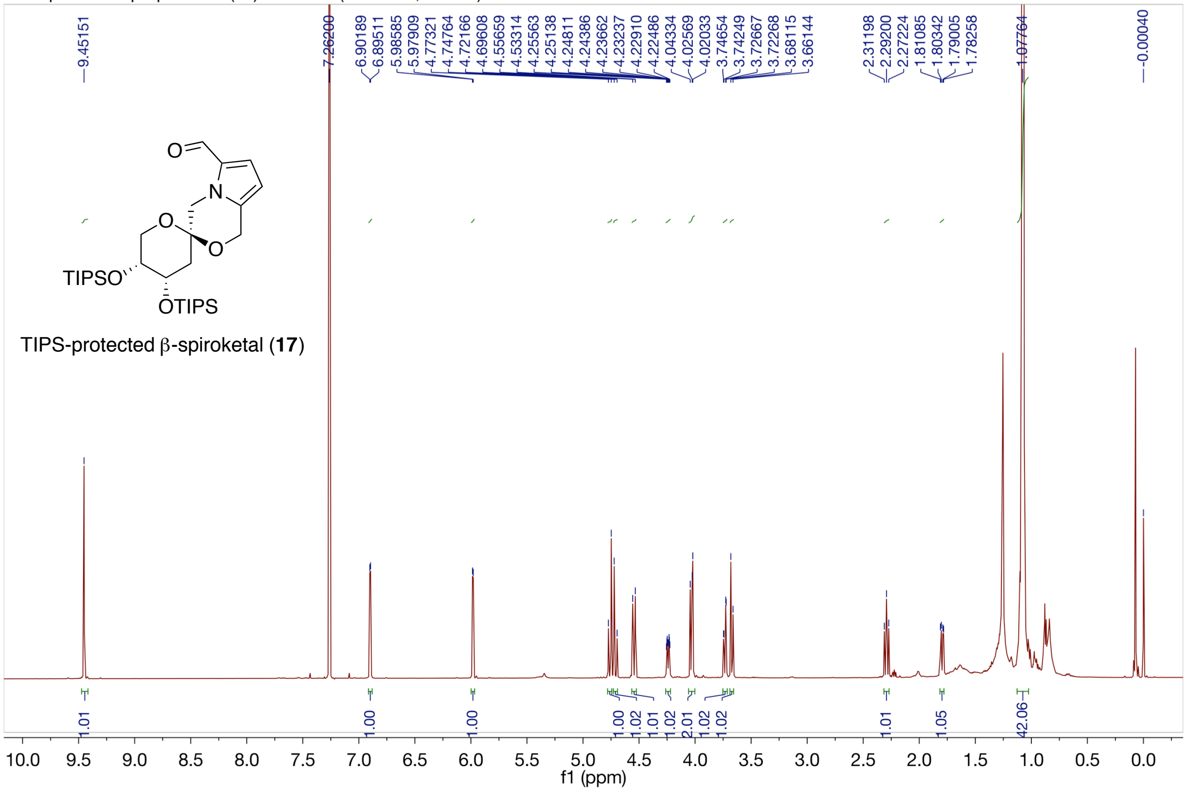

TIPS-protected β-spiroketal (17) 13C-NMR (151 MHz, CDCl3)

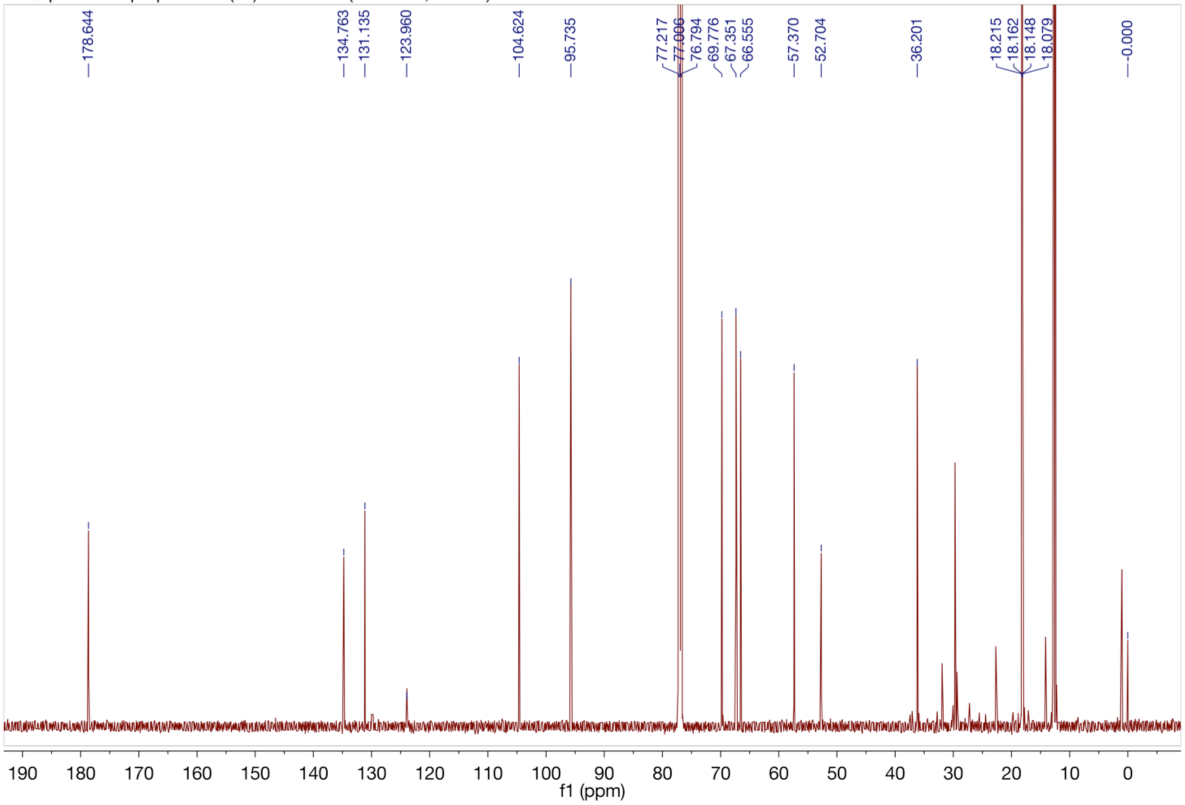

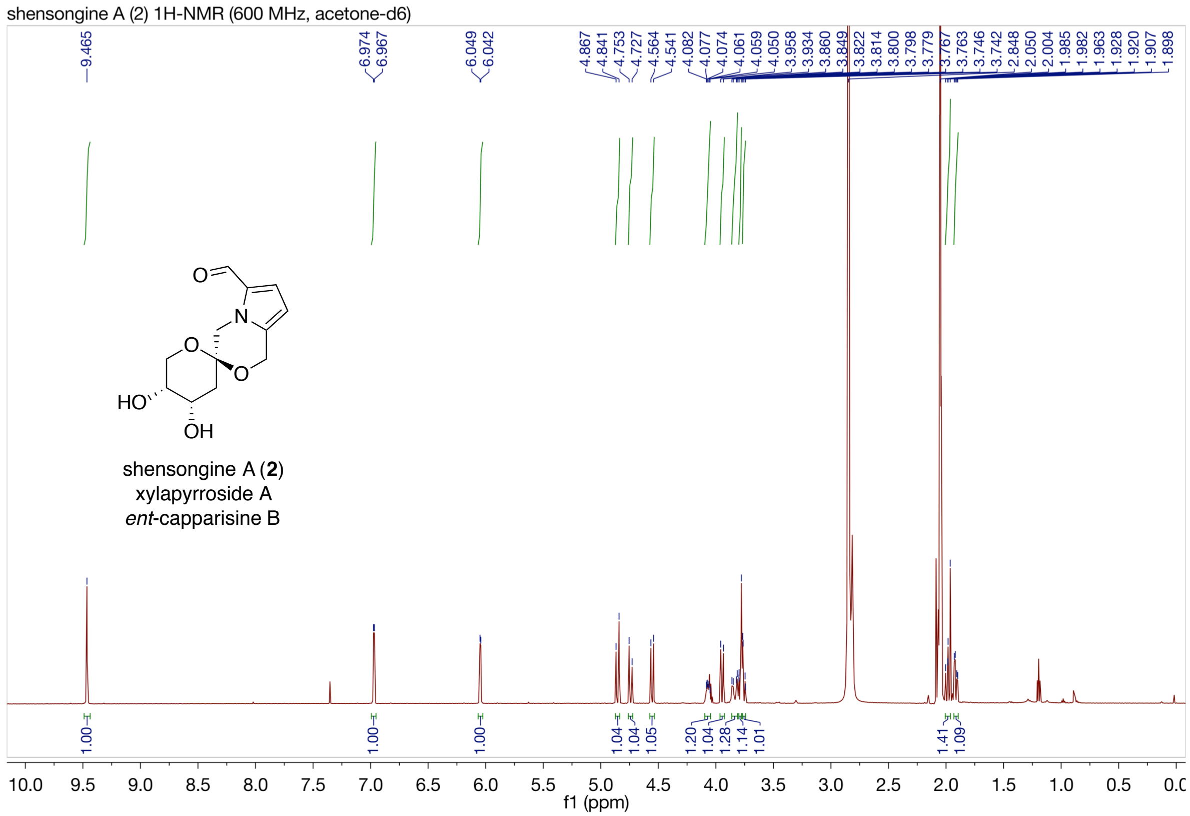
shensongine A (2) 1H-NMR (600 MHz, acetone-d6)
shensongine A (**2**)
xylapyrroside A
ent-capparisine B
f1 (ppm)

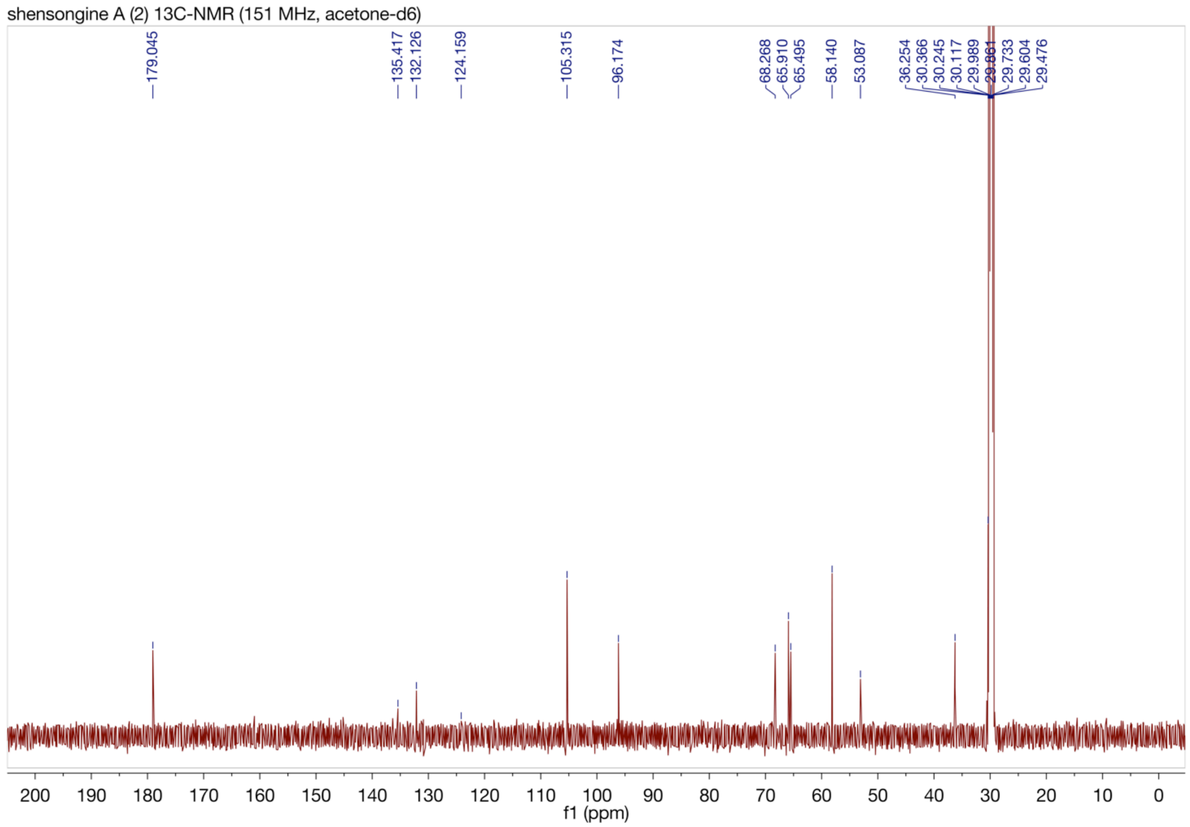
shensongine A (2) 13C-NMR (151 MHz, acetone-d6)
f1 (ppm)

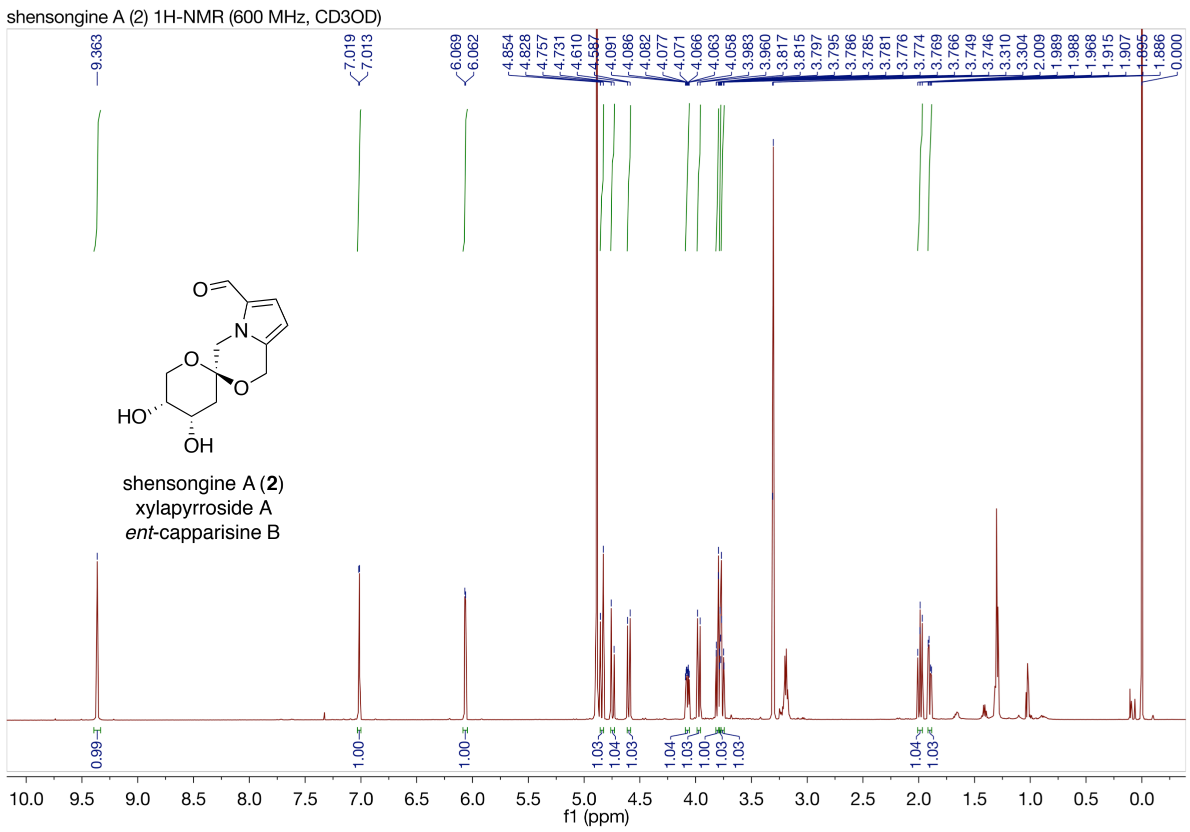
shensongine A (2) 1H-NMR (600 MHz, CD3OD)
9.363
7.019
7.013
6.069
6.062
4.854
4.828
4.757
4.731
4.610
4.587
4.091
4.086
4.082
4.077
4.071
4.066
4.063
4.058
3.983
3.960
3.817
3.815
3.797
3.795
3.786
3.785
3.781
3.776
3.774
3.769
3.766
3.749
3.746
3.310
3.304
2.009
1.989
1.988
1.968
1.915
1.907
1.895
1.886
0.000
O
N
O
O
HO
OH
shensongine A (**2**)
xylapyrroside A
ent-capparisine B
0.99
1.00
1.00
1.03
1.04
1.03
1.04
1.03
1.00
1.03
1.03
1.04
1.03
f1 (ppm)

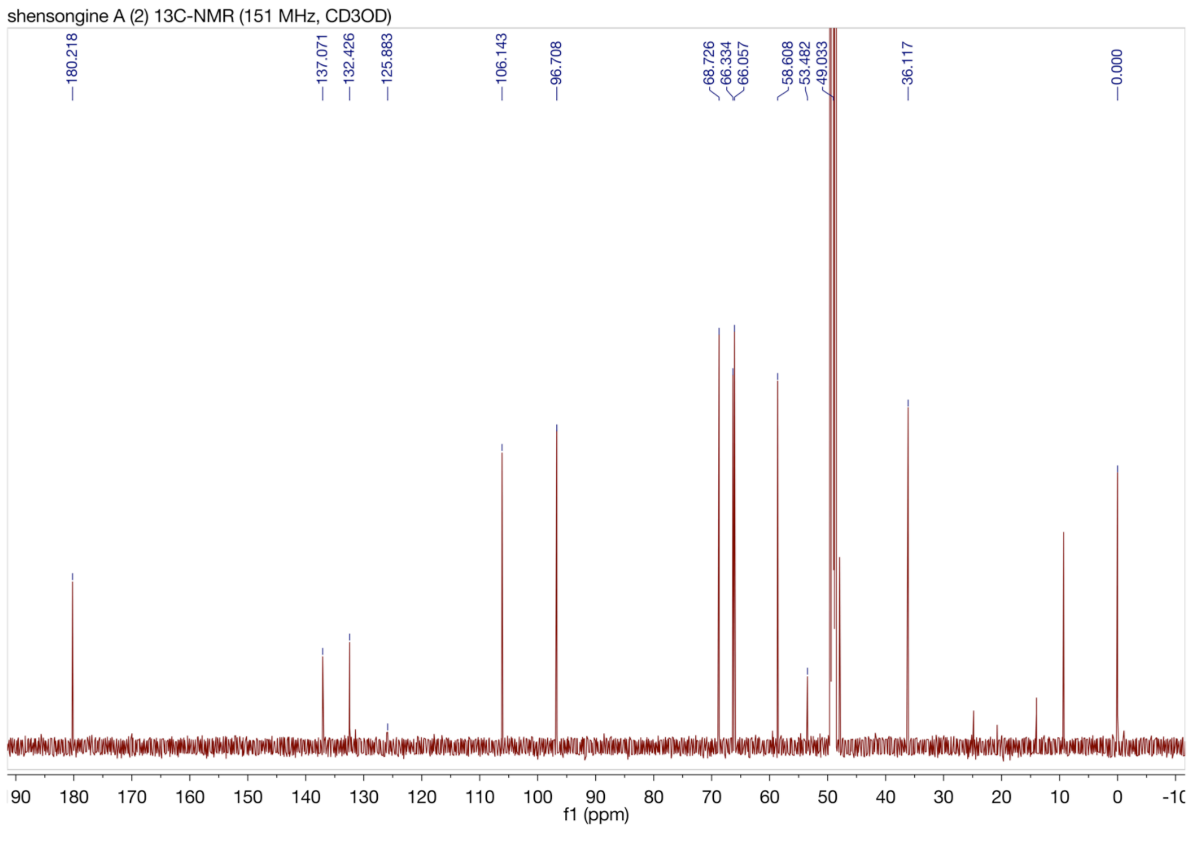
shensongine A (2) 13C-NMR (151 MHz, CD3OD)
180.218
137.071
132.426
125.883
106.143
96.708
68.726
66.334
66.057
58.608
53.482
49.033
36.117
0.000
f1 (ppm)

shensongine A (2) 1H-NMR (600 MHz, CDCl3)

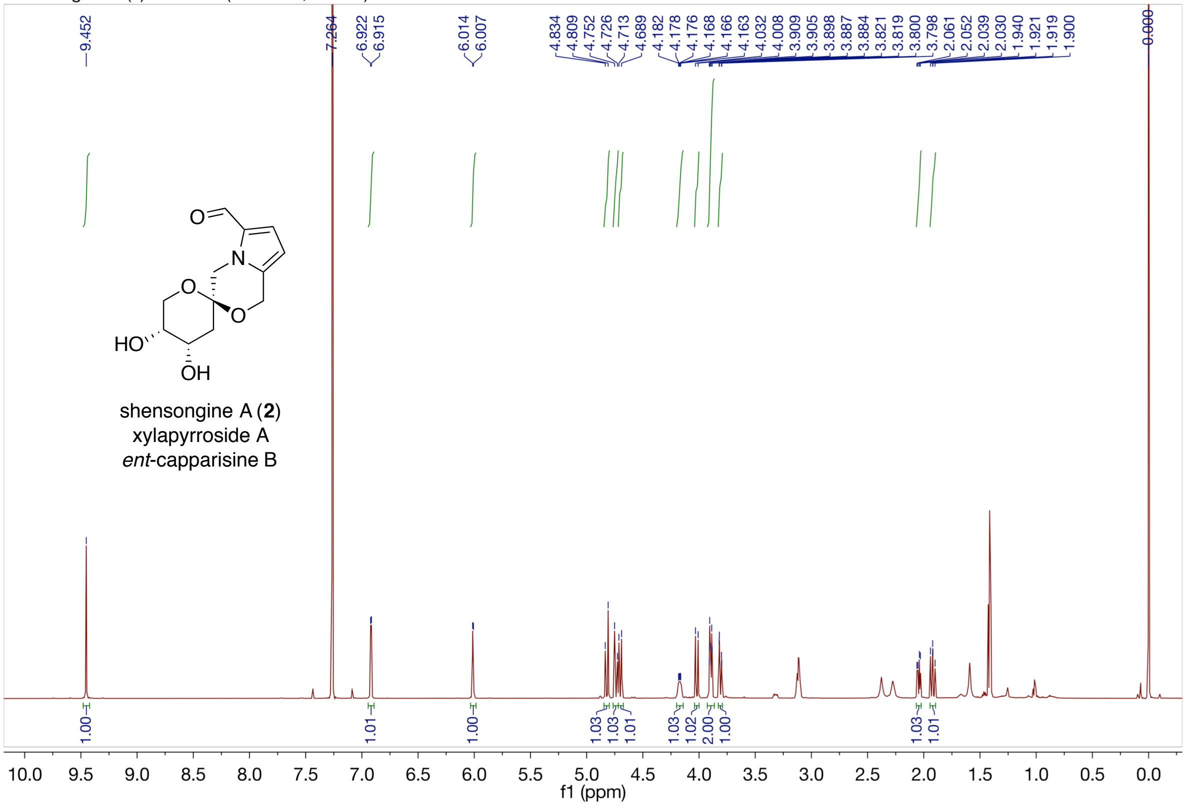

shensongine A (2) 13C-NMR (151 MHz, CDCl3)

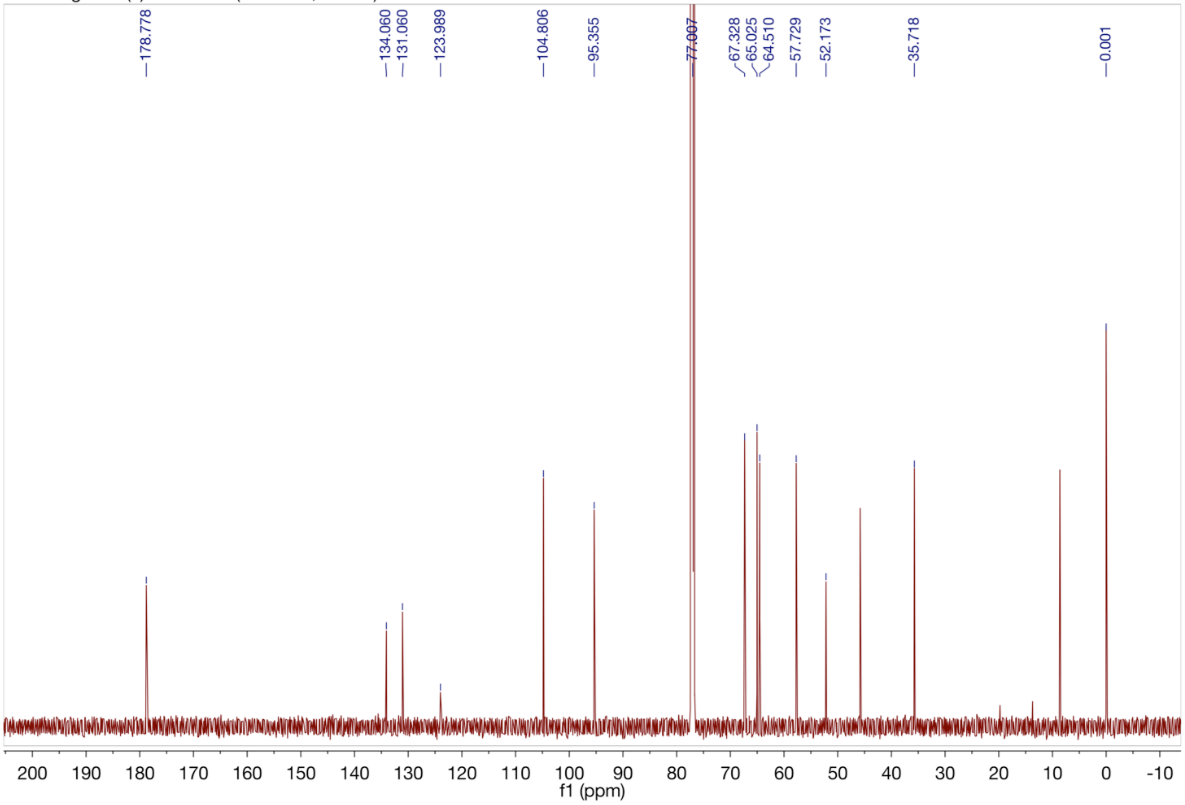

TIPS-protected 2-OH α-spiroketal (24) 1H-NMR (600 MHz, CDCl3)

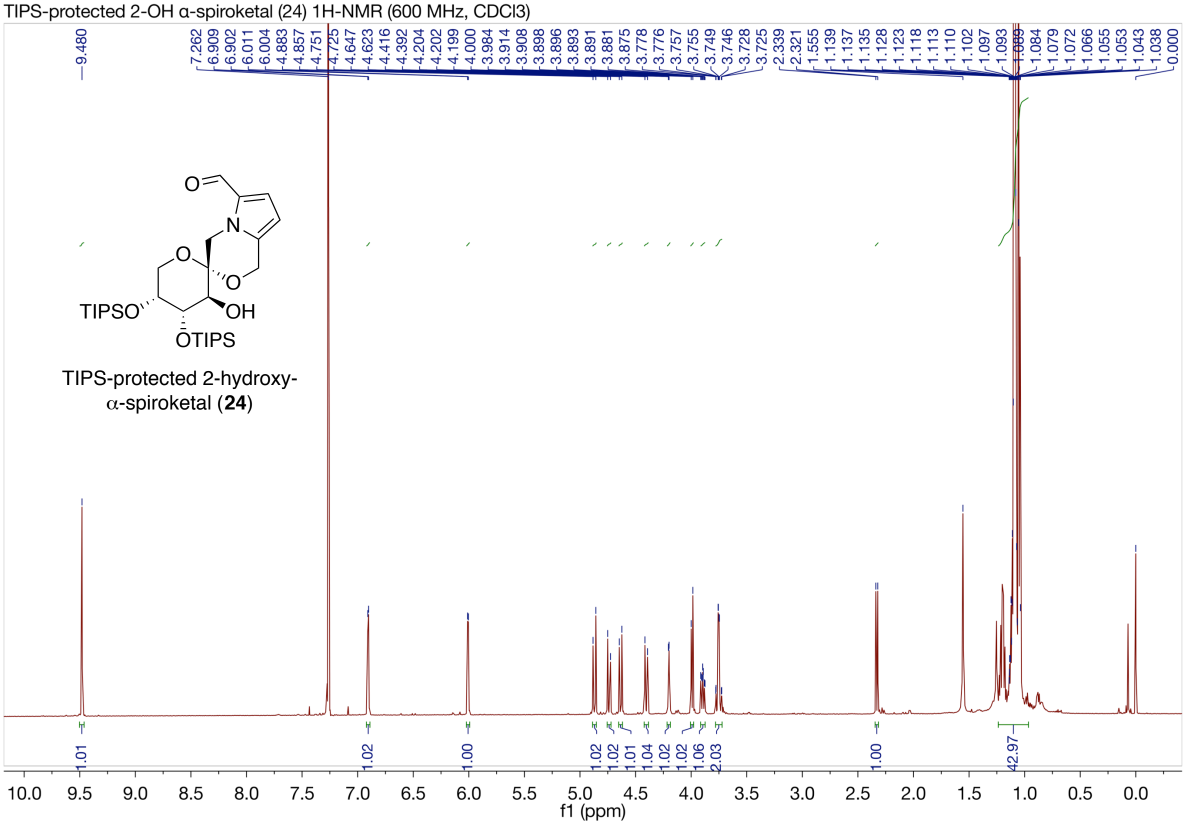

TIPS-protected 2-OH α-spiroketal (24) 13C-NMR (151 MHz, CDCl3)

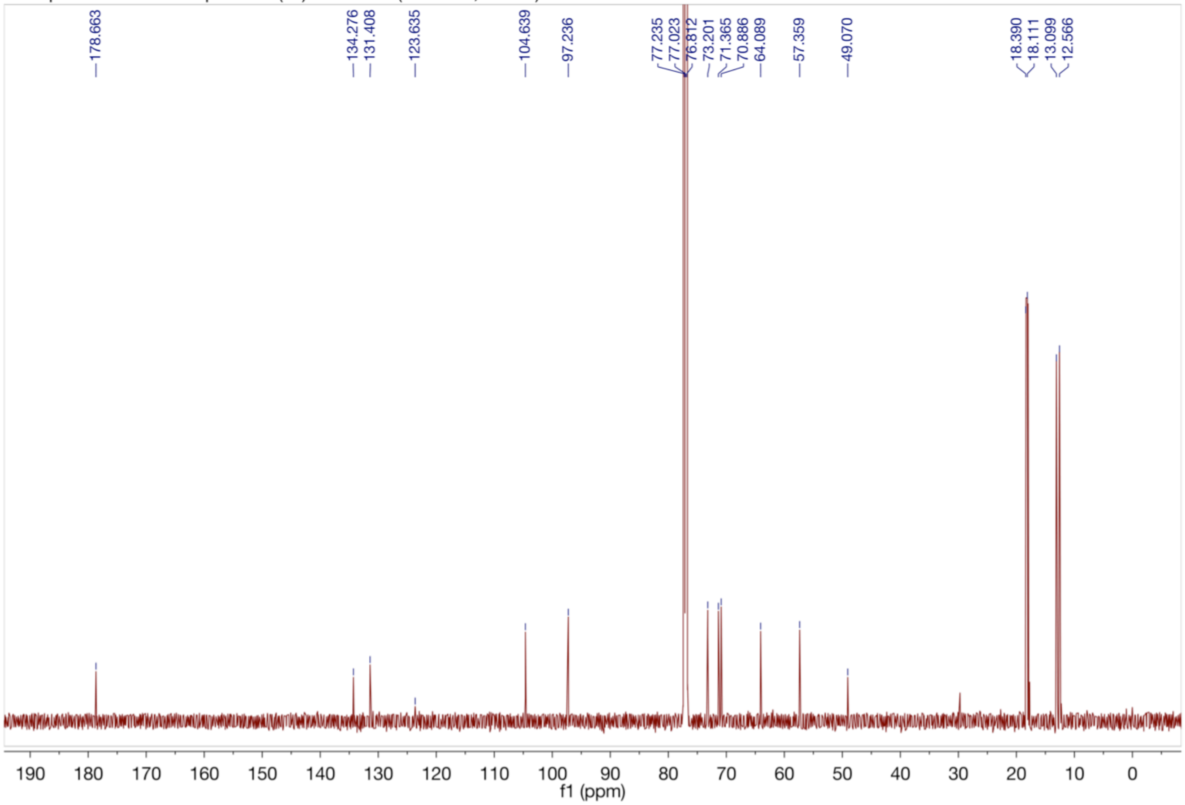

TIPS-protected 2-xanthate-α-spiroketal (26) 1H-NMR (600 MHz, CDCl3)

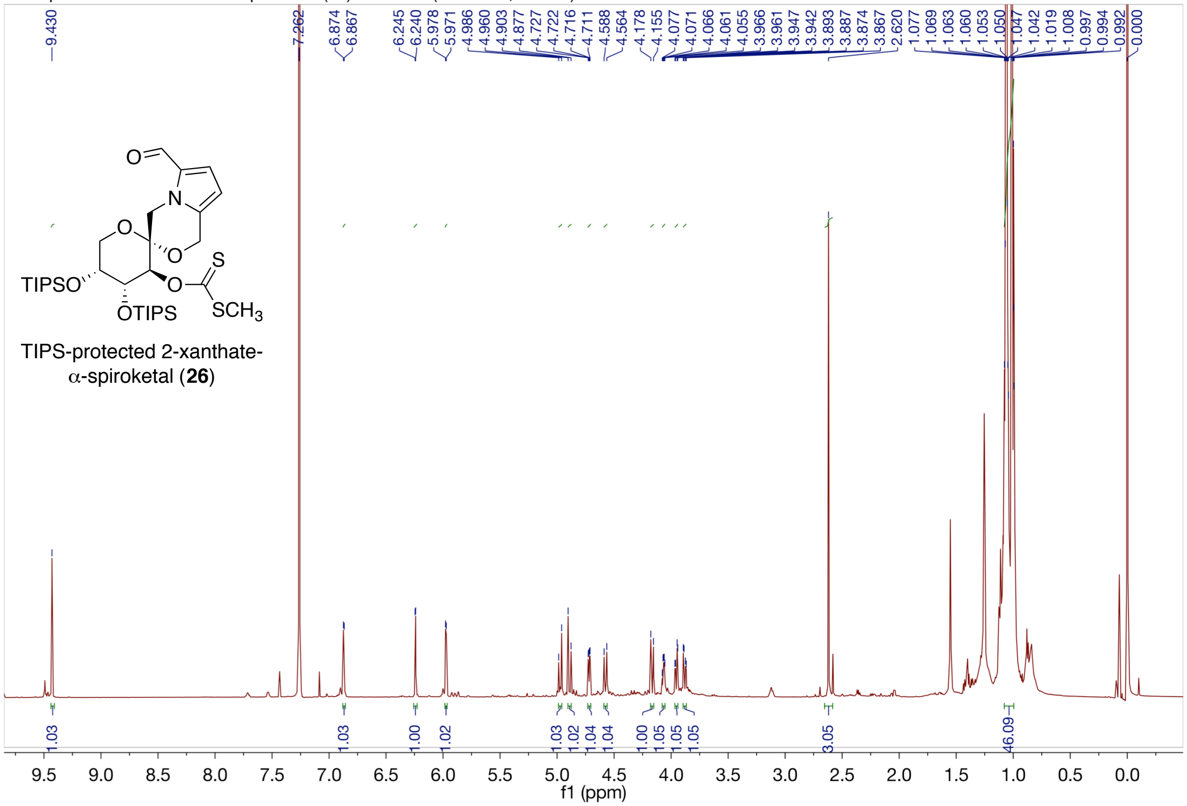

TIPS-protected 2-xanthate-α-spiroketal (26) 13C-NMR (151 MHz, CDCl3)

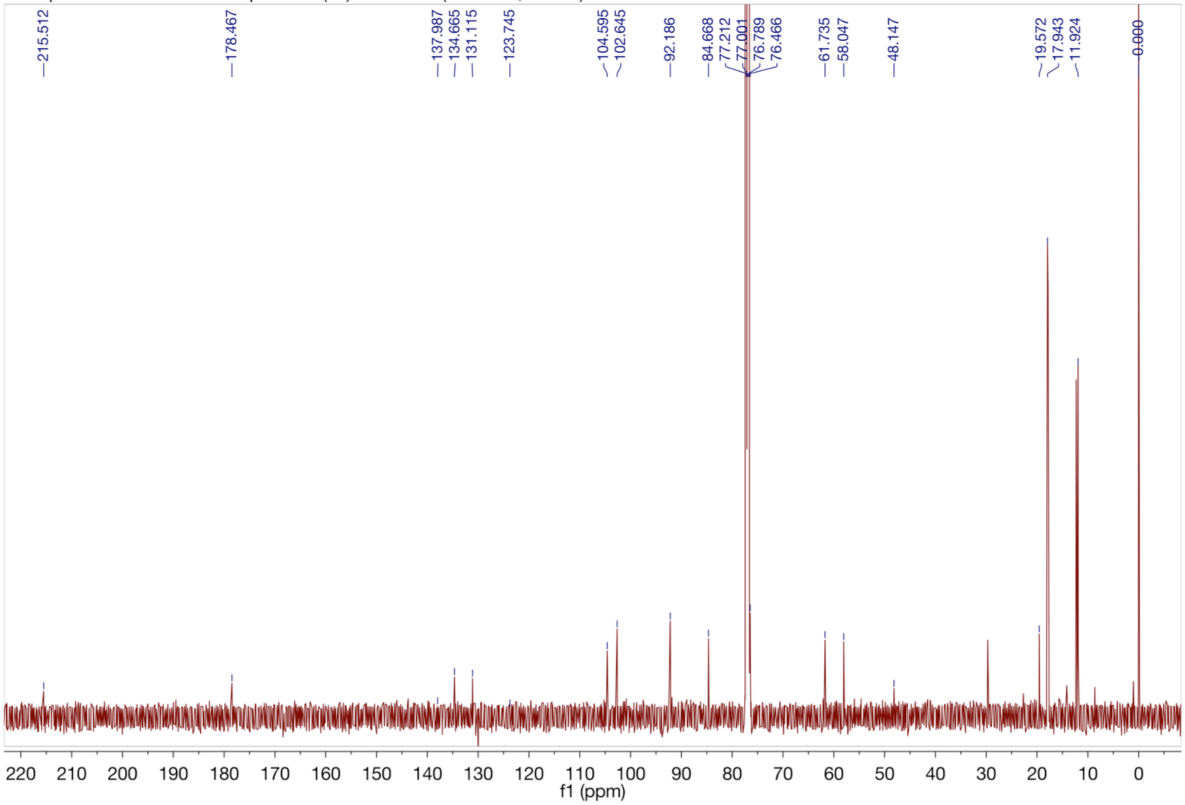

TIPS-protected α-spiroketal (23) 1H-NMR (600 MHz, CDCl3)

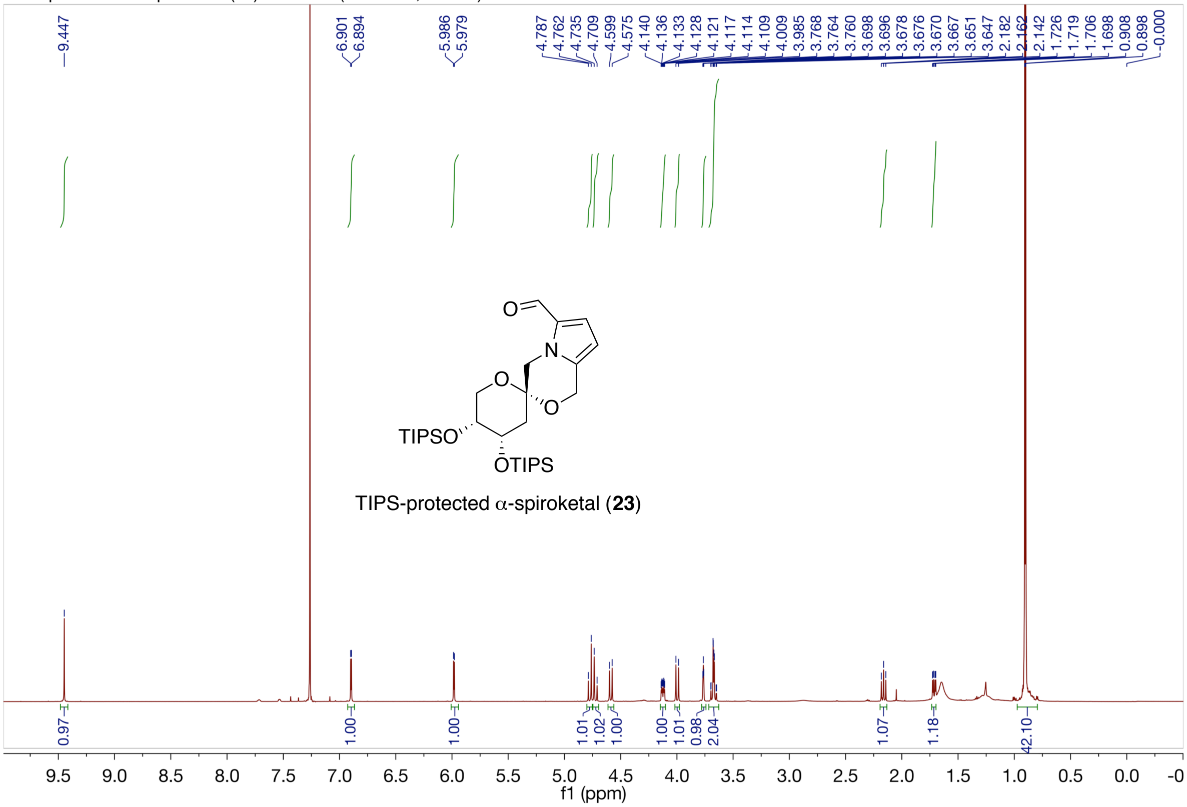

TIPS-protected α-spiroketal (23) 13C-NMR (151 MHz, CDCl3)

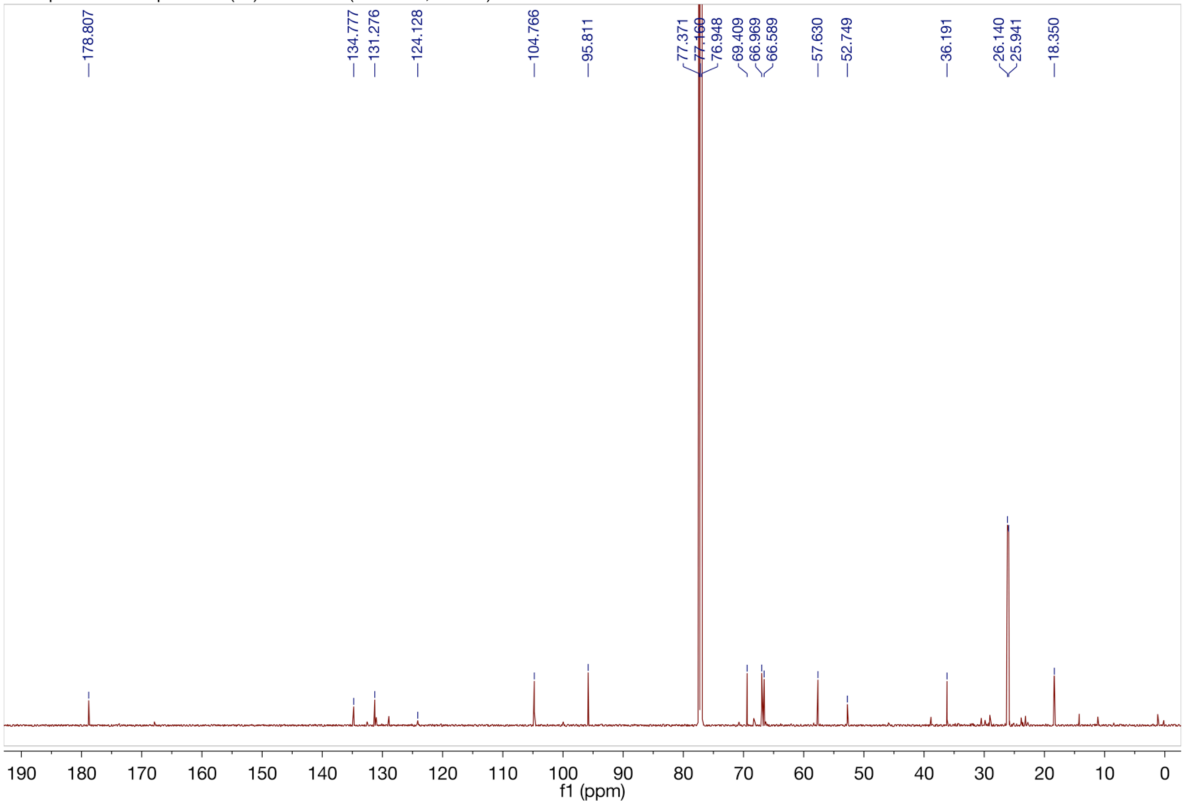

pollenopyrroside A (1) 1H-NMR (600 MHz, acetone-d6)

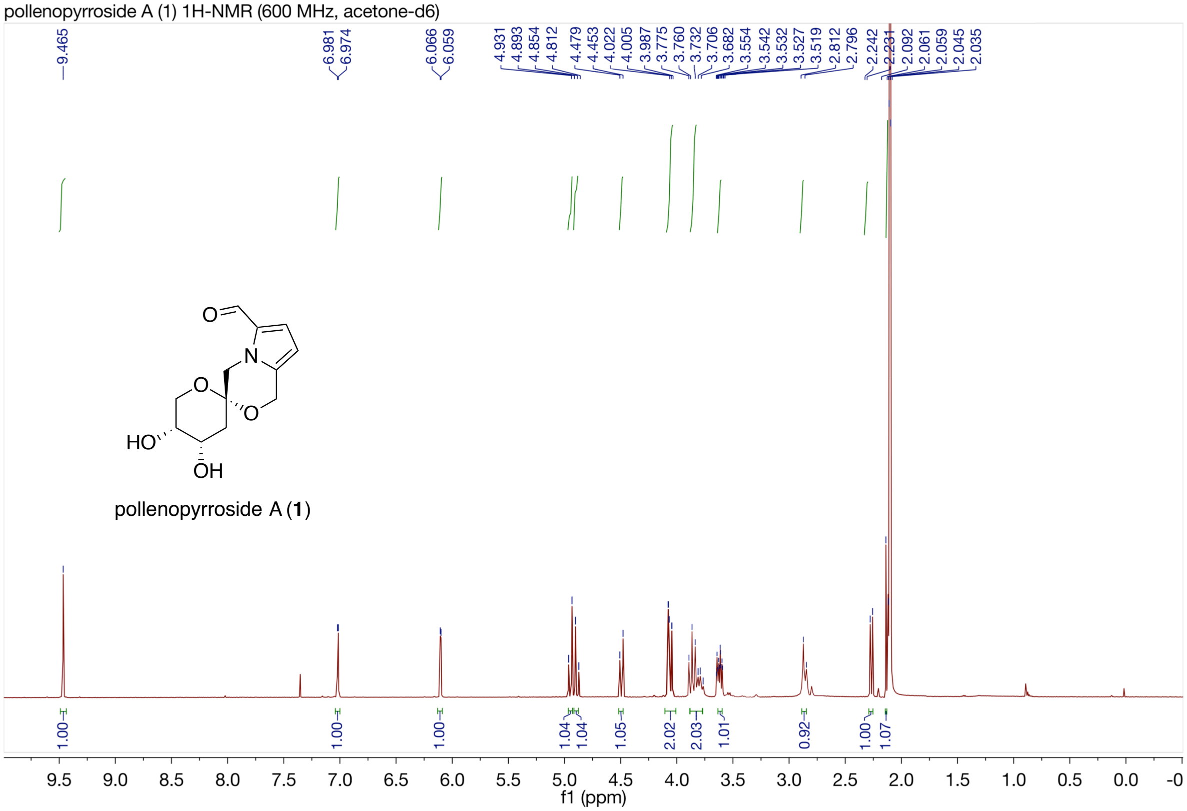

pollenopyrroside A (**1**)

pollenopyrroside A (1) 13C-NMR (151 MHz, acetone-d6)

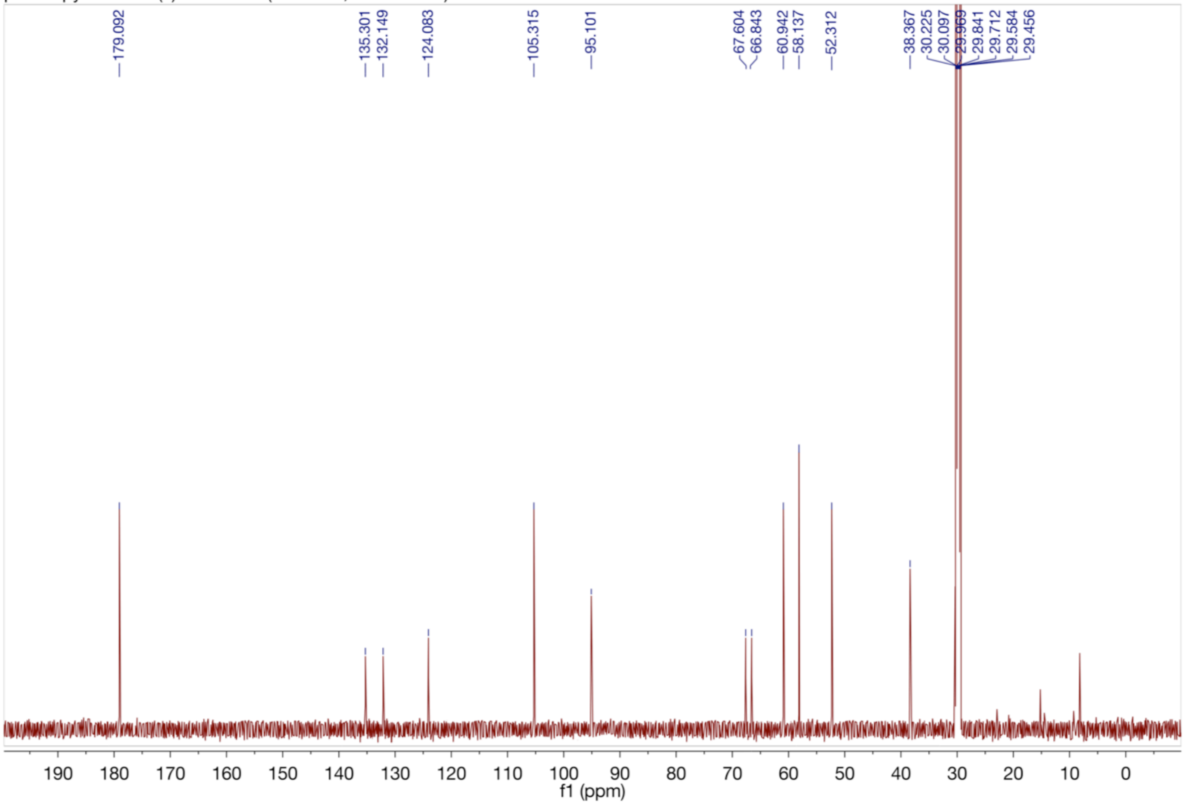

2-OH pollenopyrroside A (25) 1H-NMR (600 MHz, acetone-d6)

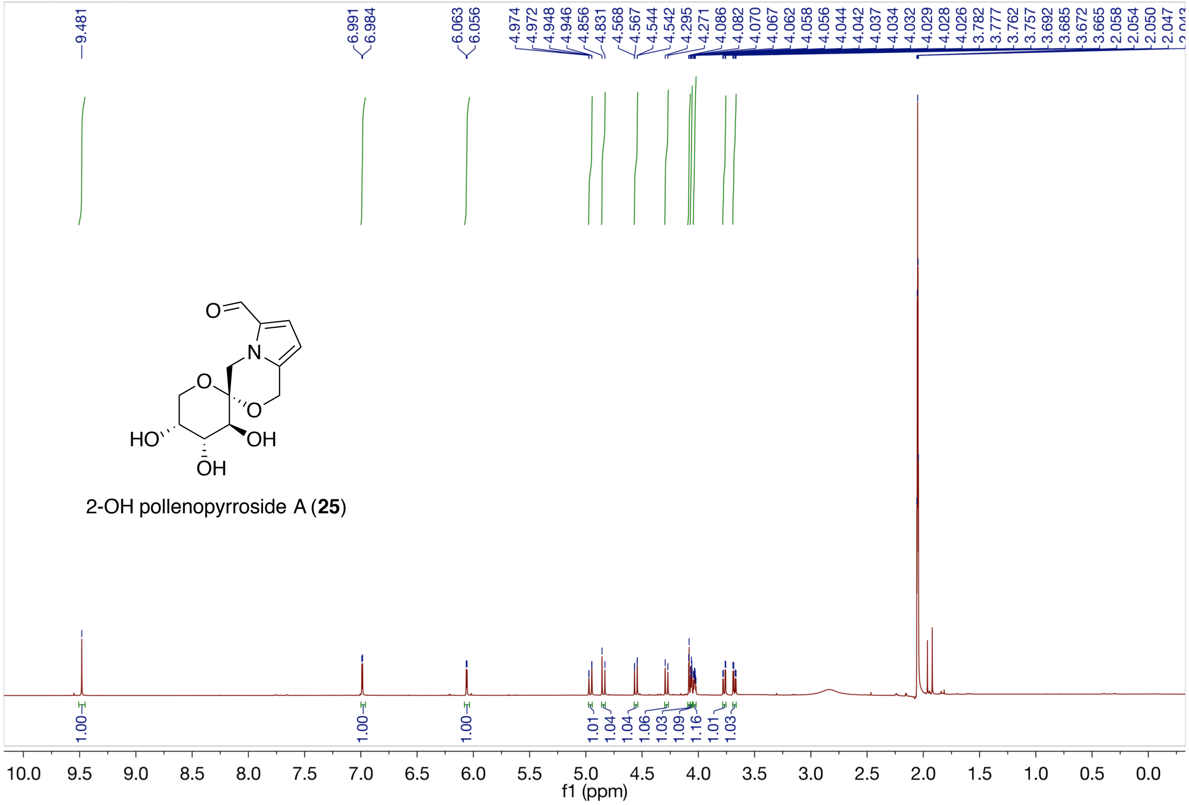

2-OH pollenopyrroside A (25) 13C-NMR (151 MHz, acetone-d6)

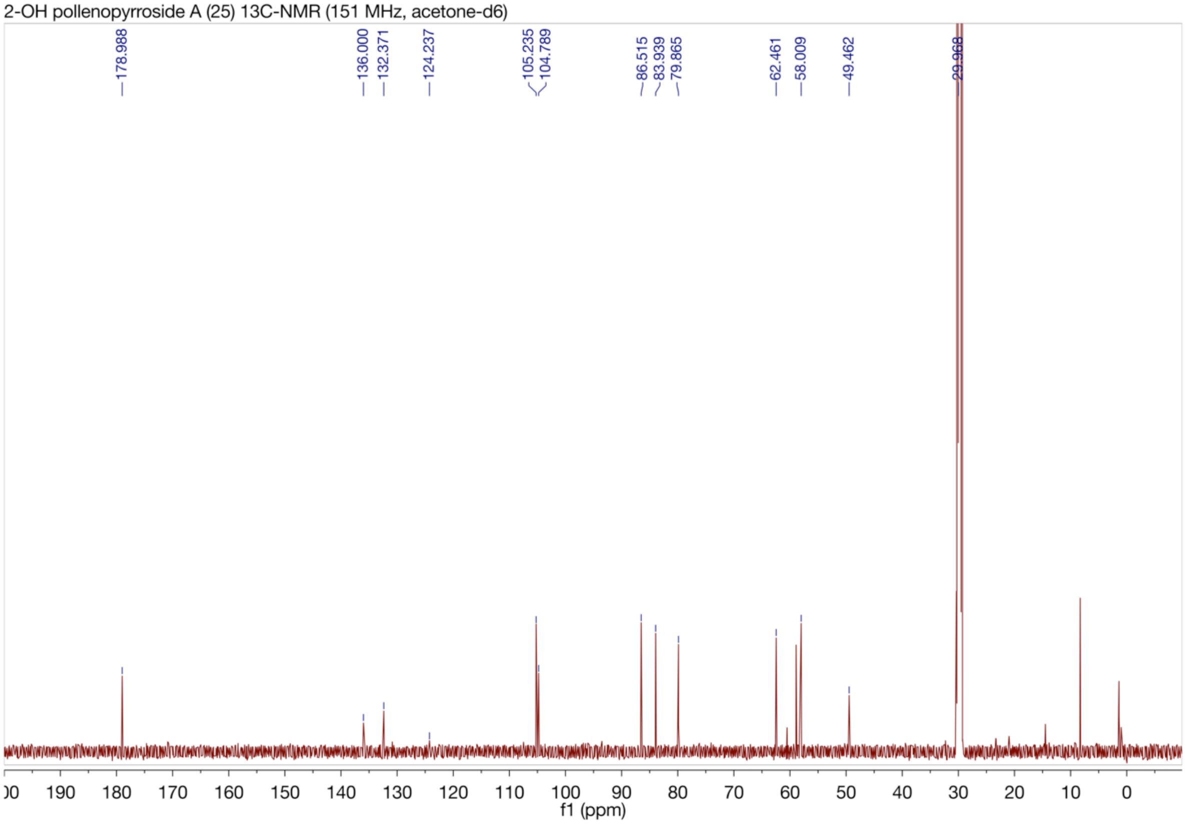

TIPS-protected 2-OH β-spiroketal (27) 1H-NMR (600 MHz, CDCl3)

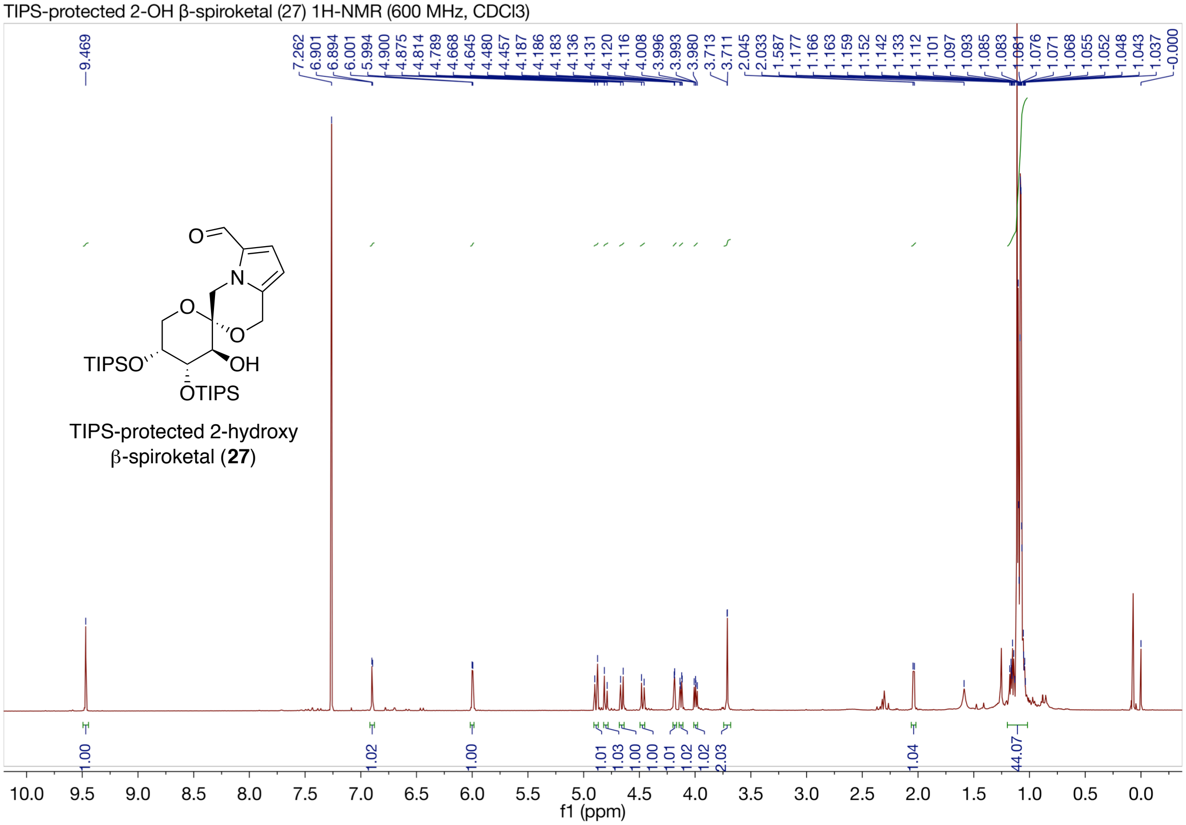

TIPS-protected 2-OH β-spiroketal (27) 13C-NMR (151 MHz, CDCl3)

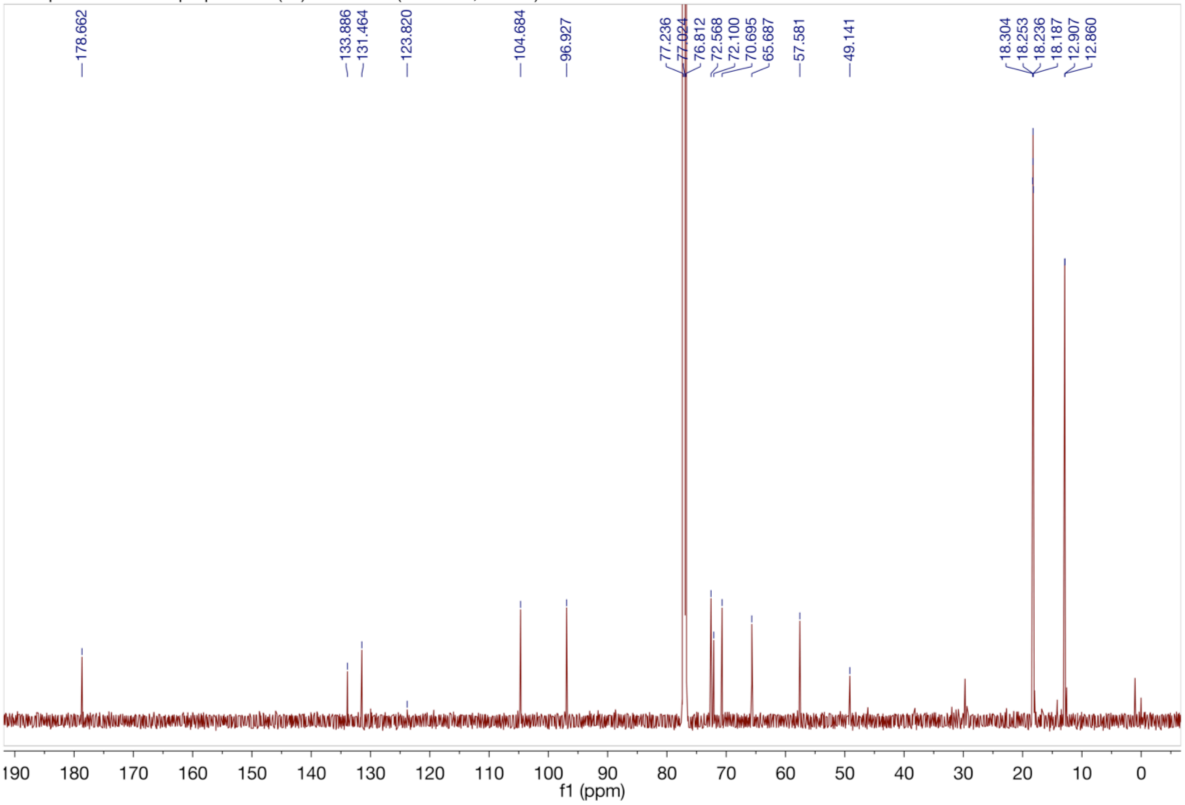

2-OH shensongine A (28) 1H-NMR (600 MHz, acetone-d6)

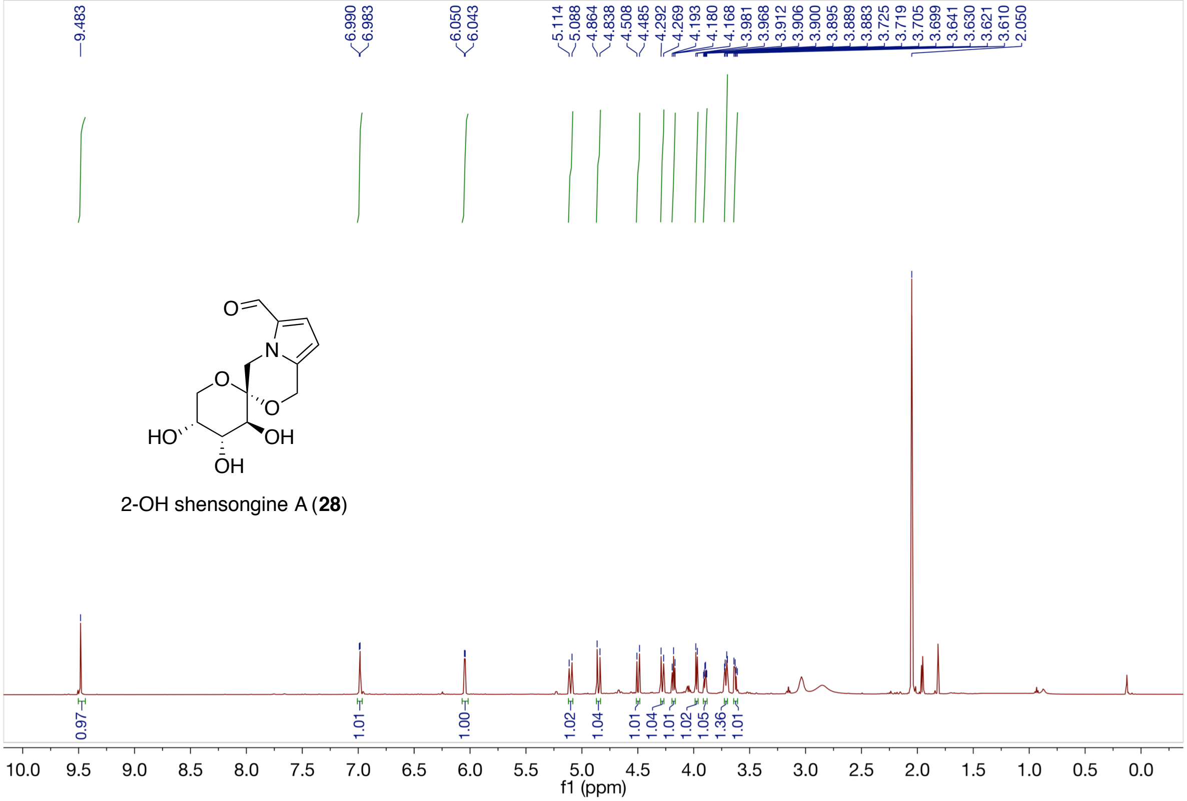

2-OH shensongine A (28) 13C-NMR (151 MHz, acetone-d6)

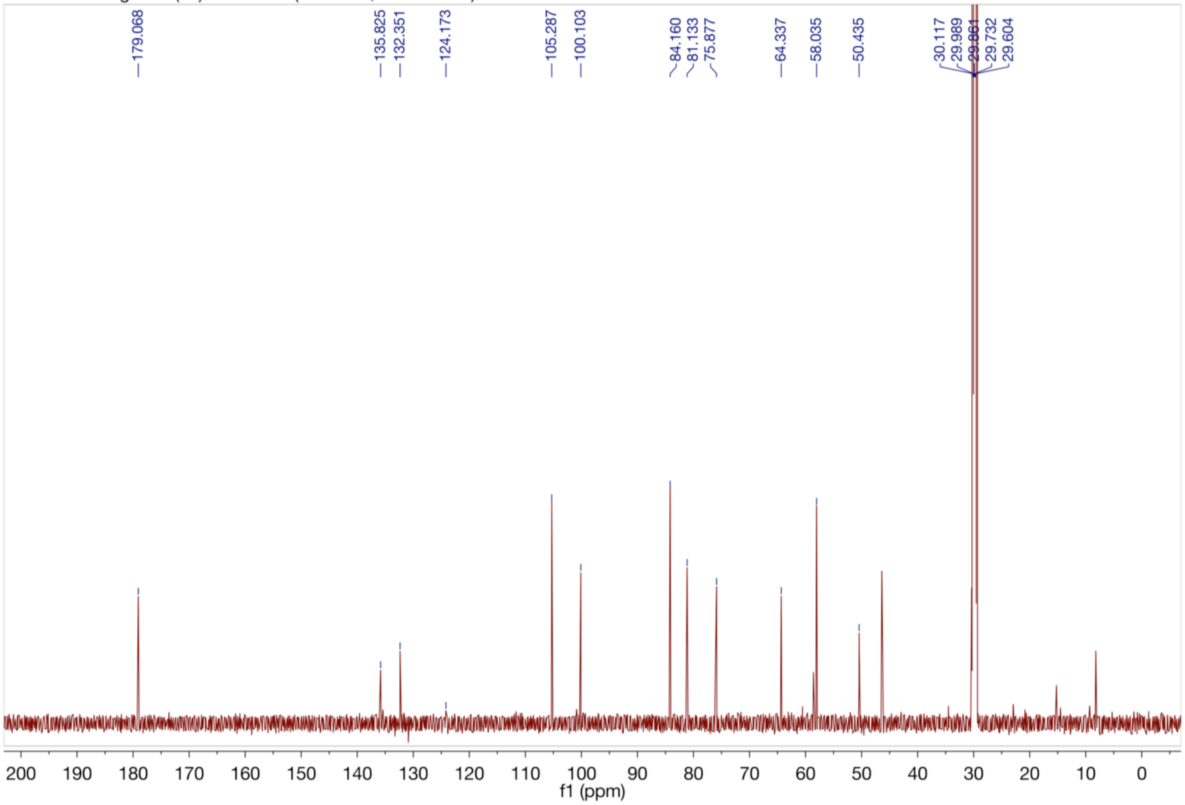

Supplement: Supplementary file 1 [file SC-008-C6SC05505B-s001.pdf]
